# Electroencephalography during acute painful procedures in neonates: a scoping review

**DOI:** 10.1097/PR9.0000000000001437

**Published:** 2026-04-09

**Authors:** Patricia Y. Gunawan, Luke Baxter, Maria M. Cobo, Marianne van der Vaart, Samyuktha Iyer, Vaneesha Monk, Kanwaljeet J.S. Anand, Charles B. Berde, Caterina Coviello, Mohammad Reza Daliri, Guy Dumont, Vineta Fellman, Behnood Gholami, Caroline Hartley, Pierre Kuhn, Nathalie L. Maitre, Roshni C. Mansfield, Simon Marchant, Sofie Nilsson, Elisabeth Norman, Safa Talebi, Sonya Wang, Karel Allegaert, Jonathan M. Davis, Mark A. Turner, Robert M. Ward, Edress Darsey, James P. Sheppard, Aomesh Bhatt, John van den Anker, An N. Massaro, Kanwaljit Singh, Rebeccah Slater

**Affiliations:** a Department of Paediatrics, University of Oxford, Oxford, United Kingdom; b Department of Pediatrics, Faculty of Medicine, Pelita Harapan University, Tangerang, Indonesia; c Colegio de Ciencias Biologicas y Ambientales, Universidad San Francisco de Quito USFQ, Quito, Ecuador; d Stanford Child Wellness Lab, Maternal & Child Health Research Institute, Stanford University School of Medicine, Stanford, CA, USA; e Department of Pediatrics, Stanford University School of Medicine, Stanford, CA, USA; f Department of Anesthesiology, Perioperative and Pain Medicine, Stanford University School of Medicine, Stanford, CA, USA; g Department of Anesthesiology, Critical Care and Pain Medicine, Boston Children's Hospital, Harvard Medical School, Boston, MA, USA; h Department of Anaesthesia, Harvard Medical School, Boston, MA, USA; i Division of Neonatology, Careggi University Hospital of Florence, Florence, Italy; j Biomedical Engineering Department, School of Electrical Engineering, Iran University of Science and Technology, Tehran, Iran; k Department of Electrical & Computer Engineering, The University of British Columbia, Vancouver, Canada; l British Columbia Children's Hospital Research Institute, Vancouver, Canada; m Paediatrics, Department of Clinical Sciences, Lund University, Skåne University Hospital, Lund, Sweden; n Children's Hospital, University of Helsinki, Helsinki, Finland; o Autonomous Healthcare, Santa Clara, CA, USA; p Department of Neonatology, University of Strasbourg, Strasbourg, France; q Department of Pediatrics, Emory University School of Medicine, Atlanta, GA, USA; r Children's Healthcare of Atlanta Inc, Atlanta, GA, USA; s Department of Neonatology, Skåne University Hospital, Lund, Sweden; t Faculty of Electrical and Computer Engineering, University of Tabriz, Tabriz, Iran; u Departments of Neurology and; v Pediatrics, University of Minnesota Medical School, Minneapolis, MN, USA; w Department of Development and Regeneration, KU Leuven, Leuven, Belgium; x Department of Pharmaceutical and Pharmacological Sciences, KU Leuven, Leuven, Belgium; y Department of Hospital Pharmacy, Erasmus Medical Center, Rotterdam, Netherlands; z Department of Pediatrics, Tufts Medical Center, Boston, MA, USA; aa Department of Women's and Children's Health, University of Liverpool, Liverpool, United Kingdom; ab Conect4children Stichting, Utrecht, Netherlands; ac Department of Pediatrics, University of Utah School of Medicine, Salt Lake City, UT, USA; ad Pediatric Development Strategy, Clinical Research Group, Thermo Fisher Scientific, Waltham, MA, USA; ae Nuffield Department of Primary Care Health Sciences, University of Oxford, Oxford, United Kingdom; af Center for Translational Research, Children's National Hospital, Washington, DC, USA; ag Office of Pediatric Therapeutics, Food and Drug Administration (FDA), Silver Spring, MD, USA; ah INC, Critical Path Institute (C-Path), Tucson, AZ, USA

**Keywords:** Pain, EEG, Neonate, Evidence synthesis

## Abstract

Although electroencephalography (EEG) is used to assess neonatal pain in research settings, EEG assessments have not been sufficiently characterized for use as end points to assess the efficacy of analgesics in regulatory-endorsed, industry-sponsored trials. We aimed to identify all studies conducted in neonates with EEG recordings during acute somatic nociceptive skin-breaking procedures, and to create a network of authors who will be invited to contribute their individual participant data (IPD) to an IPD meta-analysis to establish the validity, reliability, and clinical interpretability of an EEG-based neonatal pain measure. To identify literature, we searched MEDLINE, Embase, CINAHL, Web of Science, Scopus, Google Scholar, ClinicalTrials.gov, and the WHO ICTRP from database inception to July 2, 2025. Eligible studies were primary empirical studies that included neonates with EEG recordings during acute skin-breaking procedures. We identified 55 studies across 11 countries. Heel lance was the most common painful procedure; others included venipuncture, immunization, and lumbar puncture. The impact of 12 analgesic interventions has been studied to date, mostly nonpharmacological interventions. Individual-electrode EEG is more common than EEG caps. We noted relatively high data loss due to EEG data-quality concerns. A wide range of non-EEG pain-relevant measures have been recorded alongside EEG (eg, behavior, vital signs). Coauthorship network analysis highlighted that authors commonly work within discrete authorship hubs, with limited coauthorship across hubs. The predominance of studies was from European and American institutions, which limits generalizability. We conclude that sufficient data are available to undertake an IPD meta-analysis.

## Abstract 2

Supplemental Digital Content is Available in the Text.

The authors review use of electroencephalography globally to measure acute neonatal pain, initiate collaborations with key researchers in the field, and identify data for subsequent meta-analysis.

## 1. Introduction

Neonatal pain assessment is extremely challenging. This is due to (1) the subjective nature of painful experiences, (2) the nonverbal nature of neonates, and (3) a lack of specificity of behavioral and physiological responses to painful and nonpainful stimuli.^75^ Development of clinical pain scales for neonates and infants has been ongoing since at least the late 1980s, with more than 40 different versions currently available to both the clinician and the researcher.^15,58^ Many of these pain scales rely on more than one measurement “domain,” such as a combination of measures from the behavioral (eg, facial expressions, limb movements), the cardiovascular (eg, heart rate, blood pressure), or the respiratory (eg, breathing pattern, oxygen requirement) domains. However, over the past decade, there has been increased interest among both researchers^11,86^ and regulatory bodies^36,88^ in the development of brain-based indicators of neonatal pain for use as a primary end point in clinical trials or as a component of a multidimensional neonatal pain scale.

Currently, brain-based indicators are not included in neonatal pain scales. This is likely due to both limited access to brain-based equipment for monitoring or evaluating pain responses and the lack of well-established validity, reliability, and clinical interpretability of these measures.^2,3^ Owing to these concerns and the desire to advance clinical pain scale content validity,^26^ we are undertaking an individual participant data (IPD) meta-analysis of an electroencephalography (EEG) metric.^9^ The overarching goal is to facilitate the creation of an EEG-based acute pain pharmacodynamic biomarker for use in neonates.^9^ We are specifically interested in acute somatic nociceptive pain, where pain is defined as an unpleasant sensory and emotional experience associated with, or resembling that associated with, actual or potential tissue damage.^51^ We operationalize acute pain as acute somatic skin-breaking procedures (eg, heel lance procedures), which is in line with acute pain models for clinical trials in the neonatal population recommended by the ACTTION (Analgesic, Anesthetic, and Addiction Clinical Trial Translations, Innovations, Opportunities, and Networks) Pediatric Pain Research Consortium.^106^ Compared with standard summary data meta-analyses, an IPD meta-analysis enables the ability to perform more powerful and nuanced analysis of the source data, while facilitating the analysis of larger sample sizes than in individual studies.^78^ Furthermore, this approach will enable the implementation of standardized analytic techniques to establish the measurement properties of an EEG-based acute pain biomarker.

As an initial step toward conducting the IPD meta-analysis, we scoped existing literature to identify researchers who have recorded EEG in neonates during acute skin-breaking procedures and who may have relevant individual neonatal EEG data that could contribute to the IPD meta-analysis. Our intention is to create a collaborative network of researchers sharing data to empower others to develop novel EEG-based methods and outcomes. In this current scoping review, we have (1) summarized the relevant literature, (2) invited investigators who conducted relevant studies to coauthor the review and provide expertise and insights on factors affecting data quality and study design, and (3) developed a standardized and objective approach to facilitate future collaborative efforts.

We identified the primary empirical research conducted on acute neonatal pain that featured EEG as a recording method. We report on where and when this research has been conducted and catalog researchers working in this field. We summarize the: (1) sample sizes of the studies that collected EEG data, (2) age and sex distributions of study participants, (3) nature of the collected EEG data, (4) painful procedures that were investigated, and (5) pain relief interventions that have been studied using EEG outcomes to date. To understand the heterogeneity in collected EEG data and the potential for implementing a standardized analytic approach as part of the IPD meta-analysis, we also document the electrode placement methods and positions. To address concerns about data quality when using neonatal EEG for pain research, we quantify the amount of data reportedly lost due to EEG data quality issues. Finally, we record non-EEG pain assessment measures that were collected alongside the EEG data, including clinical pain scales, vital signs measurements, and pain behavior videos.

## 2. Methods

### 2.1. Research question

We aimed to identify all published research on pain in neonates assessed using EEG and bring together this research community. This scoping review is the initial stage of an IPD meta-analysis designed to assess the reliability, validity, and clinical interpretability of a specific EEG outcome measure.^9^ The associated IPD meta-analysis research question is “Is the specific EEG-recorded brain activity magnitude metric under investigation, that is evoked by acute somatic nociceptive skin-breaking procedures in neonates 34–44 weeks postmenstrual age (PMA), a reliable, valid, and interpretable pain-relevant indicator?” The eligibility criteria (see below) are the same in this scoping review as in the IPD meta-analysis, except we do not restrict the participants' age to 34 to 44 weeks PMA. Instead, the entire neonatal period is included in this scoping review. The neonatal period for term and post-term neonates is defined as the day of birth plus 27 days; the neonatal period for preterm neonates is defined as the day of birth through the expected date of delivery plus 27 days.^30,31^ The search strategies (see below) are identical between the IPD meta-analysis and the scoping review.

Because this scoping review is the first stage of the IPD meta-analysis, the research question is similar to the IPD meta-analysis research question. The scoping review research question in PICO (Population, Intervention, Comparator, Outcome) format includes (1) human neonates as population, (2) acute somatic nociceptive skin-breaking procedure as “intervention” ie, painful procedure, (3) comparator is not applicable, and (4) EEG measures of procedure-evoked brain activity. The research question is “What research has been published examining acute somatic nociceptive skin-breaking procedures in human neonates, assessed using EEG?”

### 2.2. Protocol development

This scoping review is a subcomponent of the IPD meta-analysis protocol, which has been written according to PRISMA-P (Preferred Reporting Items for Systematic Reviews and Meta-Analyses-Protocol extension) guidelines,^67^ has been pre-registered on PROSPERO (International Prospective Register of Systematic Reviews) on July 14, 2023 (CRD42023444809), and has subsequently been published in a peer-reviewed journal.^9^ No data extraction for this scoping review occurred before review registration on PROSPERO.

### 2.3. Eligibility criteria

We provide a full list of our eligibility criteria in the Supplementary Information (Supplementary Table 1, http://links.lww.com/PR9/A400). We included primary empirical research studies, including gray literature, with any study design and without any restriction related to publication date or language. We excluded secondary literature (eg, reviews, book chapters) and nonempirical publications (eg, commentaries, opinions, perspectives).

### 2.4. Literature searches

We searched 6 bibliographic databases: MEDLINE (Ovid), Embase (Ovid), CINAHL (EBSCO Industries), Web of Science Core Collection (Clarivate Analytics), Scopus (Elsevier), and Google Scholar (Publish or Perish). We searched the clinical trial registry ClinicalTrials.gov (https://clinicaltrials.gov) and the clinical trial registry platform WHO ICTRP (World Health Organization International Clinical Trials Registry Platform; https://trialsearch.who.int), which includes 18 registries. Finally, we included publications known to the authors. All databases and trial registry platforms were searched from inception to July 2, 2025.

For electronic searches, the search strategy was initially developed in MEDLINE, then translated for other databases and registries. The search focused on the combination of 3 core topics: “neonate,” “pain,” and “EEG.” The search strategies were independently peer-reviewed using the PRESS Checklist by an Outreach Librarian at the Bodleian Health Care Libraries, University of Oxford.^63^ All search strategies are presented in full in the Supplementary Information, http://links.lww.com/PR9/A400.

We used EPPI-Reviewer (Evidence for Policy and Practice Information and Co-ordinating Reviewer) for deduplication and screening.^97^ Study selection was a 2-stage process: screening on title and abstract followed by screening on full text. Screening was performed in duplicate by 2 independent reviewers and disagreements settled by discussion between both reviewers.

### 2.5. Data extraction and variable explanation

Data extraction was performed by one author and then verified by a second author, with disagreements settled by discussion. The data extraction and synthesis were only performed on a single representative publication per research study. In instances where a study had multiple associated publications (eg, one clinical trial had a trial registration, a protocol paper, a statistical analysis plan paper, a main publication paper, and an additional trial report document), a single primary publication representative of the study was selected for data extraction and synthesis. We extracted data on publication year, country in which data were collected, authorship, sample size, average age of participants, sex ratio, skin-breaking procedures, pain-relief interventions, EEG electrode placements, EEG data quality issues, and non-EEG data. Explanations for each of these variables are provided in the Supplementary Information (Supplementary Table 2, http://links.lww.com/PR9/A400).

### 2.6. Data synthesis

We synthesized data descriptively; we did not perform inferential statistics or hypothesis testing. Results are presented primarily as figures. The researcher coauthorship network was produced using VOSviewer.^28^

## 3. Results

### 3.1. Study selection and study characteristics

**Figure 1.**
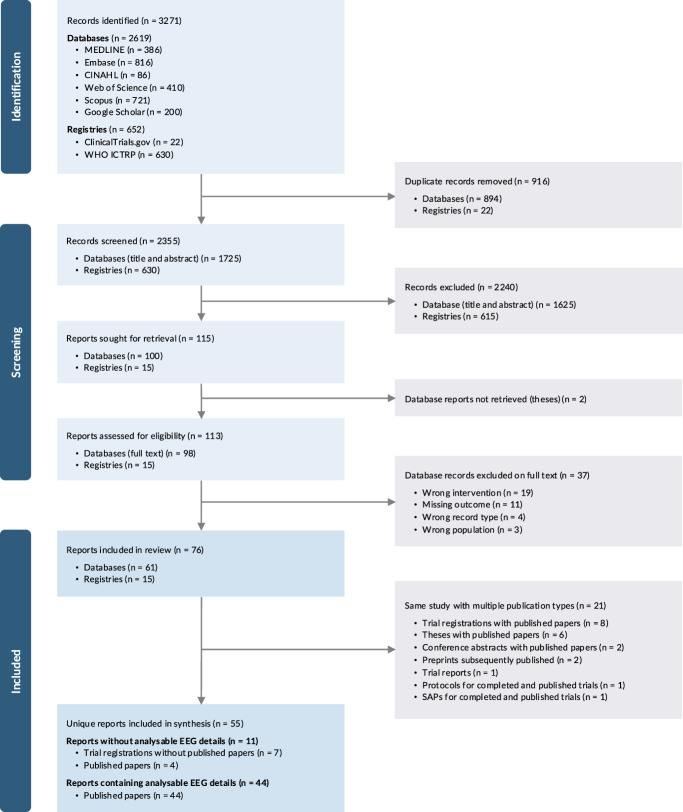
PRISMA flow diagram.

Of the 2,355 records screened, we identified 76 relevant to this review (Fig. 1). Twenty-one of 76 (26.9%) were directly related to the same study with multiple publication types (eg, clinical trial registrations for completed trials and published articles). In all cases, there was a clear primary publication, with additional publications excluded from synthesis including trial registrations, theses, conference abstracts, preprints, protocols, and statistical analysis plans. A list of the 21 nonprimary publications excluded from synthesis is provided in the Supplementary Table 3, http://links.lww.com/PR9/A400.^5,8,10,13,16,19,22,27,38,39,56,64,65,68,76,81,87,89,90,96,101^ A list of the 55 unique representative primary publications included in the synthesis is provided in the Supplementary Table 4, http://links.lww.com/PR9/A400.^1,4,6,12,14,17,18,20,21,23,24,32–35,37,40–47,50,53–55,57,60–62,69–74,77,79,80,84,85,91–93,95,99,100,103–105,107,109,110^

### 3.2. Results structure

Below, we first outline the geographical origin of the EEG data and the publication year of the relevant records for the 55 unique studies included in the synthesis. Second, we assess the coauthorship network of these studies, excluding the 7 trial registrations (as there is no authorship list for trial registrations), which resulted in a total of 48 studies for coauthorship network analysis. Third, we provide details on study populations of neonates, EEG sample sizes and acquisition details, painful procedures, analgesic interventions, and non-EEG outcomes, for all 44 studies containing analyzable EEG details. Eleven of 55 studies without analyzable EEG details included: (1) 7 trial registrations,^23,37,57,70,71,74,107^ (2) 3 methods development articles with vaguely detailed exemplar EEG data,^35,109,110^ and (3) one clinical trial protocol (Supplementary Table 4, http://links.lww.com/PR9/A400).^17^

### 3.3. Geographical and temporal distribution of research

**Figure 2.**
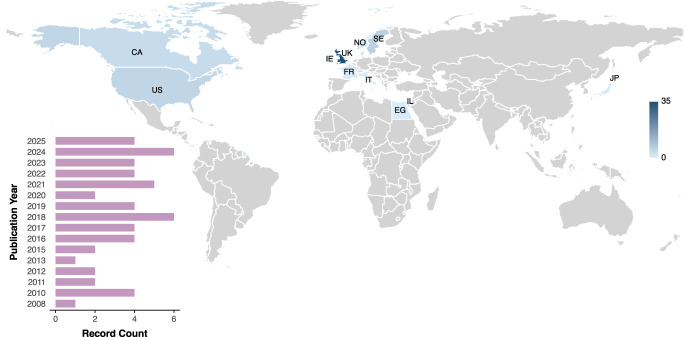
Country of origin of the data set and publication year of the published record. CA, Canada; EG, Egypt; FR, France; IE, Ireland; IL, Israel; IT, Italy; JP, Japan; NO, Norway; SE, Sweden; UK, United Kingdom; US, United States.

Of the 55 unique studies, most data were collected in Europe, with the United Kingdom (UK) producing the highest number of publications (n = 35, Fig. 2). We did not identify any data sets originating from South-East Asia or Africa (using WHO region definitions). The publication of research where EEG was used to study pain in neonates seems to have started in 2008 with a rate of publication of approximately 3 per year (Fig. 2).

### 3.4. Researcher coauthorship network

**Figure 3.**
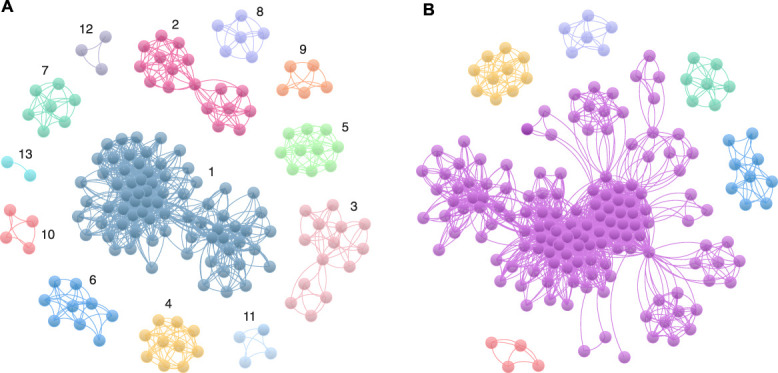
Researcher coauthorship networks. Nodes represent authors, edges indicate coauthorships. (A) Before this scoping review: the network comprises relatively unconnected “islands” of authors; clusters are labelled 1–13. See Supplementary Table 6, http://links.lww.com/PR9/A400 for the full author list by cluster. (B) Coauthorship network after this scoping review. Colors are consistent across panels for clusters that are unchanged; colors that appear in only one panel reflect clusters that merge into a larger connected network in the other panel.

From the 48 studies included in the coauthorship network analysis, we identified 13 unconnected coauthorship clusters (Fig. 3A, Supplementary Table 6, http://links.lww.com/PR9/A400). Once this scoping review is included in the list of studies, the coauthorship network connectivity increases with the number of unconnected clusters reducing to 6 (Fig. 3B).

### 3.5. Study populations

**Figure 4.**
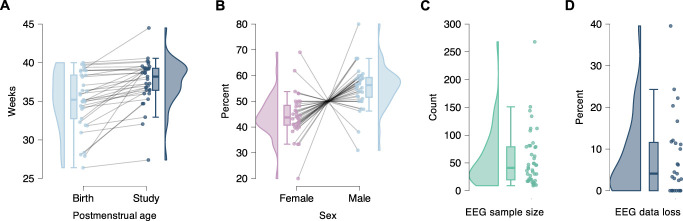
Study populations and EEG sample sizes. In each panel, points show study-level values, overlaid with half-violin density plots and boxplots (median and interquartile range). (A) Average postmenstrual age at birth and at study; grey lines connect paired birth and study values from the same study. (B) Sex distribution (female, male); grey lines connect paired female and male percentages within the same study. (C) EEG sample size: number of babies with EEG per study. (D) Percentage of EEG data lost due to quality issues per study.

To characterize the study populations of neonates studied using EEG during painful skin-breaking procedures, we recorded the average age at birth, average age at study (PMA in weeks), and percentage of female and male neonates—Figures 4A and B. Taken together, the age distributions indicate that the included studies are overweighted to near-term/term-equivalent infants (median age at birth 35.2 weeks; median age at study 38.2 weeks), with earlier preterm gestations underrepresented. Sex was likewise imbalanced, with a median 12.6% higher proportion of male than female neonates across studies.

### 3.6. Electroencephalography

The median EEG sample size (number of neonates with EEG recordings) across studies was n = 41, with a minimum of n = 9 and a maximum of n = 268 (Fig. 4C). The median percentage of neonates excluded from EEG analysis due to poor EEG data quality was 4.1%, with a minimum of 0% and a maximum of 39.6% (Fig. 4D). Reasons for poor EEG data quality included artifacts or loss of electrode contact with the scalp. Twenty-two of 44 studies (50%) either did not report on the topic of participant loss due to EEG data quality issues or did report it, but it was unclear how many participants were specifically lost due to poor data quality. Twenty-two studies described the method by which they determined low-quality EEG data for exclusion.

**Figure 5.**
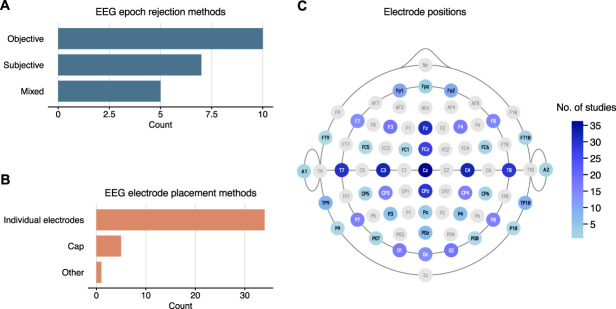
(A) EEG epoch rejection methods. (B) EEG electrode placement methods. (C) EEG electrode placement position frequency counts, mapped to the 10-10 system.

We identified 3 approaches reported for identifying low data quality: objective, subjective, and mixed approaches (Fig. 5A). The objective approach was most often used and involved explicitly objective criteria, which in most cases was an amplitude threshold above which the epoch was classified as artifactual and rejected. The amplitude threshold used among studies varied. The subjective approach was typically described as involving “expert assessment” without further criteria provided. The lack of clear criteria that would allow reproducibility is the basis for the categorization as subjective. Five studies used a mixed-methods approach, combining objective and reproducible algorithms (an objective element) which were subsequently checked by the expert researcher (a subjective element).^6,61,69,77,84^

The use of individually placed electrodes for EEG acquisition was substantially more popular than the use of EEG caps (Fig. 5B). One study used novel sensors that were for combined EEG-NIRS (near-infrared spectroscopy) acquisition.^4^ The frequency with which different EEG electrodes were used during acquisition also varied substantially (Fig. 5C). The following 8 electrodes (and number of studies) were notably the most common: Cz (36), C3 (32), C4 (32), Fz (31), T7 (30), CPz (29), T8 (29), and FCz (27).

### 3.7. Painful procedures and pain relief interventions

**Figure 6.**
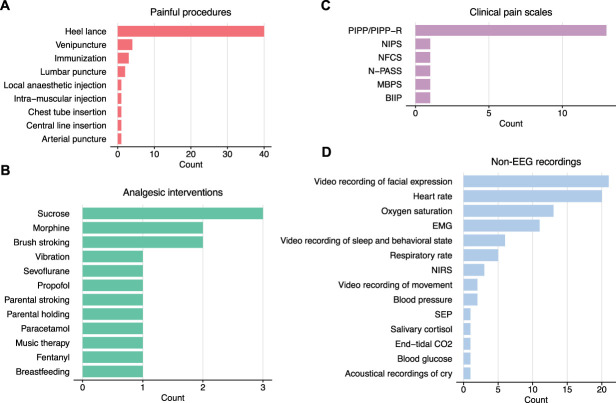
(A) Painful (ie, skin-breaking) procedures. (B) Analgesic interventions. (C) Clinical pain scales. (D) Non-EEG recordings. BIIP, Behavioral Indicators of Infant Pain; CO_2_, carbon dioxide; EMG, electromyography; MBPS, Modified Behavioral Pain Scale; NIPS, Neonatal Infant Pain Scale; NFCS, Neonatal Facial Coding System; NIRS, near-infrared spectroscopy; N-PASS, Neonatal Pain, Agitation, and Sedation Scale; PIPP(-R), Premature Infant Pain Profile (Revised); SEP, somatosensory evoked potential.

We identified 9 skin-breaking procedures used in neonatal EEG pain research (Fig. 6A), with heel lance being the most common procedure in 40/44 (91.0%) of studies. Some studies examined more than one procedure type, so the total number of procedures is larger than the number of studies.

We identified 12 interventions that were studied for their potential to provide pain relief (Fig. 6B). There were 7 nonpharmacological interventions: sucrose,^1,12,91^ music therapy,^73^ researcher-led touch-based interventions (vibration stimulation^77^ and brush stroking^21,41^), parent-led touch-based interventions (stroking^47^ and holding^54^), and breastfeeding.^12^ There were 5 pharmacological interventions: the opioid analgesics morphine and fentanyl,^42,46,69^ the nonopioid analgesic paracetamol (acetaminophen),^21^ and the anesthetics sevoflurane and propofol.^18^

### 3.8. Nonelectroencephalography recordings

Finally, we documented other non-EEG measures of pain responses that were collected along with the EEG. These included 6 neonatal clinical pain scales, with the combination of the Premature Infant Pain Profile and its revised version together being the most common clinical pain assessment tool (Fig. 6C). In addition, a host of non-EEG recordings were identified, including hemodynamic brain responses (ie, NIRS), behavioral, cardiovascular, respiratory, and hormonal responses (Fig. 6D).

## 4. Discussion

This scoping review is the first stage in an IPD meta-analysis, with the findings from this review guiding the next stages of the project.^9^ This scoping review identified 55 unique publications using EEG to assess acute pain in neonates, with 164 global researchers having published articles using EEG for this purpose. Electroencephalography has been commonly used during acute painful procedures in neonates, with studies being conducted in 11 countries since 2008. Electroencephalography has often been acquired alongside other pain-relevant recordings, which will facilitate assessments of the relationships that pain-related EEG activity may have with other pain-related signals.

We identified 13 clusters of researchers publishing in this area, with limited overlap between “islands” of authors. To consolidate and connect researchers who have investigated neonatal pain using EEG, we invited authors of the relevant literature to contribute to and coauthor this review and to facilitate future collaborations in this developing field. While EEG studies span multiple countries, most originate from Europe and the Americas, highlighting gaps in global neonatal pain research. Regional differences in neonatal care, cultural perceptions of pain, and technological access influence this disparity. Limited participation of researchers from South America, Africa, and Asia in this review limits the generalizability of the findings. This is likely due to the equipment being expensive to purchase, requires expert personnel to perform the studies and interpret the results, and is labor-intensive to maintain. Collaborative efforts must be made to ensure that future research is inclusive of diverse global populations, which will enrich the understanding and utility of EEG in neonatal pain assessment.

The EEG measures used in the studies identified in this review offer objective neurophysiological metrics that could be used to augment traditional neonatal pain scales. However, technical demands and the need for specialized personnel hinder its routine clinical use. The development of EEG-integrated monitoring systems with flexible or wireless electrodes could help bridge this gap. Developing automated systems capable of real-time EEG analysis could also help make EEG a more practical tool for routine bedside pain assessment. In addition, EEG biomarkers of neonatal pain could be used in nonroutine, well-structured, high technology contexts such as clinical trials either as a stand-alone outcome or as one item in a multidimensional pain scale assessment.^82,83^ The technical and logistical needs, as well as the acceptability of the technique, will depend on the context of use. Before an EEG metric will be broadly acceptable, either as an item to be integrated into traditional neonatal pain scales or as a stand-alone outcome, basic measurements of reliability, validity, and interpretability will need to be established for any EEG metric.^9,25^ In addition, optimal methods for EEG monitoring and electrode placement in neonates are not readily available, especially for longer-term monitoring, highlighting the need for standardized protocols.^59^

Regardless of how EEG biomarkers of acute neonatal pain are implemented, integrated, or scaled up (eg, wired or wireless individual electrodes or caps; integrated with existing pain-relevant indicators and pain scales or used as stand-alone outcomes; scaled up for routine clinical use or large-scale clinical trials), data quality issues must be considered carefully. In this review, we identified that loss of participants from analysis due to EEG data quality seems to be a significant issue for some studies. The median data loss was 3.3%, which is consistent with similar numbers reported with the use of nonpainful stimuli in this age range,^94,102^ suggesting the issue is likely to be related to the neonatal population rather than the painful procedure. Furthermore, the methods involved in assessing EEG data quality were not always clearly presented and often left undescribed, thus limiting the reproducibility of this step in the analysis process. These reproducibility and data loss issues pose a challenge to the field. Loss of data will reduce precision and may introduce selection bias (eg, neonates with more movement-related artifacts may be disproportionately excluded from analysis)^48,49^ or bias by indication (ie, certain types of neonates requiring a specific type of painful procedure), and the resulting lack of reproducibility will be a barrier to general uptake. Addressing these limitations requires a concerted effort to develop better technologies and standardized protocols. Innovations in EEG hardware (eg, wireless electrodes) could reduce movement-related artifacts, and standardized protocols with clear data quality criteria that combine objective algorithms with human verification for data quality control could improve reproducibility and reduce risk of bias. For research studies including EEG assessments, all these complexities will require careful specification of analytic methods in statistical analysis plans, which include sensitivity tests and appropriate methods to assess and handle missing data.^29^

We identified multiple distinct research groups with relatively large data sets, demonstrating the existence of sufficient EEG data recorded in neonates during acute skin-breaking procedures to conduct an IPD meta-analysis designed to assess the reliability, validity, and interpretability of a specific EEG outcome measure.^9^ Compared with standard aggregate data meta-analysis, an IPD meta-analysis can potentially provide substantial improvements to the extent and quality of data available, by including both reported and unreported data and addressing data completeness and quality issues directly with original investigators.^98^ This approach could increase sample size, statistical power, and generalizability.^78^ Collaborating with original investigators in an IPD meta-analysis study provides deeper insights and fosters a network for sharing neonatal EEG data, enabling novel research opportunities. Other research groups have already begun generously sharing their neonatal pain IPD data sets,^55^ which has created new research opportunities and findings.^96,100^ Through our IPD meta-analysis project, we plan to continue expanding and advancing the data sharing efforts to help transform the field of neonatal pain research.^9^

Pooling of data across studies will be limited by heterogeneity and inconsistencies in data collection methodologies. In this review, we identified that electrode coverage of the scalp varied considerably, with central electrodes being the most recorded. For analyses that require central electrodes, such as the specific Cz-Fz channel biomarker that we are assessing in our IPD meta-analysis, significant value will be gained from pooling these data sets. However, for research questions that require recordings from brain regions not covered by certain electrodes, or for analytic methods that require relatively high electrode numbers, use of existing data sets might be more limited. For example, microstate analysis is becoming increasingly popular in neonatal EEG analysis,^14,66,80^ using information that can be obtained from a full-scalp electrode array to detect and analyze signals otherwise inaccessible to traditional event-related potential analysis, such as signals from the subcortical loci used for pain processing. Pooling existing data sets with limited and inconsistent scalp coverage may cause more challenges to implementing microstate analysis than event-related potential analysis. However, highlighting challenges is often a necessary first step in overcoming them, and methodological progress for complex nonevent-related potential analytic approaches would likely accelerate if researchers had access to these pooled data sets.

## 5. Conclusion

The development of more accurate and reproducible pain measures for neonates is urgently needed. This scoping review is an initial component of a larger project addressing this issue by taking the necessary steps to establish a pain-related EEG biomarker to help create novel brain-based clinical pain measures. This review identified 55 studies that used EEG-based measures to help quantify changes in noxious stimulation-evoked brain activity in neonates. Identifying this substantial body of literature through a scoping review raises awareness of the value that neonatal EEG studies can bring to better understand the occurrence and treatment of neonatal pain. There is great value in bringing these data sets together as these difficult-to-collect data have tremendous value beyond the single studies for which the data were initially collected. Future collaborative efforts will further develop the data acquisition and analytic skills that are needed to further progress this field. This work forms the groundwork for a future IPD meta-analysis where we can bring this community together to share and store data using the infrastructure within the Critical-Path Institute's Rare Disease Cures Accelerator-Data and Analytics Platform.^7^ This is a valuable initiative funded by the US Food and Drug Administration that provides a centralized and standardized data analytics infrastructure to ensure safe and secure data storage that meets FAIR (Findability, Accessibility, Interoperability, and Reusability) data principles.^52,108^ By bringing this community together, great advances are possible in the use of brain-derived approaches to better understand how afferent noxious input is processed by the immature neonatal brain and ultimately how to modify these signals to improve the experiences of newborn babies.

## Disclosures

B.G. has stock ownership in Autonomous Healthcare Inc. and serves as its CEO. All other authors have no conflicts of interest relevant to this article to disclose. The opinions expressed in this article are those of the authors and should not be interpreted as the position of the US Food and Drug Administration.

## Supplemental digital content

Supplemental digital content associated with this article can be found online at http://links.lww.com/PR9/A400.

## Supplementary Material

SUPPLEMENTARY MATERIAL

## References

[1] Abdelsami Awad H Hassanein S Mohamed Abdou R Taher Bassiouny L. Analysis of pain effect on EEG recordings and oral sucrose suckling effect on pain reduction in neonates. QJM Int J Med 2018;111:hcy200.158.

[2] Anand KJS. Assessment of neonatal pain. 2019. Available at: https://www.uptodate.com/contents/assessment-of-neonatal-pain/. Accessed January 4, 2022.

[3] Anand KJS. Prevention and treatment of neonatal pain. Waltham, MA: Wolters Kluwer Publisher; 2021.

[4] Askari S Bastany Z Holsti L Dumont GD. Lighting up babies’ brains: development of a combined NIRS/EEG system for infants. Biophotonics in exercise science, sports medicine, health monitoring technologies, and wearables II. Bellingham, WA, USA: SPIE; 2021:Vol. 11638:80–5.

[5] Aspbury M. Developing neuroimaging methods for clinical translation and better understanding neonatal brain development. University of Oxford; 2023. Available at: http://purl.org/dc/dcmitype/Text; https://ora.ox.ac.uk/objects/uuid:3ff54503-a5cc-48bd-8518-6e018efa8905. Accessed February 23, 2024.

[6] Aspbury M Mansfield RC Baxter L Bhatt A Cobo MM Fitzgibbon SP Hartley C Hauck A Marchant S Monk V Pillay K Poorun R van der Vaart M Slater R. Establishing a standardised approach for the measurement of neonatal noxious-evoked brain activity in response to an acute somatic nociceptive heel lance stimulus. Cortex 2024;179:215–34. 39197410 10.1016/j.cortex.2024.05.023 PMC11913738

[7] Barrett JS Betourne A Walls RL Lasater K Russell S Borens A Rohatagi S Roddy W. The future of rare disease drug development: the rare disease cures accelerator data analytics platform (RDCA-DAP). J Pharmacokinet Pharmacodyn 2023;50:507–19. 37131052 10.1007/s10928-023-09859-7 PMC10673974

[8] Baxter L Hauck AGV Bhatt A Cobo MM Hartley C Marchant S Poorun R van der Vaart M Slater R. Statistical analysis plan for the Petal trial: the effects of parental touch on relieving acute procedural pain in neonates. Wellcome Open Res 2024;8:402. 40110538 10.12688/wellcomeopenres.19819.3 PMC11920693

[9] Baxter L van der Vaart M Cobo MM Gunawan PY Allegaert K Davis JM Turner M Ward RM Darsey E Sheppard JP Bhatt A van den Anker J Massaro AN Walls RL Song LS Singh K Apele-Freimane D Arimistu T Barry C Discenza D Giola O Hovinga C Jackson Y Matthews D Mehta V Reginato Cascamo K Vivas N Slater R, INC Pain Working Group. Is noxious stimulus-evoked electroencephalography response a reliable, valid, and interpretable outcome measure to assess analgesic efficacy in neonates? A systematic review and individual participant data (IPD) meta-analysis protocol. Syst Rev 2025;14:152. 40713869 10.1186/s13643-025-02890-4 PMC12296588

[10] Benoit B. The influence of breastfeeding on cortical activity during procedures (iCAP). ClinicalTrials.gov, 2017. Available at: https://clinicaltrials.gov/study/NCT03272594. Accessed August 18, 2024.

[11] Benoit B Martin-Misener R Newman A Latimer M Campbell-Yeo M. Neurophysiological assessment of acute pain in infants: a scoping review of research methods. Acta Paediatr 2017;106:1053–66. 28326623 10.1111/apa.13839

[12] Benoit B Newman A Martin-Misener R Latimer M Campbell-Yeo M. The influence of breastfeeding on cortical and bio-behavioural indicators of procedural pain in newborns: findings of a randomized controlled trial. Early Hum Develop 2021;154:105308. 10.1016/j.earlhumdev.2021.105308 33513546

[13] Bucsea O. Examining the relationships between neonatal pain-related facial actions and cortical activity. Toronto: York University, 2020. Available at: http://hdl.handle.net/10315/37946. Accessed August 18, 2024.

[14] Bucsea O Rupawala M Shiff I Wang X Meek J Fitzgerald M Fabrizi L Pillai Riddell R Jones L. Clinical thresholds in pain-related facial activity linked to differences in cortical network activation in neonates. PAIN 2023;164:1039–50. 36633530 10.1097/j.pain.0000000000002798 PMC10108588

[15] Bueno M Eriksson M Stevens BJ. Neonatal and infant pain assessment. In: Stevens BJ Hathway G Zempsky WT, editors. Oxford Textbook of Pediatric Pain. Oxford Textbook. Oxford, New York: Oxford University Press, 2021. p. 375–90.

[16] Campbell-Yeo M. The influence of skin-to-skin contact on cortical activity during painful procedures on preterm infants in the NICU (iCAPmini). ClinicalTrials.gov, 2018. Available at: https://clinicaltrials.gov/study/NCT03745963. 10.1186/s13063-022-06424-4 PMC9208173 35725632

[17] Campbell-Yeo M Benoit B Newman A Johnston C Bardouille T Stevens B Jiang A. The influence of skin-to-skin contact on cortical activity during painful procedures in preterm infants in the neonatal intensive care unit (iCAP mini): study protocol for a randomized control trial. Trials 2022;23:512. 35725632 10.1186/s13063-022-06424-4 PMC9208173

[18] Castillo P Vanhatalo S Lundblad M Blennow M Lonnqvist PA. EEG response to a high volume (1.5 mL/kg) caudal block in infants less than 3 months. Reg Anesth Pain Med 2023;49:163–7. 10.1136/rapm-2023-104452 37364921

[19] Cobo MM. Developing translational tools for measuring pain in neonates. University of Oxford, 2022. Available at: http://purl.org/dc/dcmitype/Text; https://ora.ox.ac.uk/objects/uuid:c3d39756-706c-4bad-816f-88fdadf92c3b. Accessed February 23, 2024.

[20] Cobo MM Green G Andritsou F Baxter L Evans Fry R Grabbe A Gursul D Hoskin A Mellado GS van der Vaart M Adams E Bhatt A Denk F Hartley C Slater R. Early life inflammation is associated with spinal cord excitability and nociceptive sensitivity in human infants. Nat Commun 2022;13:3943. 35803920 10.1038/s41467-022-31505-y PMC9270448

[21] Cobo MM Hartley C Gursul D Andritsou F van der Vaart M Schmidt Mellado G Baxter L Duff EP Buckle M Evans Fry R Green G Hoskin A Rogers R Adams E Moultrie F Slater R. Quantifying noxious-evoked baseline sensitivity in neonates to optimise analgesic trials. eLife 2021;10:e65266. 33847561 10.7554/eLife.65266 PMC8087440

[22] Cobo MM Moultrie F Hauck AGV Crankshaw D Monk V Hartley C Evans Fry R Robinson S van der Vaart M Baxter L Adams E Poorun R Bhatt A Slater R. Multicentre, randomised controlled trial to investigate the effects of parental touch on relieving acute procedural pain in neonates (Petal). BMJ Open 2022;12:e061841. 10.1136/bmjopen-2022-061841 PMC9301810 36250332

[23] Cornelissen L. Innovative approaches to assessment of pain control and sedation in the NICU. ClinicalTrials.gov, 2016. Available at: https://clinicaltrials.gov/study/NCT03057782. Accessed August 18, 2024.

[24] Coviello C Lori S Bertini G Montano S Gabbanini S Bastianelli M Cossu C Cavaliere S Lunardi C Dani C. Evaluation of the relationship between pain exposure and somatosensory evoked potentials in preterm infants: a prospective cohort study. Children 2024;11:676. 38929255 10.3390/children11060676 PMC11201689

[25] De Vet HCW Terwee CB Mokkink LB Knol DL. Measurement in medicine: A practical guide. Cambridge: Cambridge University Press, 2011. doi: 10.1017/CBO9780511996214

[26] De Vet HCW Terwee CB Mokkink LB Knol DL. Validity. Measurement in medicine: A practical guide. New York, NY, USA: Cambridge University Press; 2011:150–201.

[27] Dempsey G. The effect of a musical intervention on stress response to venepuncture. ClinicalTrials.gov, 2017. Available at: https://clinicaltrials.gov/study/NCT03028844. Accessed August 18, 2024.

[28] van Eck NJ Waltman L. Software survey: VOSviewer, a computer program for bibliometric mapping. Scientometrics 2010;84:523–38. 20585380 10.1007/s11192-009-0146-3 PMC2883932

[29] EMA. Guideline on missing data in confirmatory clinical trials, 2010. Available at: https://www.ema.europa.eu/en/documents/scientific-guideline/guideline-missing-data-confirmatory-clinical-trials_en.pdf. Accessed January 26, 2025.

[30] EMA. ICH E11(R1) step 5 guideline on clinical investigation of medicinal products in the pediatric population. European Medicines Agency, 2017. Available at: https://www.ema.europa.eu/en/ich-e11r1-step-5-guideline-clinical-investigation-medicinal-products-pediatric-population. Accessed August 1, 2022.

[31] EMA. Investigation of medicinal products in the term and preterm neonate. European Medicines Agency, 2009. Available at: https://www.ema.europa.eu/en/investigation-medicinal-products-term-preterm-neonate. Accessed August 1, 2022.

[32] Fabrizi L Slater R Worley A Meek J Boyd S Olhede S Fitzgerald M. A shift in sensory processing that enables the developing human brain to discriminate touch from pain. Curr Biol 2011;21:1552–8. 21906948 10.1016/j.cub.2011.08.010 PMC3191265

[33] Fabrizi L Slater R Worley A Meek J Olhede S Boyd S Fitzgerald M. P14-24 development of a cortical electrophysiological response to noxious stimulation in human infants. Clin Neurophysiol 2010;121:S190.

[34] Fabrizi L Verriotis M Williams G Lee A Meek J Olhede S Fitzgerald M. Encoding of mechanical nociception differs in the adult and infant brain. Sci Rep 2016;6:28642. 27345331 10.1038/srep28642 PMC4921818

[35] Fabrizi L Worley A Patten D Holdridge S Cornelissen L Meek J Boyd S Slater R. Electrophysiological measurements and analysis of nociception in human infants. J Vis Exp. 2011;(58):e3118. doi:10.3791/3118. PMC3369648 22214879

[36] FDA. FDA-M CERSI: Analgesic clinical trial designs, extrapolation, and endpoints in patients from birth to less than two years of age public workshop. FDA, 2021. Available at: https://www.fda.gov/drugs/news-events-human-drugs/fda-m-cersi-analgesic-clinical-trial-designs-extrapolation-and-endpoints-patients-birth-less-two. Accessed August 19, 2024.

[37] Fellman V. NeoFent-I study; fentanyl treatment in newborn infants; a pharmacokinetic, pharmacodynamic and pharmacogenetic study. ClinicalTrials.gov, 2012. Available at: https://clinicaltrials.gov/study/NCT03897452. Accessed August 18, 2024.

[38] Goksan S. Imaging nociceptive brain activity in the newborn infant. University of Oxford, 2016. Available at: http://purl.org/dc/dcmitype/Text; https://ora.ox.ac.uk/objects/uuid:ea4d49fc-cf7e-4775-bb82-ddb3385cc2d9. Accessed August 18, 2024.

[39] Green G. Measuring pain in the newborn infant. University of Oxford, 2018. Available at: http://purl.org/dc/dcmitype/Text; https://ora.ox.ac.uk/objects/uuid:5647e78c-48fb-4b1d-a54f-146803bd7037. Accessed August 18, 2024.

[40] Green G Hartley C Hoskin A Duff E Shriver A Wilkinson D Adams E Rogers R Moultrie F Slater R. Behavioural discrimination of noxious stimuli in infants is dependent on brain maturation. PAIN 2019;160:493–500. 30422872 10.1097/j.pain.0000000000001425 PMC6343955

[41] Gursul D Goksan S Hartley C Mellado GS Moultrie F Hoskin A Adams E Hathway G Walker S McGlone F Slater R. Stroking modulates noxious-evoked brain activity in human infants. Curr Biol 2018;28:R1380–1. 30562526 10.1016/j.cub.2018.11.014 PMC6303187

[42] Hartley C Baxter L Moultrie F Purdy R Bhatt A Rogers R Patel C Adams E Slater R. Predicting severity of adverse cardiorespiratory effects of morphine in premature infants: a post hoc analysis of Procedural Pain in Premature Infants trial data. Br J Anaesth 2021;126:e133–5. 33309053 10.1016/j.bja.2020.10.034 PMC8767644

[43] Hartley C Duff EP Green G Mellado GS Worley A Rogers R Slater R. Nociceptive brain activity as a measure of analgesic efficacy in infants. Sci Translat Med 2017;9:eaah6122. 10.1126/scitranslmed.aah6122 PMC5884430 28469039

[44] Hartley C Goksan S Poorun R Brotherhood K Mellado GS Moultrie F Rogers R Adams E Slater R. The relationship between nociceptive brain activity, spinal reflex withdrawal and behaviour in newborn infants. Sci Rep 2015;5:12519. 26228435 10.1038/srep12519 PMC4521152

[45] Hartley C Moultrie F Gursul D Hoskin A Adams E Rogers R Slater R. Changing balance of spinal cord excitability and nociceptive brain activity in early human development. Curr Biol 2016;26:1998–2002. 27374336 10.1016/j.cub.2016.05.054 PMC4985558

[46] Hartley C Moultrie F Hoskin A Green G Monk V Bell JL King AR Buckle M van der Vaart M Gursul D Goksan S Juszczak E Norman JE Rogers R Patel C Adams E Slater R. Analgesic efficacy and safety of morphine in the Procedural Pain in Premature Infants (Poppi) study: randomised placebo-controlled trial. Lancet 2018;392:2595–605. 30509743 10.1016/S0140-6736(18)31813-0 PMC6294828

[47] Hauck AGV van der Vaart M Adams E Baxter L Bhatt A Crankshaw D Dhami A Evans Fry R Freire MBO Hartley C Mansfield RC Marchant S Monk V Moultrie F Peck M Robinson S Yong J Poorun R Cobo MM Slater R. Effect of parental touch on relieving acute procedural pain in neonates and parental anxiety (Petal): a multicentre, randomised controlled trial in the UK. Lancet Child Adolesc Health 2024;8:259–69. 38373429 10.1016/S2352-4642(23)00340-1 PMC7618171

[48] Higgins J Savović J Page M Elbers R Sterne J. Chapter 8: assessing risk of bias in a randomized trial. In: Higgins J Thomas J Chandler J Cumpston M Li T Page M Welch V, editors. Cochrane Handbook for Systematic Reviews of Interventions. Cochrane, 2023. Available at: www.training.cochrane.org/handbook. Accessed November 11, 2023.

[49] Higgins JPT Savović J Page MJ Sterne JA. Revised Cochrane risk-of-bias tool for randomized trials (RoB 2), 2019. Available at: https://drive.google.com/file/d/19R9savfPdCHC8XLz2iiMvL_71lPJERWK/view?usp=drive_open&usp=embed_facebook. Accessed August 19, 2024.

[50] Hohsoh N Iwata O Suzuki T Hanai C Huang M Yokoyama K. Quantification electroencephalography response to procedural pain during heel puncture in preterm infants. Physiol Meas 2025;46:065004. 10.1088/1361-6579/addfa9 40456262

[51] IASP. IASP terminology. IASP, 2024. Available at: https://www.iasp-pain.org/resources/terminology/. Accessed June 13, 2020.

[52] Jacobsen A de Miranda Azevedo R Juty N Batista D Coles S Cornet R Courtot M Crosas M Dumontier M Evelo CT Goble C Guizzardi G Hansen KK Hasnain A Hettne K Heringa J Hooft RWW Imming M Jeffery KG Kaliyaperumal R Kersloot MG Kirkpatrick CR Kuhn T Labastida I Magagna B McQuilton P Meyers N Montesanti A van Reisen M Rocca-Serra P Pergl R Sansone S-A da Silva Santos LOB Schneider J Strawn G Thompson M Waagmeester A Weigel T Wilkinson MD Willighagen EL Wittenburg P Roos M Mons B Schultes E. FAIR principles: interpretations and implementation considerations. Data Intelligence 2020;2:10–29.

[53] Jones L Fabrizi L Laudiano-Dray M Whitehead K Meek J Verriotis M Fitzgerald M. Nociceptive cortical activity is dissociated from nociceptive behavior in newborn human infants under stress. Curr Biol 2017;27:3846–51.e3. 29199079 10.1016/j.cub.2017.10.063 PMC5742634

[54] Jones L Laudiano-Dray MP Whitehead K Meek J Fitzgerald M Fabrizi L Pillai Riddell R. The impact of parental contact upon cortical noxious-related activity in human neonates. Eur J Pain 2021;25:149–59. 32965725 10.1002/ejp.1656 PMC8436758

[55] Jones L Laudiano-Dray MP Whitehead K Verriotis M Meek J Fitzgerald M Fabrizi L. EEG, behavioural and physiological recordings following a painful procedure in human neonates. Sci Data 2018;5:180248. 30422128 10.1038/sdata.2018.248 PMC6233256

[56] Jones L Verriotis M Laudiano-Dray M Whitehead K Fabrizi L Meek J Fitzgerald M. Behavioural and cortical pain responses in human infants are dissociable by their relationship to physiological stress. British Neuroscience Association Festival of Neuroscience, BNA 2017 Birmingham, United Kingdom; 2017;1:144.

[57] Kuhn P. Multimodal approach to the ontogenesis of nociception in very preterm and term infants (NOCI-Prem). ClinicalTrials.gov, 2019. Available at: https://clinicaltrials.gov/study/NCT05404594. Accessed August 18, 2024.

[58] Lee G Stevens B. Neonatal and infant pain assessment. Oxford Textbook of Paediatric Pain. Oxford, UK: Oxford University Press; 2013:353–69.

[59] Lloyd R Goulding R Filan P Boylan G. Overcoming the practical challenges of electroencephalography for very preterm infants in the neonatal intensive care unit. Acta Paediatr 2015;104:152–7. 25495482 10.1111/apa.12869 PMC5024034

[60] Maimon N Grunau RE Cepeda IL Friger M Selnovik L Gilat S Shany E. Electroencephalographic activity in response to procedural pain in preterm infants born at 28 and 33 weeks gestational age. Clin J Pain 2013;29:1044–9. 23446071 10.1097/AJP.0b013e318284e525

[61] Maitre NL Stark AR McCoy Menser CC Chorna OD France DJ Key AF Wilkens K Moore-Clingenpeel M Wilkes DM Bruehl S. Cry presence and amplitude do not reflect cortical processing of painful stimuli in newborns with distinct responses to touch or cold. Arch Dis Child Fetal Neonatal Ed 2017;102:F428–33. 28500064 10.1136/archdischild-2016-312279 PMC5651180

[62] Marchant S van der Vaart M Pillay K Baxter L Bhatt A Fitzgibbon S Hartley C Slater R. A machine learning artefact detection method for single-channel infant event-related potential studies. J Neural Eng 2024;21:046021. 10.1088/1741-2552/ad5c04 PMC11250100 38925111

[63] McGowan J Sampson M Salzwedel DM Cogo E Foerster V Lefebvre C. PRESS peer review of electronic search strategies: 2015 guideline statement. J Clin Epidemiol 2016;75:40–6. 27005575 10.1016/j.jclinepi.2016.01.021

[64] Meek J. A clinical study investigating sucrose as a pain reliever in infants. London, UK: ISRCTN Registry; 2009.

[65] Menser CCM Bruehl S France D Wilkes D Maitre N. Assessment of neonatal pain: correlations between cortical somatosensory processing and cry acoustics. Anesth Analg 2016;122:S245.

[66] Michel CM Koenig T. EEG microstates as a tool for studying the temporal dynamics of whole-brain neuronal networks: a review. Neuroimage 2018;180:577–93. 29196270 10.1016/j.neuroimage.2017.11.062

[67] Moher D Shamseer L Clarke M Ghersi D Liberati A Petticrew M Shekelle P Stewart LA, PRISMA-P Group. Preferred reporting items for systematic review and meta-analysis protocols (PRISMA-P) 2015 statement. Syst Rev 2015;4:1. 25554246 10.1186/2046-4053-4-1 PMC4320440

[68] Monk V Moultrie F Hartley C Hoskin A Green G Bell JL Stokes C Juszczak E Norman J Rogers R Patel C Adams E Slater R. Oral morphine analgesia for preventing pain during invasive procedures in non-ventilated premature infants in hospital: the Poppi RCT. Efficacy Mechanism Eval 2019;6:1–98. 31483590

[69] Nilsson S Tokariev A Vehviläinen T Fellman V Vanhatalo S Norman E. Depression of cortical neuronal activity after a low-dose fentanyl in preterm infants. Acta Paediatr 2025;114:109–15. 39258825 10.1111/apa.17411 PMC11627449

[70] Norman E. Clonidine for analgesia to preterm infants during neonatal intensive care. ClinicalTrials.gov, 2018. Available at: https://clinicaltrials.gov/study/NCT04928651. Accessed August 18, 2024.

[71] Norman E. Fentanyl and clonidine for analgesia during hypothermia in term asphyxiated infants (SANNI 1). ClinicalTrials.gov, 2017. Available at: https://clinicaltrials.gov/study/NCT03177980. Accessed August 18, 2024.

[72] Norman E Rosén I Vanhatalo S Stjernqvist K Ökland O Fellman V Hellström-Westas L. Electroencephalographic response to procedural pain in healthy term newborn infants. Pediatr Res 2008;64:429–34. 18594483 10.1203/PDR.0b013e3181825487

[73] Pavel AM Dhais FA Howard C O'Toole JM Pavlidis E Finn D Livingstone V Powell A Dempsey EM Boylan GB. GP252 the effect of music therapy on the electroencephalogram (EEG) and heart rate variability (HRV) of premature infants during routine painful procedures. Arch Dis Child 2019;104:A135.

[74] Pillai Riddell R. Rebooting infant pain assessment: Using machine learning to exponentially improve neonatal intensive care unit practice (BabyAI). ClinicalTrials.gov, 2020. Available at: https://clinicaltrials.gov/study/NCT05579496. Accessed August 18, 2024.

[75] Raja SN Carr DB Cohen M Finnerup NB Flor H Gibson S Keefe FJ Mogil JS Ringkamp M Sluka KA Song X-J Stevens B Sullivan MD Tutelman PR Ushida T Vader K. The revised International Association for the Study of Pain definition of pain: concepts, challenges, and compromises. PAIN 2020;161:1976–82. 32694387 10.1097/j.pain.0000000000001939 PMC7680716

[76] Relland LM. Effect of a vibratory stimulus on mitigating nociception-specific responses to skin puncture in neonates. ClinicalTrials.gov, 2018. Available at: https://clinicaltrials.gov/study/NCT04050384. Accessed August 18, 2024.

[77] Relland LM Kjeldsen CP Jeanvoine A Emery L Adderley K Srinivas R McLoughlin M Maitre NL. Vibration-based mitigation of noxious-evoked responses to skin puncture in neonates and infants: a randomised controlled trial. Arch Dis Child Fetal Neonatal Ed 2024;109:622–7. 38479794 10.1136/archdischild-2023-326588

[78] Riley RD Tierney JF Stewart LA. Individual participant data meta-analysis: A handbook for healthcare research. Newark, United Kingdom. John Wiley & Sons, Incorporated, 2021. Available at: http://ebookcentral.proquest.com/lib/oxford/detail.action?docID=6629933. Accessed August 19, 2024.

[79] Roué J-M Avnit A Gholami B Haddad WM Anand KJS. Objective detection of newborn infant acute procedural pain using EEG and machine learning algorithms. Paediatric Neonatal Pain 2025;7:e70001. 40066435 10.1002/pne2.70001 PMC11891568

[80] Rupawala M Bucsea O Laudiano-Dray MP Whitehead K Meek J Fitzgerald M Olhede S Jones L Fabrizi L. A developmental shift in habituation to pain in human neonates. Curr Biol 2023;33:1397–406.e5. 36931271 10.1016/j.cub.2023.02.071

[81] Rupawala M Bucsea O Laudiano-Dray MP Whitehead K Meek J Fitzgerald M Olhede S Jones L Fabrizi L. Developmental switch in prediction and adaptation to pain in human neonates. bioRxiv preprint. 2022. doi:10.1101/2022.04.05.486988. 36931271

[82] Sankoh AJ Li H D'Agostino RB. Composite and multicomponent end points in clinical trials. Stat Med 2017;36:4437–40. 28675919 10.1002/sim.7386

[83] Sankoh AJ Li H D'Agostino RB. Use of composite endpoints in clinical trials. Stat Med 2014;33:4709–14. 24833282 10.1002/sim.6205

[84] Schmidt Mellado G Pillay K Adams E Alarcon A Andritsou F Cobo MM Evans Fry R Fitzgibbon S Moultrie F Baxter L Slater R. The impact of premature extrauterine exposure on infants' stimulus-evoked brain activity across multiple sensory systems. Neuroimage Clin 2022;33:102914. 34915328 10.1016/j.nicl.2021.102914 PMC8683775

[85] Shafiee R Daliri MR. Decoding of pain during heel lancing in human neonates with EEG signal and machine learning approach. Sci Rep 2024;14:31244. 39732802 10.1038/s41598-024-82631-0 PMC11682341

[86] Shiroshita Y Kirimoto H Ozawa M Watanabe T Uematsu H Yunoki K Sobue I. Can event-related potentials evoked by heel lance assess pain processing in neonates? A systematic review. Children (Basel) 2021;8:58. 33498331 10.3390/children8020058 PMC7909417

[87] Slater R. A blinded randomised placebo-controlled trial investigating the efficacy of morphine analgesia for procedural pain in infants. EU Clinical Trials Register, 2015. Available at: https://www.clinicaltrialsregister.eu/ctr-search/search?query=eudract_number:2014-003237-25. Accessed August 18, 2024. 10.12688/wellcomeopenres.10005.2 PMC5218543 28066825

[88] Slater R. FDA public workshop: Brain-derived approaches to assess neonatal & infant pain, 2021. Available at: https://www.fda.gov/media/153237/download. Accessed July 18, 2023.

[89] Slater R. Parental touch trial (Petal). ClinicalTrials.gov, 2021. Available at: https://clinicaltrials.gov/study/NCT04901611. Accessed August 18, 2024.

[90] Slater R. Using parental touch to relieve pain in newborn infants. London, UK: ISRCTN Registry; 2021.

[91] Slater R Cornelissen L Fabrizi L Patten D Yoxen J Worley A Boyd S Meek J Fitzgerald M. Oral sucrose as an analgesic drug for procedural pain in newborn infants: a randomised controlled trial. Lancet 2010;376:1225–32. 20817247 10.1016/S0140-6736(10)61303-7 PMC2958259

[92] Slater R Fabrizi L Worley A Meek J Boyd S Fitzgerald M. Premature infants display increased noxious-evoked neuronal activity in the brain compared to healthy age-matched term-born infants. Neuroimage 2010;52:583–9. 20438855 10.1016/j.neuroimage.2010.04.253

[93] Slater R Worley A Fabrizi L Roberts S Meek J Boyd S Fitzgerald M. Evoked potentials generated by noxious stimulation in the human infant brain. Eur J Pain 2010;14:321–6. 19481484 10.1016/j.ejpain.2009.05.005

[94] Stets M Stahl D Reid VM. A meta-analysis investigating factors underlying attrition rates in infant ERP studies. Develop Neuropsychol 2012;37:226–52. 10.1080/87565641.2012.654867 22545660

[95] Talebi S Frounchi J Mozaffari Tazehkand B. A novel channel selection approach for human neonate's pain EEG data analysis. SIViP 2025;19:364.

[96] Talebi S Frounchi J Tazehkand BM. A novel channel selection approach for human neonate’s pain EEG data analysis. Durham, NC: Research Square; 2022.

[97] Thomas J Graziosi S Brunton J Ghouze Z O'Driscoll P Bond M Koryakina A. EPPI-Reviewer: Advanced software for systematic reviews, maps and evidence synthesis, 2022. Available at: http://eppi.ioe.ac.uk/cms/Default.aspx?tabid=2914. Accessed May 25, 2022.

[98] Tierney JF Riley RD Tudur Smith C Clarke M Stewart LA. Rationale for embarking on an IPD meta-analysis project. Individual participant data meta-analysis: A handbook for healthcare research. Newark, United Kingdom: John Wiley & Sons, Incorporated, 2021. p. 9–19. Available at: http://ebookcentral.proquest.com/lib/oxford/detail.action?docID=6629933. Accessed August 19, 2024.

[99] van der Vaart M Duff E Raafat N Rogers R Hartley C Slater R. Multimodal pain assessment improves discrimination between noxious and non-noxious stimuli in infants. Paediatric Neonatal Pain 2019;1:21–30. 35546868 10.1002/pne2.12007 PMC8974881

[100] van der Vaart M Hartley C Baxter L Mellado GS Andritsou F Cobo MM Fry RE Adams E Fitzgibbon S Slater R. Premature infants display discriminable behavioral, physiological, and brain responses to noxious and nonnoxious stimuli. Cereb Cortex 2022;32:3799–815. 34958675 10.1093/cercor/bhab449 PMC9433423

[101] van der Vaart ML. Multimodal assessment of neonatal pain. University of Oxford, 2022. Available at: http://purl.org/dc/dcmitype/Text; https://ora.ox.ac.uk/objects/uuid:c18e5c7d-e36e-440b-851f-c8cf1393a95c. Accessed February 23, 2024.

[102] van der Velde B Junge C. Limiting data loss in infant EEG: putting hunches to the test. Develop Cogn Neurosci 2020;45:100809. 10.1016/j.dcn.2020.100809 PMC7358181 32658760

[103] Verriotis M Fabrizi L Lee A Cooper RJ Fitzgerald M Meek J. Mapping cortical responses to somatosensory stimuli in human infants with simultaneous near-infrared spectroscopy and event-related potential recording. eNeuro 2016;3:ENEURO.0026-16.2016. 10.1523/ENEURO.0026-16.2016 PMC4867026 27200413

[104] Verriotis M Fabrizi L Lee A Ledwidge S Meek J Fitzgerald M. Cortical activity evoked by inoculation needle prick in infants up to one-year old. PAIN 2015;156:222–30. 25599443 10.1097/01.j.pain.0000460302.56325.0c PMC4309489

[105] Verriotis M Jones L Whitehead K Laudiano-Dray M Panayotidis I Patel H Meek J Fabrizi L Fitzgerald M. The distribution of pain activity across the human neonatal brain is sex dependent. Neuroimage 2018;178:69–77. 29763673 10.1016/j.neuroimage.2018.05.030 PMC6062722

[106] Walco GA Kopecky EA Weisman SJ Stinson J Stevens B Desjardins PJ Berde CB Krane EJ Anand KJS Yaster M Dampier CD Dworkin RH Gilron I Lynn AM Maxwell LG Raja S Schachtel B Turk DC. Clinical trial designs and models for analgesic medications for acute pain in neonates, infants, toddlers, children, and adolescents: ACTTION recommendations. PAIN 2018;159:193–205. 29140927 10.1097/j.pain.0000000000001104 PMC5949239

[107] Wang S. Effects of music based intervention (MBI) on pain response and neurodevelopment in preterm infants. ClinicalTrials.gov, 2020. Available at: https://clinicaltrials.gov/study/NCT04286269. Accessed August 18, 2024.

[108] Wilkinson MD Dumontier M Aalbersberg IJJ Appleton G Axton M Baak A Blomberg N Boiten J-W da Silva Santos LB Bourne PE Bouwman J Brookes AJ Clark T Crosas M Dillo I Dumon O Edmunds S Evelo CT Finkers R Gonzalez-Beltran A Gray AJG Groth P Goble C Grethe JS Heringa J 't Hoen PAC Hooft R Kuhn T Kok R Kok J Lusher SJ Martone ME Mons A Packer AL Persson B Rocca-Serra P Roos M van Schaik R Sansone S-A Schultes E Sengstag T Slater T Strawn G Swertz MA Thompson M van der Lei J van Mulligen E Velterop J Waagmeester A Wittenburg P Wolstencroft K Zhao J Mons B. The FAIR guiding principles for scientific data management and stewardship. Sci Data 2016;3:160018. 26978244 10.1038/sdata.2016.18 PMC4792175

[109] Worley A Fabrizi L Boyd S Slater R. Multi-modal pain measurements in infants. J Neurosci Methods 2012;205:252–7. 22285660 10.1016/j.jneumeth.2012.01.009 PMC3465552

[110] Worley A Pillay K Cobo MM Mellado GS van der Vaart M Bhatt A Hartley C. The PiNe box: development and validation of an electronic device to time-lock multimodal responses to sensory stimuli in hospitalised infants. PLoS One 2023;18:e0288488. 37440586 10.1371/journal.pone.0288488 PMC10343045

