# Supplementary material for: Electroencephalography during acute painful procedures in neonates: a scoping review
Source: Pain Rep. 2026 Apr 9;11(3):e1437. doi: 10.1097/PR9.0000000000001437 (PMC13068459; doi:10.1097/PR9.0000000000001437)
Supplement: SUPPLEMENTARY MATERIAL [file painreports-11-e1437-s001.pdf]

Supplementary Information

Electroencephalography During Acute Painful Procedures in Neonates: A Scoping Review

Gunawan et al.

Table of Contents

Supplementary Methods *3*

Eligibility criteria 4

Search strategies 5

Data dictionary 10

Supplementary Results *12*

Non-primary publications excluded from synthesis 13

Primary publications included in synthesis 15

Authors to contact 20

Data extraction 26

Researcher co-authorship network clusters 123

Supplementary Methods

Eligibility criteria

Supplementary table 1: Eligibility criteria.

Inclusion criteria	
**Population**	Human neonates
**Intervention**	Acute somatic nociceptive skin-breaking procedure
**Comparator**	n/a
**Outcome**	EEG measures of intervention-evoked brain activity
**Study design**	Primary empirical studies with any study design e.g. interventional, observational, controlled, uncontrolled.
**Report characteristics**	Primary empirical research. All report types e.g. peer-reviewed publications, grey literature. Any year of dissemination. Any publication language.
**Exclusion criteria**	
**Population**	Human non-neonates; non-humans
**Intervention**	Non-acute noxious conditions, non-somatic noxious conditions, non-skin-breaking procedures
**Comparator**	n/a
**Outcome**	Studies that do not include EEG measures of brain activity evoked by acute somatic nociceptive skin-breaking procedures
**Study design**	None
**Report characteristics**	Secondary literature e.g. reviews, book chapters. Non-empirical research e.g. commentaries, opinions, perspectives.

Search strategies

MEDLINE (Ovid)

1 infant/ or infant, newborn/ or infant, low birth weight/ or infant, small for gestational age/ or infant, very low birth weight/ or infant, extremely low birth weight/ or infant, postmature/ or infant, premature/ or infant, extremely premature/ or birth weight/ or (perinatal* or neonatal* or neo-natal* or postnatal* or post-natal* or baby or babies or neonate* or neo-nate* or newborn* or new-born* or infant*).ti,ab.

2 pain/ or acute pain/ or nociceptive pain/ or pain, postoperative/ or pain, procedural/ or pain perception/ or nociception/ or hyperalgesia/ or pain measurement/ or pain management/ or pain threshold/ or nociceptors/ or analgesia/ or (pain* or nocicept* or noxious* or allodynia* or allo-dynia* or hyperalges* or hyper-alges* or hypoalges* or hypo-alges* or analges*).ti,ab.

3 electroencephalography/ or evoked potentials/ or laser-evoked potentials/ or cortical synchronization/ or electroencephalography phase synchronization/ or brain waves/ or alpha rhythm/ or beta rhythm/ or delta rhythm/ or gamma rhythm/ or theta rhythm/ or cortical excitability/ or (eeg* or electroencephalogra* or electro-encephalogra* or erp or event-related potential* or event related potential* or evoked response* or brain activit* or brain function* or cortical activit* or cortical function*).ti,ab.

4 1 and 2 and 3

Embase (Ovid)

1 infant/ or baby/ or high risk infant/ or hospitalized infant/ or newborn/ or low birth weight/ or small for date infant/ or very low birth weight/ or extremely low birth weight/ or postmaturity/ or prematurity/ or birth weight/ or high birth weight/ or (perinatal* or neonatal* or neo-natal* or postnatal* or post-natal* or baby or babies or neonate* or neo-nate* or newborn* or new-born* or infant*).ti,ab.

2 pain/ or allodynia/ or mechanical allodynia/ or tactile allodynia/ or thermal allodynia/ or cold allodynia/ or heat allodynia/ or experimental pain/ or hyperalgesia/ or mechanical hyperalgesia/ or opioid induced hyperalgesia/ or thermal hyperalgesia/ or cold hyperalgesia/ or heat hyperalgesia/ or hypoalgesia/ or thermal hypoalgesia/ or inflammatory pain/ or injection pain/ or injection site pain/ or nociceptive pain/ or postoperative pain/ or procedural pain/ or pain assessment/ or behavioral pain scale/ or nociception/ or gate control theory/ or nociceptive stimulation/ or pain threshold/ or heat pain threshold/ or pressure pain threshold/ or pain receptor/ or pain measurement/ or algometry/ or analgesia/ or antinociception/ or epidural analgesia/ or postoperative analgesia/ or (pain* or nocicept* or noxious* or allodynia* or allo-dynia* or hyperalges* or hyper-alges* or hypoalges* or hypo-alges* or analges*).ti,ab.

3 electroencephalography/ or continuous electroencephalography/ or electroencephalography monitoring/ or electroencephalogram/ or event related potential/ or evoked response/ or evoked cortical response/ or alpha rhythm/ or beta rhythm/ or delta rhythm/ or gamma rhythm/ or theta rhythm/ or hippocampus theta rhythm/ or mu rhythm/ or cortical excitability/ or laser evoked potential/ or cortical synchronization/ or electroencephalography phase synchronization/ or (eeg* or electroencephalogra* or electro-encephalogra* or erp or event-related potential* or event related potential* or evoked response* or brain activit* or brain function* or cortical activit* or cortical function*).ti,ab.

4 1 and 2 and 3

CINAHL (EBSCO Industries)

S1 (MH infant) OR (MH "infant, newborn") OR (MH "infant, low birth weight") OR (MH "infant, small for gestational age") OR (MH "infant, very low birth weight") OR (MH "infant, extremely low birth weight") OR (MH "infant, postmature") OR (MH "infant, premature") OR (MH "infant, extremely premature") OR (MH "birth weight") OR ((TI perinatal* OR AB perinatal*) OR (TI neonatal* OR AB neonatal*) OR (TI neo-natal* OR AB neo-natal*) OR (TI postnatal* OR AB postnatal*) OR (TI post-natal* OR AB post-natal*) OR (TI baby OR AB baby) OR (TI babies OR AB babies) OR (TI neonate* OR AB neonate*) OR (TI neo-nate* OR AB neo-nate*) OR (TI newborn* OR AB newborn*) OR (TI new-born* OR AB new-born*) OR (TI infant* OR AB infant*))

S2 (MH pain) OR (MH "acute pain") OR (MH "nociceptive pain") OR (MH "pain, postoperative") OR (MH "pain, procedural") OR (MH "pain perception") OR (MH nociception) OR (MH hyperalgesia) OR (MH "pain measurement") OR (MH "pain management") OR (MH "pain threshold") OR (MH nociceptors) OR (MH analgesia) OR ((TI pain* OR AB pain*) OR (TI nocicept* OR AB nocicept*) OR (TI noxious* OR AB noxious*) OR (TI allodynia* OR AB allodynia*) OR (TI allo-dynia* OR AB allo-dynia*) OR (TI hyperalges* OR AB hyperalges*) OR (TI hyper-alges* OR AB hyper-alges*) OR (TI hypoalges* OR AB hypoalges*) OR (TI hypo-alges* OR AB hypo-alges*) OR (TI analges* OR AB analges*))

S3 (MH electroencephalography) OR (MH "evoked potentials") OR (MH "laser-evoked potentials") OR (MH "cortical synchronization") OR (MH "electroencephalography phase synchronization") OR (MH "brain waves") OR (MH "alpha rhythm") OR (MH "beta rhythm") OR (MH "delta rhythm") OR (MH "gamma rhythm") OR (MH "theta rhythm") OR (MH "cortical excitability") OR ((TI eeg* OR AB eeg*) OR (TI electroencephalogra* OR AB electroencephalogra*) OR (TI electro-encephalogra* OR AB electro-encephalogra*) OR (TI erp OR AB erp) OR (TI "event-related potential*" OR AB "event-related potential*") OR (TI "event related potential*" OR AB "event related potential*") OR (TI "evoked response*" OR AB "evoked response*") OR (TI "brain activit*" OR AB "brain

activit*") OR (TI "brain function*" OR AB "brain function*") OR (TI "cortical activit*" OR AB "cortical activit*") OR (TI "cortical function*" OR AB "cortical function*"))

S4 S1 AND S2 AND S3

Web Of Science Core Collection (Clarivate Analytics)

1 ALL=infant OR ALL="infant, newborn" OR ALL="infant, low birth weight" OR ALL="infant, small for gestational age" OR ALL="infant, very low birth weight" OR ALL="infant, extremely low birth weight" OR ALL="infant, postmature" OR ALL="infant, premature" OR ALL="infant, extremely premature" OR ALL="birth weight" OR (TI=(perinatal* OR neonatal* OR neo-natal* OR postnatal* OR post-natal* OR baby OR babies OR neonate* OR neo-nate* OR newborn* OR new-born* OR infant*) OR AB=(perinatal* OR neonatal* OR neo-natal* OR postnatal* OR post-natal* OR baby OR babies OR neonate* OR neo-nate* OR newborn* OR new-born* OR infant*))

2 ALL=pain OR ALL="acute pain" OR ALL="nociceptive pain" OR ALL="pain, postoperative" OR ALL="pain, procedural" OR ALL="pain perception" OR ALL=nociception OR ALL=hyperalgesia OR ALL="pain measurement" OR ALL="pain management" OR ALL="pain threshold" OR ALL=nociceptors OR ALL=analgesia OR (TI=(pain* OR nocicept* OR noxious* OR allodynia* OR allo-dynia* OR hyperalges* OR hyper-alges* OR hypoalges* OR hypo-alges* OR analges*) OR AB=(pain* OR nocicept* OR noxious* OR allodynia* OR allo-dynia* OR hyperalges* OR hyper-alges* OR hypoalges* OR hypo-alges* OR analges*))

3 ALL=electroencephalography OR ALL="evoked potentials" OR ALL="laser-evoked potentials" OR ALL="cortical synchronization" OR ALL="electroencephalography phase synchronization" OR ALL="brain waves" OR ALL="alpha rhythm" OR ALL="beta rhythm" OR ALL="delta rhythm" OR ALL="gamma rhythm" OR ALL="theta rhythm" OR ALL="cortical excitability" OR (TI=(eeg* OR electroencephalogra* OR electro-encephalogra* OR erp OR "event-related potential*" OR "event related potential*" OR "evoked response*" OR "brain activit*" OR "brain function*" OR "cortical activit*" OR "cortical function*") OR AB=(eeg* OR electroencephalogra* OR electro-encephalogra* OR erp OR "event-related potential*" OR "event related potential*" OR "evoked response*" OR "brain activit*" OR "brain function*" OR "cortical activit*" OR "cortical function*"))

4 #1 AND #2 AND #3

Scopus (Elsevier)

(INDEXTERMS(infant) OR INDEXTERMS("infant, newborn") OR INDEXTERMS("infant, low birth weight") OR INDEXTERMS("infant, small for gestational age") OR INDEXTERMS("infant, very low birth weight") OR INDEXTERMS("infant, extremely low birth weight") OR INDEXTERMS("infant, postmature") OR INDEXTERMS("infant, premature") OR

INDEXTERMS("infant, extremely premature") OR INDEXTERMS("birth weight") OR TITLE-ABS(perinatal* OR neonatal* OR neo-natal* OR postnatal* OR post-natal* OR baby OR babies OR neonate* OR neo-nate* OR newborn* OR new-born* OR infant*)) AND (INDEXTERMS(pain) OR INDEXTERMS("acute pain") OR INDEXTERMS("nociceptive pain") OR INDEXTERMS("pain, postoperative") OR INDEXTERMS("pain, procedural") OR INDEXTERMS("pain perception") OR INDEXTERMS(nociception) OR INDEXTERMS(hyperalgesia) OR INDEXTERMS("pain measurement") OR INDEXTERMS("pain management") OR INDEXTERMS("pain threshold") OR INDEXTERMS(nociceptors) OR INDEXTERMS(analgesia) OR TITLE-ABS(pain* OR nocicept* OR noxious* OR allodynia* OR allo-dynia* OR hyperalges* OR hyper-alges* OR hypoalges* OR hypo-alges* OR analges*)) AND (INDEXTERMS(electroencephalography) OR INDEXTERMS("evoked potentials") OR INDEXTERMS("laser-evoked potentials") OR INDEXTERMS("cortical synchronization") OR INDEXTERMS("electroencephalography phase synchronization") OR INDEXTERMS("brain waves") OR INDEXTERMS("alpha rhythm") OR INDEXTERMS("beta rhythm") OR INDEXTERMS("delta rhythm") OR INDEXTERMS("gamma rhythm") OR INDEXTERMS("theta rhythm") OR INDEXTERMS("cortical excitability") OR TITLE-ABS(eeg* OR electroencephalogra* OR electro-encephalogra* OR erp OR "event-related potential*" OR "event related potential*" OR "evoked response*" OR "brain activit*" OR "brain function*" OR "cortical activit*" OR "cortical function*"))

Google Scholar (Publish or Perish)

Keywords:
(perinatal OR neonatal OR postnatal OR baby OR babies OR neonate OR newborn OR infant) AND (pain OR nociception OR noxious OR analgesia OR analgesic) AND (eeg OR electroencephalography OR electroencephalographic OR erp OR "event related potential")

Maximum number of results:
200

ClinicalTrials.gov (https://clinicaltrials.gov)

Search 1: pain AND babies
Status:
"All studies"

"Condition or disease":
pain OR nociception OR noxious OR allodynia OR hyperalgesia OR hypoalgesia OR analgesia OR allodynic OR hyperalgesic OR hypoalgesic OR analgesic

"Other terms":
perinatal OR neonatal OR postnatal OR baby OR babies OR neonate OR newborn OR infant

Search 2: pain AND EEG

Status:
“All studies”

“Condition or disease”:
pain OR nociception OR noxious OR allodynia OR hyperalgesia OR hypoalgesia OR analgesia OR allodynic OR hyperalgesic OR hypoalgesic OR analgesic

“Other terms”:
eeg OR electroencephalography OR electroencephalographic OR erp OR "event-related potential" OR "event related potential" OR "evoked response" OR "brain activity" OR "brain function" OR "cortical activity" OR "cortical function"

Intersection of searches 1 and 2: pain AND babies AND EEG
Performed external to registry using python (code for this step is publicly available here: https://github.com/lukebax/clinical-trial-registry-data-wrangling

WHO ICTRP (https://trialsearch.who.int)

Search 1: pain
pain OR nociception OR noxious OR analgesia OR analgesic

Search 2: babies
infant OR newborn OR neonate OR baby

Intersection of searches 1 and 2: pain AND babies
Performed external to registry using python (code for this step is publicly available here: https://github.com/lukebax/clinical-trial-registry-data-wrangling

Data dictionary

Supplementary table 2: Data dictionary

Variable	*Coded abbreviation*	*Explanation*
**Publication Year**	publication_year	For papers, it is the year of publication. For clinical trial registers, it is the year when the study starts (either actual or estimated).
**Country of data origin**	data_country	The country pertains to the origin of data, not the origin of the published record.
**EEG sample size**	sample_size	The number of neonates with EEG recordings during skin-breaking procedure.
**Average age at birth (postmenstrual age)**	pma_birth_avg	The average postmenstrual age (PMA) at birth (gestational age) in weeks, of participants with EEG recording during skin-breaking procedures. To get an average age, we used either the mean or the median, whichever is reported.
**Average age at study (postmenstrual age)**	pma_study_avg	The average PMA at study in weeks, of participants with EEG recording during skin-breaking procedures. To get an average age, we used either the mean or the median, whichever is reported. If the study reported this in a unit other than weeks, we converted it to weeks. If the study only reported postnatal age, we added it to the reported PMA at birth.
**Male sex ratio (percent)**	sex_male_pct	The number of males divided by the total number of male and female with EEG recording during skin-breaking procedures, multiplied by 100. If the study did not report this, for example if it also included participant without EEG recording, or if it only reported for participants that were analyzed, we extracted the male number that we could get and used it in the calculation, assuming the sex ratio reported was representative of the sex ratio of the study population with EEG recordings.
**Female sex ratio (percent)**	sex_female_pct	The number of females divided by the total number of male and female with EEG recording during skin-breaking procedures, multiplied by 100. If the study did not report this, for example if it also included participants without EEG recordings, or if it only reported for participants that were analyzed, we extracted the female number that we could get and used it in the calculation, assuming the sex ratio reported was representative of the sex ratio of the study population with EEG recordings.
**Painful (i.e. skin-breaking) procedure**	pain_procedure	All reported skin-breaking procedures that occurred during EEG recording, standardizing terminology where possible (e.g. heel lance and heel stick were standardized to heel lance).
**Analgesic intervention**	analgesic_intervention	All pain-relief interventions studied (if any), both pharmacological and non-pharmacological, during EEG recording of skin-breaking procedures.

**Electrode placement method**	electrode_placement_method	The method of EEG electrode placement. If electrodes were placed individually, we record it as ‘individual electrodes’. If an electrode cap was used, we record it as ‘cap’. If something other than individual EEG electrodes or an EEG cap was used, we record this as ‘Other’. If electrode positions were reported, and there is no mention of using cap, we assume it is using individual electrodes.
**Electrode placement system**	electrode_placement_system	The system of electrode placement, e.g. 10-10 system, 10-20 system, 128-channel Geodesic system.
**Electrode placement position**	electrode_positions	The positions of reference and active electrodes during EEG recording, standardized according to the current version of the 10-10 system. For studies using EEG cap, we recorded the positions mentioned in the paper.
**EEG data lost due to quality issues (percent)**	eeg_data_loss_artefact	The percentage of participants excluded from subsequent analyses due to artefact among participants with EEG data recorded during skin-breaking procedures. If a paper only reported the number of participants analyzed, without mentioning the number of participants recruited, we put ‘Not provided’.
**Epoch rejection method**	epoch_rej_method	The methods by which researchers assessed EEG data with artefact as a basis for participant exclusion from subsequent analyses. We recorded it as ‘Objective’ if it is independent on the judgement of a rater (e.g. amplitude above a defined threshold), ‘Subjective’ if it is dependent on the judgement of a rater (e.g., by visual assessment), ‘Mixed’ if it is a combination of both (e.g., a certain amplitude threshold was used, but was then re-evaluated by a reviewer), ‘Not provided’ if the paper only stated that epochs were rejected if they contain artefact, but did not elaborate on how they decided that it was an artefact.
**Amplitude threshold**	amplitude_threshold	The amplitude threshold used in studies that used amplitude as the basis for participant exclusion from subsequent analyses.
**Clinical pain scale**	clinical_pain_scale	The name of clinical pain scale(s) analyzed during skin-breaking procedures alongside EEG recording, if any. If only part of the clinical pain scales was used (e.g., the facial expression component of PIPP-R, 7-criteria out of 10 of NFCS), we do not consider this as implementing the clinical pain scales.
**Non-EEG recordings**	non_eeg_recording	The type of data recorded during skin-breaking procedures alongside EEG recording, if any. If a study reported that an ECG was recorded, we put ‘Heart rate’ for standardization. This includes recording used to generate clinical pain scale(s), if the study analyzed clinical pain scale(s).

Supplementary Results

Non-primary publications excluded from synthesis

Supplementary Table 3: Studies with multiple publication types excluded from synthesis, sorted by PRISMA flow diagram categories (n=21 studies).

**Trial registrations with published papers: n=8**
1. Benoit B. The Influence of Breastfeeding on Cortical Activity During Procedures (iCAP).; 2017. https://clinicaltrials.gov/study/NCT03272594
2. Campbell-Yeo M. The Influence of Skin-to-Skin Contact on Cortical Activity During Painful Procedures on Preterm Infants in the NICU (iCAPmini).; 2018. https://clinicaltrials.gov/study/NCT03745963
3. Dempsey G. The Effect of a Musical Intervention on Stress Response to Venepuncture.; 2017. https://clinicaltrials.gov/study/NCT03028844
4. Meek J. A Clinical Study Investigating Sucrose as a Pain Reliever in Infants.; 2009. doi:10.1186/ISRCTN78390996
5. Relland LM. Effect of a Vibratory Stimulus on Mitigating Nociception-Specific Responses to Skin Puncture in Neonates.; 2018. https://clinicaltrials.gov/study/NCT04050384
6. Slater R. A Blinded Randomised Placebo-Controlled Trial Investigating the Efficacy of Morphine Analgesia for Procedural Pain in Infants.; 2015. https://www.clinicaltrialsregister.eu/ctr-search/search?query=eudract_number:2014-003237-25
7. Slater R. Parental Touch Trial (Petal) (Petal).; 2021. https://clinicaltrials.gov/study/NCT04901611
8. Slater R. Using Parental Touch to Relieve Pain in Newborn Infants.; 2021. doi:10.1186/ISRCTN14135962
**Theses with published papers: n=6**
9. Aspbury M. Developing Neuroimaging Methods for Clinical Translation and Better Understanding Neonatal Brain Development. http://purl.org/dc/dcmitype/Text. University of Oxford; 2023. Accessed February 23, 2024. https://ora.ox.ac.uk/objects/uuid:3ff54503-a5cc-48bd-8518-6e018efa8905
10. Bucsea O. Examining the Relationships Between Neonatal Pain-Related Facial Actions and Cortical Activity. York University; 2020. Accessed August 18, 2024. http://hdl.handle.net/10315/37946
11. Cobo MM. Developing Translational Tools for Measuring Pain in Neonates. http://purl.org/dc/dcmitype/Text. University of Oxford; 2022. Accessed February 23, 2024. https://ora.ox.ac.uk/objects/uuid:c3d39756-706c-4bad-816f-88fdadf92c3b
12. Goksan S. Imaging Nociceptive Brain Activity in the Newborn Infant. http://purl.org/dc/dcmitype/Text. University of Oxford; 2016. Accessed August 18, 2024. https://ora.ox.ac.uk/objects/uuid:ea4d49fc-cf7e-4775-bb82-ddb3385cc2d9
13. Green G. Measuring Pain in the Newborn Infant. http://purl.org/dc/dcmitype/Text. University of Oxford; 2018. Accessed August 18, 2024. https://ora.ox.ac.uk/objects/uuid:5647e78c-48fb-4b1d-a54f-146803bd7037
14. van der Vaart ML. Multimodal Assessment of Neonatal Pain. http://purl.org/dc/dcmitype/Text. University of Oxford; 2022. Accessed February 23, 2024. https://ora.ox.ac.uk/objects/uuid:c18e5c7d-e36e-440b-851f-c8cf1393a95c
**Conference abstracts with published papers: n=2**

15. Jones L, Verriotis M, Laudiano-Dray M, et al. Behavioural and cortical pain responses in human infants are dissociable by their relationship to physiological stress. British Neuroscience Association Festival of Neuroscience, BNA 2017 Birmingham United Kingdom. 2017;1:144. doi:10.1177/2398212817705279
16. Menser CCM, Bruehl S, France D, Wilkes D, Maitre N. Assessment of neonatal pain: Correlations between cortical somatosensory processing and cry acoustics. Anesthesia & Analgesia. 2016;122(5 Supplement 3):S245. doi:10.1213/01.ane.0000499505.96779.a0
**Preprints subsequently published: n=2**
17. Rupawala M, Bucsea O, Laudiano-Dray MP, et al. Developmental switch in prediction and adaptation to pain in human neonates. Published online April 7, 2022:2022.04.05.486988. doi:10.1101/2022.04.05.486988
18. Talebi S, Frounchi J, Tazehkand BM. A Novel Channel Selection Approach for Human Neonate's Pain EEG Data Analysis. Published online December 28, 2022. doi:10.21203/rs.3.rs-2390234/v1
**Trial reports: n=1**
19. Monk V, Moultrie F, Hartley C, et al. Oral morphine analgesia for preventing pain during invasive procedures in non-ventilated premature infants in hospital: the Poppi RCT. Efficacy and Mechanism Evaluation. 2019;6(9):1-98. doi:10.3310/eme06090
**Protocols for completed and published trials: n=1**
20. Cobo MM, Moultrie F, Hauck AGV, et al. Multicentre, randomised controlled trial to investigate the effects of parental touch on relieving acute procedural pain in neonates (Petal). BMJ Open. 2022;12(7):e061841. doi:10.1136/bmjopen-2022-061841
**SAPs for completed and published trials: n=1**
21. Baxter L, Hauck AGV, Bhatt A, et al. Statistical analysis plan for the Petal trial: the effects of parental touch on relieving acute procedural pain in neonates. Wellcome Open Res. 2023;8:402. doi:10.12688/wellcomeopenres.19819.3

Primary publications included in synthesis

Supplementary Table 4: Unique primary representative studies included in synthesis, sorted by PRISMA flow diagram categories (n=55 studies). *Corresponding author highlighted in yellow. When no corresponding author was listed, or if the corresponding author was otherwise not contactable, the last author has been highlighted in blue.*

**Reports without analyzable EEG details: n=11**
Trial registrations without published papers: n=7
1. Elisabeth Norman. Fentanyl and Clonidine for Analgesia During Hypothermia in Term Asphyxiated Infants (SANNI 1).; 2017. https://clinicaltrials.gov/study/NCT03177980
2. Elisabeth Norman. Clonidine for Analgesia to Preterm Infants During Neonatal Intensive Care.; 2018. https://clinicaltrials.gov/study/NCT04928651
3. Laura Cornelissen. Innovative Approaches to Assessment of Pain Control and Sedation in the NICU.; 2016. https://clinicaltrials.gov/study/NCT03057782
4. Pierre Kuhn. Multimodal Approach to the Ontogenesis of Nociception in Very Preterm and Term Infants (NOCI-Prem).; 2019. https://clinicaltrials.gov/study/NCT05404594
5. Rebecca Pillai Riddell. Rebooting Infant Pain Assessment: Using Machine Learning to Exponentially Improve Neonatal Intensive Care Unit Practice (BabyAI).; 2020. https://clinicaltrials.gov/study/NCT05579496
6. Sonya Wang. Effects of Music Based Intervention (MBI) on Pain Response and Neurodevelopment in Preterm Infants.; 2020. https://clinicaltrials.gov/study/NCT04286269
7. Vineta Fellman. NeoFent-I Study; Fentanyl Treatment in Newborn Infants; a Pharmacokinetic, Pharmacodynamic and Pharmacogenetic Study.; 2012. https://clinicaltrials.gov/study/NCT03897452
Published papers: n=4
8. Alan Worley, Lorenzo Fabrizi, Stewart Boyd, Rebeccah Slater. Multi-modal pain measurements in infants. J Neurosci Methods. 2012;205(2):252-257. doi:10.1016/j.jneumeth.2012.01.009
9. Alan Worley, Kirubin Pillay, Maria Cobo, Gabriela Schmidt Mellado; Marianne van der Vaart; Aomesh Bhatt, Caroline Hartley. The PiNe box: Development and validation of an electronic device to time-lock multimodal responses to sensory stimuli in hospitalised infants. PLOS ONE. 2023;18(7):e0288488. doi:10.1371/journal.pone.0288488
10. Lorenzo Fabrizi, Alan Worley, Debbie Patten, Siân Roberts-Holdridge, Laura Cornelissen, Judith Meek, Stewart Boyd, Rebeccah Slater. Electrophysiological Measurements and Analysis of Nociception in Human Infants. JoVE (Journal of Visualized Experiments). 2011;(58):e3118. doi:10.3791/3118
11. Marsha Campbell-Yeo, Britney Benoit, Aaron Newman, Celeste Johnston, Tim Bardouille, Bonnie Stevens, Arlene Jiang. The influence of skin-to-skin contact on Cortical Activity during Painful procedures in preterm infants in the neonatal intensive care unit (iCAP mini): study protocol for a randomized control trial. Trials. 2022;23(1):512. doi:10.1186/s13063-022-06424-4
**Reports containing analyzable EEG details: n=44**
Published papers: n=44
12. Andreea Pavel, Farah Abu Dhais, Caoimhe Howard, John O'Toole, Elena Pavlidis, Daragh Finn, Vicki Livingstone, Anna Powell, Eugene Dempsey, Geraldine Boylan. GP252 The effect of music therapy on the electroencephalogram (EEG) and heart rate variability (HRV) of premature infants during routine painful

procedures. Archives of Disease in Childhood. 2019;104(Suppl 3):A135-A135. doi:10.1136/archdischild-2019-epa.311

13. Annalisa Hauck, Marianne van der Vaart, Eleri Adams, Luke Baxter, Aomesh Bhatt, Daniel Crankshaw, Amraj Dhami, Ria Evans Fry, Marina Freire, Caroline Hartley, Roshni Mansfield, Simon Marchant, Vaneesha Monk, Fiona Moultrie, Mariska Peck, Shellie Robinson, Jean Yong, Ravi Poorun, Maria Cobo, Rebeccah Slater. Effect of parental touch on relieving acute procedural pain in neonates and parental anxiety (Petal): a multicentre, randomised controlled trial in the UK. The Lancet Child & Adolescent Health. 2024;8(4):259-269. doi:10.1016/S2352-4642(23)00340-1

14. Britney Benoit, Aaron Newman, Ruth Martin-Misener, Margot Latimer, Marsha Campbell-Yeo. The influence of breastfeeding on cortical and bio-behavioural indicators of procedural pain in newborns: Findings of a randomized controlled trial. Early Human Development. 2021;154:105308. doi:10.1016/j.earlhumdev.2021.105308

15. Caroline Hartley, Sezgi Goksan, Ravi Poorun, Kelly Brotherhood, Gabriela Schmidt Mellado, Fiona Moultrie, Richard Rogers, Eleri Adams, Rebeccah Slater. The relationship between nociceptive brain activity, spinal reflex withdrawal and behaviour in newborn infants. Sci Rep. 2015;5(1):12519. doi:10.1038/srep12519

16. Caroline Hartley, Fiona Moultrie, Deniz Gursul, Amy Hoskin, Eleri Adams, Richard Rogers, Rebeccah Slater. Changing Balance of Spinal Cord Excitability and Nociceptive Brain Activity in Early Human Development. Current Biology. 2016;26(15):1998-2002. doi:10.1016/j.cub.2016.05.054

17. Caroline Hartley, Eugene Duff, Gabrielle Green, Gabriela Schmidt Mellado, Alan Worley, Richard Rogers, Rebeccah Slater. Nociceptive brain activity as a measure of analgesic efficacy in infants. Science Translational Medicine. 2017;9(388):eaah6122. doi:10.1126/scitranslmed.aah6122

18. Caroline Hartley, Fiona Moultrie, Amy Hoskin, Gabrielle Green, Vaneesha Monk, Jennifer Bell, Andrew King, Miranda Buckle, Marianne van der Vaart, Deniz Gursul, Sezgi Goksan, Edmund Juszczak, Jane Norman, Richard Rogers, Chetan Patel, Eleri Adams, Rebeccah Slater. Analgesic efficacy and safety of morphine in the Procedural Pain in Premature Infants (Poppi) study: randomised placebo-controlled trial. The Lancet. 2018;392(10164):2595-2605. doi:10.1016/S0140-6736(18)31813-0

19. Caroline Hartley, Luke Baxter, Fiona Moultrie, Ryan Purdy, Aomesh Bhatt, Richard Rogers, Chetan Patel, Eleri Adams, Rebeccah Slater. Predicting severity of adverse cardiorespiratory effects of morphine in premature infants: a post hoc analysis of Procedural Pain in Premature Infants trial data. British Journal of Anaesthesia. 2021;126(4):e133-e135. doi:10.1016/j.bja.2020.10.034

20. Caterina Coviello, Silvia Lori, Giovanna Bertini, Simona Montano, Simonetta Gabbanini, Maria Bastianelli, Cesarina Cossu, Sara Cavaliere, Clara Lunardi, Carlo Dani. Evaluation of the Relationship between Pain Exposure and Somatosensory Evoked Potentials in Preterm Infants: A Prospective Cohort Study. Children. 2024;11(6):676. doi:10.3390/children11060676

21. Deniz Gursul, Sezgi Goksan, Caroline Hartley, Gabriela Schmidt Mellado, Fiona Moultrie, Amy Hoskin, Eleri Adams, Gareth Hathway, Susannah Walker, Francis McGlone, Rebeccah Slater. Stroking modulates noxious-evoked brain activity in human infants. Current Biology. 2018;28(24):R1380-R1381. doi:10.1016/j.cub.2018.11.014

22. Elisabeth Norman, Ingmar Rosen, Sampsa Vanhatalo, Karin Stjernqvist, Ove Okland, Vineta Fellman, Lena Hellstrom-Westas. Electroencephalographic Response to Procedural Pain in Healthy Term Newborn Infants. Pediatr Res. 2008;64(4):429-434. doi:10.1203/PDR.0b013e3181825487

23. Gabriela Schmidt Mellado, Kirubin Pillay, Eleri Adams, Ana Alarcon, Foteini Andritsou, Maria Cobo, Ria Evans Fry, Sean Fitzgibbon, Fiona Moultrie, Luke Baxter, Rebeccah Slater. The impact of premature extrauterine exposure on infants' stimulus-evoked brain activity across multiple sensory systems. NeuroImage: Clinical. 2022;33:102914. doi:10.1016/j.nicl.2021.102914

24. Gabrielle Green, Caroline Hartley, Amy Hoskin, Eugene Duff, Adam Shriver, Dominic Wilkinson, Eleri Adams, Richard Rogers, Fiona Moultrie, Rebeccah Slater. Behavioural discrimination of noxious stimuli in infants is dependent on brain maturation. PAIN. 2019;160(2):493. doi:10.1097/j.pain.0000000000001425

25. Hisham Abdelsami Awad, Sahar Hassanein, Rania Mohamed Abdou, L Taher Bassiouny. Analysis of pain effect on EEG recordings and oral sucrose suckling effect on pain reduction in neonates. QJM: An International Journal of Medicine. 2018;111(suppl_1):hcy200.158. doi:10.1093/qjmed/hcy200.158

26. Jean-Michel Roué, Amir Avnit, Behnood Gholami, Wassim Haddad, Kanwaljeet Anand. Objective Detection of Newborn Infant Acute Procedural Pain Using EEG and Machine Learning Algorithms. Paediatric and Neonatal Pain. 2025;7(1):e70001. doi:10.1002/pne2.70001

27. Lance Relland, Caitlin Kjeldsen, Arnaud Jeanvoine, Lelia Emery, Kathleen Adderley, Rachelle Srinivas, Maeve McLoughlin, Nathalie Maitre. Vibration-based mitigation of noxious-evoked responses to skin puncture in neonates and infants: a randomised controlled trial. Archives of Disease in Childhood - Fetal and Neonatal Edition. Published online March 13, 2024. doi:10.1136/archdischild-2023-326588

28. Laura Jones, Lorenzo Fabrizi, Maria Laudiano-Dray, Kimberley Whitehead, Judith Meek, Madeleine Verriotis, Maria Fitzgerald. Nociceptive Cortical Activity Is Dissociated from Nociceptive Behavior in Newborn Human Infants under Stress. Current Biology. 2017;27(24):3846-3851.e3. doi:10.1016/j.cub.2017.10.063

29. Laura Jones, Maria Laudiano-Dray, Kimberley Whitehead, Madeleine Verriotis, Judith Meek, Maria Fitzgerald, Lorenzo Fabrizi. EEG, behavioural and physiological recordings following a painful procedure in human neonates. Sci Data. 2018;5(1):180248. doi:10.1038/sdata.2018.248

30. Laura Jones, Maria Laudiano-Dray, Kimberley Whitehead, Judith Meek, Maria Fitzgerald, Lorenzo Fabrizi, Rebecca Pillai Riddell. The impact of parental contact upon cortical noxious-related activity in human neonates. European Journal of Pain. 2021;25(1):149-159. doi:10.1002/ejp.1656

31. Lorenzo Fabrizi, Rebeccah Slater, Alan Worley, Judith Meek, Sofia Olhede, Stewart Boyd, Maria Fitzgerald. P14-24 Development of a cortical electrophysiological response to noxious stimulation in human infants. Clinical Neurophysiology. 2010;121:S190. doi:10.1016/S1388-2457(10)60782-1

32. Lorenzo Fabrizi, Rebeccah Slater, Alan Worley, Judith Meek, Stewart Boyd, Sofia Olhede, Maria Fitzgerald. A Shift in Sensory Processing that Enables the Developing Human Brain to Discriminate Touch from Pain. Current Biology. 2011;21(18):1552-1558. doi:10.1016/j.cub.2011.08.010

33. Lorenzo Fabrizi, Madeleine Verriotis, Gemma Williams, Amy Lee, Judith Meek, Sofia Olhede, Maria Fitzgerald. Encoding of mechanical nociception differs in the adult and infant brain. Sci Rep. 2016;6(1):28642. doi:10.1038/srep28642

34. Madeleine Verriotis, Lorenzo Fabrizi, Amy Lee, Sheryl Ledwidge, Judith Meek, Maria Fitzgerald. Cortical activity evoked by inoculation needle prick in infants up to one-year old. Pain. 2015;156(2):222-230. doi:10.1097/01.j.pain.0000460302.56325.0c

35. Madeleine Verriotis, Lorenzo Fabrizi, Amy Lee, Robert Cooper, Maria Fitzgerald, Judith Meek. Mapping Cortical Responses to Somatosensory Stimuli in Human Infants with Simultaneous Near-Infrared Spectroscopy and Event-Related Potential Recording. eNeuro. 2016;3(2). doi:10.1523/ENEURO.0026-16.2016

36. Madeleine Verriotis, Laura Jones, Kimberley Whitehead, Maria Laudiano-Dray, Ismini Panayotidis, Hemani Patel, Judith Meek, Lorenzo Fabrizi, Maria Fitzgerald. The distribution of pain activity across the human neonatal brain is sex dependent. NeuroImage. 2018;178:69-77. doi:10.1016/j.neuroimage.2018.05.030

37. Maria Cobo, Caroline Hartley, Deniz Gursul, Foteini Andritsou, Marianne van der Vaart, Gabriela Schmidt Mellado, Luke Baxter, Eugene Duff, Miranda Buckle, Ria Evans Fry, Gabrielle Green, Amy Hoskin, Richard Rogers, Eleri Adams, Fiona Moultrie, Rebeccah Slater. Quantifying noxious-evoked baseline sensitivity in neonates to optimise analgesic trials. Ploner M, Büchel C, Tibboel D, Ploner M, eds. eLife. 2021;10:e65266. doi:10.7554/eLife.65266

38. Maria Cobo, Gabrielle Green, Foteini Andritsou, Luke Baxter, Ria Evans Fry, Annika Grabbe, Deniz Gursul, Amy Hoskin, Gabriela Schmidt Mellado, Marianne van der Vaart, Eleri Adams, Aomesh Bhatt, Franziska Denk, Caroline Hartley, Rebeccah Slater. Early life inflammation is associated with spinal cord excitability and nociceptive sensitivity in human infants. Nat Commun. 2022;13(1):3943. doi:10.1038/s41467-022-31505-y

39. Marianne Aspbury, Roshni Mansfield, Luke Baxter, Aomesh Bhatt, Maria Cobo, Sean Fitzgibbon, Caroline Hartley, Annalisa Hauck, Simon Marchant, Vaneesha Monk, Kirubin Pillay, Ravi Poorun, Marianne van der Vaart, Rebeccah Slater. Establishing a standardised approach for the measurement of neonatal noxious-evoked brain activity in response to an acute somatic nociceptive heel lance stimulus. Cortex. 2024;179:215-234. doi:10.1016/j.cortex.2024.05.023

40. Marianne van der Vaart, Eugene Duff, Nader Raafat, Richard Rogers, Caroline Hartley, Rebeccah Slater. Multimodal pain assessment improves discrimination between noxious and non-noxious stimuli in infants. Paediatric and Neonatal Pain. 2019;1(1):21-30. doi:10.1002/pne2.12007

41. Marianne van der Vaart, Caroline Hartley, Luke Baxter, Gabriela Schmidt Mellado, Foteini Andritsou, Maria Cobo, Ria Evans Fry, Eleri Adams, Sean Fitzgibbon, Rebeccah Slater. Premature infants display discriminable behavioral, physiological, and brain responses to noxious and nonnoxious stimuli. Cerebral Cortex. 2022;32(17):3799-3815. doi:10.1093/cercor/bhab449

42. Mohammed Rupawala, Oana Bucsea, Maria Laudiano-Dray, Kimberley Whitehead, Judith Meek, Maria Fitzgerald, Sofia Olhede, Laura Jones, Lorenzo Fabrizi. A developmental shift in habituation to pain in human neonates. Current Biology. 2023;33(8):1397-1406.e5. doi:10.1016/j.cub.2023.02.071

43. Nathalie Maitre, Ann Stark, Carrie McCoy Menser, Olena Chorna, Daniel France, Alexandra Key, Ken Wilkens, Melissa Moore-Clingenpeel, Don Wilkes, Stephen Bruehl. Cry presence and amplitude do not reflect cortical processing of painful stimuli in newborns with distinct responses to touch or cold. Arch Dis Child Fetal Neonatal Ed. 2017;102(5):F428-F433. doi:10.1136/archdischild-2016-312279

44. Neta Maimon, Ruth Grunau, Ivan Cepeda, Michael Friger, Leonel Selnovik, Shlomo Gilat, Eilon Shany. Electroencephalographic Activity in Response to Procedural Pain in Preterm Infants Born at 28 and 33 Weeks Gestational Age. The Clinical Journal of Pain. 2013;29(12):1044. doi:10.1097/AJP.0b013e318284e525

45. Nusreena Hohsoh, Osuke Iwata, Tomoko Suzuki, Chinami Hanai, Ming Huang, Kiyoko Yokoyama. Quantification electroencephalography response to procedural pain during heel puncture in preterm infants. Physiol Meas. 2025;46(6):065004. doi:10.1088/1361-6579/addfa9

46. Oana Bucsea, Mohammed Rupawala, Ilana Shiff, Xiaogang Wang, Judith Meek, Maria Fitzgerald, Lorenzo Fabrizi, Rebecca Pillai Riddell, Laura Jones. Clinical thresholds in pain-related facial activity linked to differences in cortical network activation in neonates. PAIN. 2023;164(5):1039. doi:10.1097/j.pain.0000000000002798

47. Paul Castillo, Sampsa Vanhatalo, Marit Lundblad, Mats Blennow, Per-Arne Lonnqvist. EEG response to a high volume (1.5 mL/kg) caudal block in infants less than 3 months. Reg Anesth Pain Med. 2023;49(3):163-167. doi:10.1136/rapm-2023-104452

48. Rebeccah Slater, Alan Worley, Lorenzo Fabrizi, Siân Roberts-Holdridge, Judith Meek, Stewart Boyd, Maria Fitzgerald. Evoked potentials generated by noxious stimulation in the human infant brain. European Journal of Pain. 2010;14(3):321-326. doi:10.1016/j.ejpain.2009.05.005

49. Rebeccah Slater, Lorenzo Fabrizi, Alan Worley, Judith Meek, Stewart Boyd, Maria Fitzgerald. Premature infants display increased noxious-evoked neuronal activity in the brain compared to healthy age-matched term-born infants. NeuroImage. 2010;52(2):583-589. doi:10.1016/j.neuroimage.2010.04.253

50. Rebeccah Slater, Laura Cornelissen, Lorenzo Fabrizi, Debbie Patten, Jan Yoxen, Alan Worley, Stewart Boyd, Judith Meek, Maria Fitzgerald. Oral sucrose as an analgesic drug for procedural pain in newborn infants: a randomised controlled trial. The Lancet. 2010;376(9748):1225-1232. doi:10.1016/S0140-6736(10)61303-7

51. Reyhane Shafiee, Mohammad Reza Daliri. Decoding of pain during heel lancing in human neonates with EEG signal and machine learning approach. Sci Rep. 2024;14(1):31244. doi:10.1038/s41598-024-82631-0

52. Safa Talebi, Javad Frounchi, Behzad Mozaffari Tazehkand. A novel channel selection approach for human neonate's pain EEG data analysis. SIViP. 2025;19(5):364. doi:10.1007/s11760-025-03934-x

53. Shahbaz Askari, Zoya Bastany, Liisa Holsti, Guy Dumont. Lighting up babies' brains: development of a combined NIRS/EEG system for infants. In: Biophotonics in Exercise Science, Sports Medicine, Health Monitoring Technologies, and Wearables II. Vol 11638. SPIE; 2021:80-85. doi:10.1117/12.2595899

54. Simon Marchant, Marianne van der Vaart, Kirubin Pillay, Luke Baxter, Aomesh Bhatt, Sean Fitzgibbon, Caroline Hartley, Rebeccah Slater. A machine learning artefact detection method for single-channel infant event-related potential studies. J Neural Eng. 2024;21(4):046021. doi:10.1088/1741-2552/ad5c04

55. Sofie Nilsson, Anton Tokariev, Timo Vehviläinen, Vineta Fellman, Sampsa Vanhatalo, Elisabeth Norman. Depression of cortical neuronal activity after a low-dose fentanyl in preterm infants. Acta Paediatrica. 2025;114(1):109-115. doi:10.1111/apa.17411

Authors to contact

Supplementary Table 5: Unique primary representative studies included in synthesis, sorted by author to contact (n=55 studies, N=31 authors to contact). *Corresponding author highlighted in yellow. When no corresponding author was listed, the last author has been highlighted in blue.*

Author to contact	Publications
**1 & 2. Behnood Gholami & Kanwaljeet J. S. Anand**	26. Jean-Michel Roué, Amir Avnit, Behnood Gholami, Wassim Haddad, Kanwaljeet J. S. Anand. Objective Detection of Newborn Infant Acute Procedural Pain Using EEG and Machine Learning Algorithms. Paediatric and Neonatal Pain. 2025;7(1):e70001. doi:10.1002/pne2.70001
**3. Britney Benoit**	14. Britney Benoit, Aaron Newman, Ruth Martin-Misener, Margot Latimer, Marsha Campbell-Yeo. The influence of breastfeeding on cortical and bio-behavioural indicators of procedural pain in newborns: Findings of a randomized controlled trial. Early Human Development. 2021;154:105308. doi:10.1016/j.earlhumdev.2021.105308
**4. Caroline Hartley**	9. Alan Worley, Kirubin Pillay, Maria Cobo, Gabriela Schmidt Mellado; Marianne van der Vaart; Aomesh Bhatt, Caroline Hartley. The PiNe box: Development and validation of an electronic device to time-lock multimodal responses to sensory stimuli in hospitalised infants. PLOS ONE. 2023;18(7):e0288488. doi:10.1371/journal.pone.0288488<br><br>15. Caroline Hartley, Sezgi Goksan, Ravi Poorun, Kelly Brotherhood, Gabriela Schmidt Mellado, Fiona Moultrie, Richard Rogers, Eleri Adams, Rebeccah Slater. The relationship between nociceptive brain activity, spinal reflex withdrawal and behaviour in newborn infants. Sci Rep. 2015;5(1):12519. doi:10.1038/srep12519<br><br>19. Caroline Hartley, Luke Baxter, Fiona Moultrie, Ryan Purdy, Aomesh Bhatt, Richard Rogers, Chetan Patel, Eleri Adams, Rebeccah Slater. Predicting severity of adverse cardiorespiratory effects of morphine in premature infants: a post hoc analysis of Procedural Pain in Premature Infants trial data. British Journal of Anaesthesia. 2021;126(4):e133-e135. doi:10.1016/j.bja.2020.10.034<br><br>40. Marianne van der Vaart, Eugene Duff, Nader Raafat, Richard Rogers, Caroline Hartley, Rebeccah Slater. Multimodal pain assessment improves discrimination between noxious and non-noxious stimuli in infants. Paediatric and Neonatal Pain. 2019;1(1):21-30. doi:10.1002/pne2.12007
**5. Caterina Coviello**	20. Caterina Coviello, Silvia Lori, Giovanna Bertini, Simona Montano, Simonetta Gabbanini, Maria Bastianelli, Cesarina Cossu, Sara Cavaliere, Clara Lunardi, Carlo Dani. Evaluation of the Relationship between Pain Exposure and Somatosensory Evoked Potentials in Preterm Infants: A Prospective Cohort Study. Children. 2024;11(6):676. doi:10.3390/children11060676
**6. Eilon Shany**	44. Neta Maimon, Ruth Grunau, Ivan Cepeda, Michael Friger, Leonel Selnovik, Shlomo Gilat, Eilon Shany. Electroencephalographic Activity in Response to Procedural Pain in Preterm Infants Born at 28 and 33 Weeks Gestational Age. The Clinical Journal of Pain. 2013;29(12):1044. doi:10.1097/AJP.0b013e318284e525

**7. Elisabeth Norman**	1. Elisabeth Norman. Fentanyl and Clonidine for Analgesia During Hypothermia in Term Asphyxiated Infants (SANNI 1).; 2017. https://clinicaltrials.gov/study/NCT03177980<br><br>2. Elisabeth Norman. Clonidine for Analgesia to Preterm Infants During Neonatal Intensive Care.; 2018. https://clinicaltrials.gov/study/NCT04928651<br><br>22. Elisabeth Norman, Ingmar Rosen, Sampsa Vanhatalo, Karin Stjernqvist, Ove Okland, Vineta Fellman, Lena Hellstrom-Westas. Electroencephalographic Response to Procedural Pain in Healthy Term Newborn Infants. Pediatr Res. 2008;64(4):429-434. doi:10.1203/PDR.0b013e3181825487
**8. Geraldine Boylan**	12. Andreea Pavel, Farah Abu Dhais, Caoimhe Howard, John O'Toole, Elena Pavlidis, Daragh Finn, Vicki Livingstone, Anna Powell, Eugene Dempsey, Geraldine Boylan. GP252 The effect of music therapy on the electroencephalogram (EEG) and heart rate variability (HRV) of premature infants during routine painful procedures. Archives of Disease in Childhood. 2019;104(Suppl 3):A135-A135. doi:10.1136/archdischild-2019-epa.311
**9. Guy Dumont**	53. Shahbaz Askari, Zoya Bastany, Liisa Holsti, Guy Dumont. Lighting up babies' brains: development of a combined NIRS/EEG system for infants. In: Biophotonics in Exercise Science, Sports Medicine, Health Monitoring Technologies, and Wearables II. Vol 11638. SPIE; 2021:80-85. doi:10.1117/12.2595899
**10. Hisham Abdelsami Awad**	25. Hisham Abdelsami Awad, Sahar Hassanein, Rania Mohamed Abdou, L Taher Bassiouny. Analysis of pain effect on EEG recordings and oral sucrose suckling effect on pain reduction in neonates. QJM: An International Journal of Medicine. 2018;111(suppl_1):hcy200.158. doi:10.1093/qjmed/hcy200.158
**11. Judith Meek**	35. Madeleine Verriotis, Lorenzo Fabrizi, Amy Lee, Robert Cooper, Maria Fitzgerald, Judith Meek. Mapping Cortical Responses to Somatosensory Stimuli in Human Infants with Simultaneous Near-Infrared Spectroscopy and Event-Related Potential Recording. eNeuro. 2016;3(2). doi:10.1523/ENEURO.0026-16.2016
**12. Kiyoko Yokoyama**	45. Nusreena Hohsoh, Osuke Iwata, Tomoko Suzuki, Chinami Hanai, Ming Huang, Kiyoko Yokoyama. Quantification electroencephalography response to procedural pain during heel puncture in preterm infants. Physiol Meas. 2025;46(6):065004. doi:10.1088/1361-6579/addfa9
**13. Laura Cornelissen**	3. Laura Cornelissen. Innovative Approaches to Assessment of Pain Control and Sedation in the NICU.; 2016. https://clinicaltrials.gov/study/NCT03057782
**14. Laura Jones**	29. Laura Jones, Maria Laudiano-Dray, Kimberley Whitehead, Madeleine Verriotis, Judith Meek, Maria Fitzgerald, Lorenzo Fabrizi. EEG, behavioural and physiological recordings following a painful procedure in human neonates. Sci Data. 2018;5(1):180248. doi:10.1038/sdata.2018.248<br><br>46. Oana Bucsea, Mohammed Rupawala, Ilana Shiff, Xiaogang Wang, Judith Meek, Maria Fitzgerald, Lorenzo Fabrizi, Rebecca Pillai Riddell, Laura Jones. Clinical thresholds in pain-related facial activity linked to differences in cortical network activation in neonates. PAIN. 2023;164(5):1039. doi:10.1097/j.pain.0000000000002798

**15. Lorenzo Fabrizi**	30. Laura Jones, Maria Laudiano-Dray, Kimberley Whitehead, Judith Meek, Maria Fitzgerald, Lorenzo Fabrizi, Rebecca Pillai Riddell. The impact of parental contact upon cortical noxious-related activity in human neonates. European Journal of Pain. 2021;25(1):149-159. doi:10.1002/ejp.1656<br><br>32. Lorenzo Fabrizi, Rebeccah Slater, Alan Worley, Judith Meek, Stewart Boyd, Sofia Olhede, Maria Fitzgerald. A Shift in Sensory Processing that Enables the Developing Human Brain to Discriminate Touch from Pain. Current Biology. 2011;21(18):1552-1558. doi:10.1016/j.cub.2011.08.010<br><br>33. Lorenzo Fabrizi, Madeleine Verriotis, Gemma Williams, Amy Lee, Judith Meek, Sofia Olhede, Maria Fitzgerald. Encoding of mechanical nociception differs in the adult and infant brain. Sci Rep. 2016;6(1):28642. doi:10.1038/srep28642<br><br>42. Mohammed Rupawala, Oana Bucsea, Maria Laudiano-Dray, Kimberley Whitehead, Judith Meek, Maria Fitzgerald, Sofia Olhede, Laura Jones, Lorenzo Fabrizi. A developmental shift in habituation to pain in human neonates. Current Biology. 2023;33(8):1397-1406.e5. doi:10.1016/j.cub.2023.02.071
**16. Luke Baxter**	23. Gabriela Schmidt Mellado, Kirubin Pillay, Eleri Adams, Ana Alarcon, Foteini Andritsou, Maria Cobo, Ria Evans Fry, Sean Fitzgibbon, Fiona Moultrie, Luke Baxter, Rebeccah Slater. The impact of premature extrauterine exposure on infants' stimulus-evoked brain activity across multiple sensory systems. NeuroImage: Clinical. 2022;33:102914. doi:10.1016/j.nicl.2021.102914
**17. Maria Fitzgerald**	28. Laura Jones, Lorenzo Fabrizi, Maria Laudiano-Dray, Kimberley Whitehead, Judith Meek, Madeleine Verriotis, Maria Fitzgerald. Nociceptive Cortical Activity Is Dissociated from Nociceptive Behavior in Newborn Human Infants under Stress. Current Biology. 2017;27(24):3846-3851.e3. doi:10.1016/j.cub.2017.10.063<br><br>31. Lorenzo Fabrizi, Rebeccah Slater, Alan Worley, Judith Meek, Sofia Olhede, Stewart Boyd, Maria Fitzgerald. P14-24 Development of a cortical electrophysiological response to noxious stimulation in human infants. Clinical Neurophysiology. 2010;121:S190. doi:10.1016/S1388-2457(10)60782-1<br><br>36. Madeleine Verriotis, Laura Jones, Kimberley Whitehead, Maria Laudiano-Dray, Ismini Panayotidis, Hemani Patel, Judith Meek, Lorenzo Fabrizi, Maria Fitzgerald. The distribution of pain activity across the human neonatal brain is sex dependent. NeuroImage. 2018;178:69-77. doi:10.1016/j.neuroimage.2018.05.030
**18. Madeleine Verriotis**	34. Madeleine Verriotis, Lorenzo Fabrizi, Amy Lee, Sheryl Ledwidge, Judith Meek, Maria Fitzgerald. Cortical activity evoked by inoculation needle prick in infants up to one-year old. Pain. 2015;156(2):222-230. doi:10.1097/01.j.pain.0000460302.56325.0c
**19. Marsha Campbell-Yeo**	11. Marsha Campbell-Yeo, Britney Benoit, Aaron Newman, Celeste Johnston, Tim Bardouille, Bonnie Stevens, Arlene Jiang. The influence of skin-to-skin contact on Cortical Activity during Painful procedures in preterm infants in the neonatal intensive care unit (iCAP mini): study protocol for a randomized control trial. Trials. 2022;23(1):512. doi:10.1186/s13063-022-06424-4
**20. Mohammad Reza Daliri**	51. Reyhane Shafiee, Mohammad Reza Daliri. Decoding of pain during heel lancing in human neonates with EEG signal and machine learning approach. Sci Rep. 2024;14(1):31244. doi:10.1038/s41598-024-82631-0

**21. Nathalie Maitre**	27. Lance Relland, Caitlin Kjeldsen, Arnaud Jeanvoine, Lelia Emery, Kathleen Adderley, Rachelle Srinivas, Maeve McLoughlin, Nathalie Maitre. Vibration-based mitigation of noxious-evoked responses to skin puncture in neonates and infants: a randomised controlled trial. Archives of Disease in Childhood - Fetal and Neonatal Edition. Published online March 13, 2024. doi:10.1136/archdischild-2023-326588<br><br>43. Nathalie Maitre, Ann Stark, Carrie McCoy Menser, Olena Chorna, Daniel France, Alexandra Key, Ken Wilkens, Melissa Moore-Clingenpeel, Don Wilkes, Stephen Bruehl. Cry presence and amplitude do not reflect cortical processing of painful stimuli in newborns with distinct responses to touch or cold. Arch Dis Child Fetal Neonatal Ed. 2017;102(5):F428-F433. doi:10.1136/archdischild-2016-312279
**22. Paul Castillo**	47. Paul Castillo, Sampsa Vanhatalo, Marit Lundblad, Mats Blennow, Per-Arne Lonnqvist. EEG response to a high volume (1.5 mL/kg) caudal block in infants less than 3 months. Reg Anesth Pain Med. 2023;49(3):163-167. doi:10.1136/rapm-2023-104452
**23. Pierre Kuhn**	4. Pierre Kuhn. Multimodal Approach to the Ontogenesis of Nociception in Very Preterm and Term Infants (NOCI-Prem).; 2019. https://clinicaltrials.gov/study/NCT05404594
**24. Rebecca Pillai Riddell**	5. Rebecca Pillai Riddell. Rebooting Infant Pain Assessment: Using Machine Learning to Exponentially Improve Neonatal Intensive Care Unit Practice (BabyAI).; 2020. https://clinicaltrials.gov/study/NCT05579496
**25. Rebeccah Slater**	8. Alan Worley, Lorenzo Fabrizi, Stewart Boyd, Rebeccah Slater. Multi-modal pain measurements in infants. J Neurosci Methods. 2012;205(2):252-257. doi:10.1016/j.jneumeth.2012.01.009<br><br>10. Lorenzo Fabrizi, Alan Worley, Debbie Patten, Siân Roberts-Holdridge, Laura Cornelissen, Judith Meek, Stewart Boyd, Rebeccah Slater. Electrophysiological Measurements and Analysis of Nociception in Human Infants. JoVE (Journal of Visualized Experiments). 2011;(58):e3118. doi:10.3791/3118<br><br>13. Annalisa Hauck, Marianne van der Vaart, Eleri Adams, Luke Baxter, Aomesh Bhatt, Daniel Crankshaw, Amraj Dhami, Ria Evans Fry, Marina Freire, Caroline Hartley, Roshni Mansfield, Simon Marchant, Vaneesha Monk, Fiona Moultrie, Mariska Peck, Shellie Robinson, Jean Yong, Ravi Poorun, Maria Cobo, Rebeccah Slater. Effect of parental touch on relieving acute procedural pain in neonates and parental anxiety (Petal): a multicentre, randomised controlled trial in the UK. The Lancet Child & Adolescent Health. 2024;8(4):259-269. doi:10.1016/S2352-4642(23)00340-1<br><br>16. Caroline Hartley, Fiona Moultrie, Deniz Gursul, Amy Hoskin, Eleri Adams, Richard Rogers, Rebeccah Slater. Changing Balance of Spinal Cord Excitability and Nociceptive Brain Activity in Early Human Development. Current Biology. 2016;26(15):1998-2002. doi:10.1016/j.cub.2016.05.054<br><br>17. Caroline Hartley, Eugene Duff, Gabrielle Green, Gabriela Schmidt Mellado, Alan Worley, Richard Rogers, Rebeccah Slater. Nociceptive brain activity as a measure of analgesic efficacy in infants. Science Translational Medicine. 2017;9(388):eaah6122. doi:10.1126/scitranslmed.aah6122

	18. Caroline Hartley, Fiona Moultrie, Amy Hoskin, Gabrielle Green, Vaneesha Monk, Jennifer Bell, Andrew King, Miranda Buckle, Marianne van der Vaart, Deniz Gursul, Sezgi Goksan, Edmund Juszczak, Jane Norman, Richard Rogers, Chetan Patel, Eleri Adams, Rebeccah Slater. Analgesic efficacy and safety of morphine in the Procedural Pain in Premature Infants (Poppi) study: randomised placebo-controlled trial. The Lancet. 2018;392(10164):2595-2605. doi:10.1016/S0140-6736(18)31813-0<br><br>21. Deniz Gursul, Sezgi Goksan, Caroline Hartley, Gabriela Schmidt Mellado, Fiona Moultrie, Amy Hoskin, Eleri Adams, Gareth Hathway, Susannah Walker, Francis McGlone, Rebeccah Slater. Stroking modulates noxious-evoked brain activity in human infants. Current Biology. 2018;28(24):R1380-R1381. doi:10.1016/j.cub.2018.11.014<br><br>24. Gabrielle Green, Caroline Hartley, Amy Hoskin, Eugene Duff, Adam Shriver, Dominic Wilkinson, Eleri Adams, Richard Rogers, Fiona Moultrie, Rebeccah Slater. Behavioural discrimination of noxious stimuli in infants is dependent on brain maturation. PAIN. 2019;160(2):493. doi:10.1097/j.pain.0000000000001425<br><br>37. Maria Cobo, Caroline Hartley, Deniz Gursul, Foteini Andritsou, Marianne van der Vaart, Gabriela Schmidt Mellado, Luke Baxter, Eugene Duff, Miranda Buckle, Ria Evans Fry, Gabrielle Green, Amy Hoskin, Richard Rogers, Eleri Adams, Fiona Moultrie, Rebeccah Slater. Quantifying noxious-evoked baseline sensitivity in neonates to optimise analgesic trials. Ploner M, Büchel C, Tibboel D, Ploner M, eds. eLife. 2021;10:e65266. doi:10.7554/eLife.65266<br><br>38. Maria Cobo, Gabrielle Green, Foteini Andritsou, Luke Baxter, Ria Evans Fry, Annika Grabbe, Deniz Gursul, Amy Hoskin, Gabriela Schmidt Mellado, Marianne van der Vaart, Eleri Adams, Aomesh Bhatt, Franziska Denk, Caroline Hartley, Rebeccah Slater. Early life inflammation is associated with spinal cord excitability and nociceptive sensitivity in human infants. Nat Commun. 2022;13(1):3943. doi:10.1038/s41467-022-31505-y<br><br>41. Marianne van der Vaart, Caroline Hartley, Luke Baxter, Gabriela Schmidt Mellado, Foteini Andritsou, Maria Cobo, Ria Evans Fry, Eleri Adams, Sean Fitzgibbon, Rebeccah Slater. Premature infants display discriminable behavioral, physiological, and brain responses to noxious and nonnoxious stimuli. Cerebral Cortex. 2022;32(17):3799-3815. doi:10.1093/cercor/bhab449<br><br>48. Rebeccah Slater, Alan Worley, Lorenzo Fabrizi, Siân Roberts-Holdridge, Judith Meek, Stewart Boyd, Maria Fitzgerald. Evoked potentials generated by noxious stimulation in the human infant brain. European Journal of Pain. 2010;14(3):321-326. doi:10.1016/j.ejpain.2009.05.005<br><br>49. Rebeccah Slater, Lorenzo Fabrizi, Alan Worley, Judith Meek, Stewart Boyd, Maria Fitzgerald. Premature infants display increased noxious-evoked neuronal activity in the brain compared to healthy age-matched term-born infants. NeuroImage. 2010;52(2):583-589. doi:10.1016/j.neuroimage.2010.04.253<br><br>50. Rebeccah Slater, Laura Cornelissen, Lorenzo Fabrizi, Debbie Patten, Jan Yoxen, Alan Worley, Stewart Boyd, Judith Meek, Maria Fitzgerald. Oral sucrose as an analgesic drug for procedural pain in newborn infants: a randomised controlled trial. The Lancet. 2010;376(9748):1225-1232. doi:10.1016/S0140-6736(10)61303-7

**26. Roshni Mansfield**	39. Marianne Aspbury, Roshni Mansfield, Luke Baxter, Aomesh Bhatt, Maria Cobo, Sean Fitzgibbon, Caroline Hartley, Annalisa Hauck, Simon Marchant, Vaneesha Monk, Kirubin Pillay, Ravi Poorun, Marianne van der Vaart, Rebeccah Slater. Establishing a standardised approach for the measurement of neonatal noxious-evoked brain activity in response to an acute somatic nociceptive heel lance stimulus. Cortex. 2024;179:215-234. doi:10.1016/j.cortex.2024.05.023
**27. Safa Talebi**	52. Safa Talebi, Javad Frounchi, Behzad Mozaffari Tazehkand. A novel channel selection approach for human neonate's pain EEG data analysis. SIViP. 2025;19(5):364. doi:10.1007/s11760-025-03934-x
**28. Simon Marchant**	54. Simon Marchant, Marianne van der Vaart, Kirubin Pillay, Luke Baxter, Aomesh Bhatt, Sean Fitzgibbon, Caroline Hartley, Rebeccah Slater. A machine learning artefact detection method for single-channel infant event-related potential studies. J Neural Eng. 2024;21(4):046021. doi:10.1088/1741-2552/ad5c04
**29. Sofie Nilsson**	55. Sofie Nilsson, Anton Tokariev, Timo Vehviläinen, Vineta Fellman, Sampsa Vanhatalo, Elisabeth Norman. Depression of cortical neuronal activity after a low-dose fentanyl in preterm infants. Acta Paediatrica. 2025;114(1):109-115. doi:10.1111/apa.17411
**30. Sonya Wang**	6. Sonya Wang. Effects of Music Based Intervention (MBI) on Pain Response and Neurodevelopment in Preterm Infants.; 2020. https://clinicaltrials.gov/study/NCT04286269
**31. Vineta Fellman**	7. Vineta Fellman. NeoFent-I Study; Fentanyl Treatment in Newborn Infants; a Pharmacokinetic, Pharmacodynamic and Pharmacogenetic Study.; 2012. https://clinicaltrials.gov/study/NCT03897452

Data extraction

Behnood Gholami & Kanwaljeet Anand (n=1 records)

26. Jean-Michel Roué, Amir Avnit, Behnood Gholami, Wassim Haddad, Kanwaljeet J. S. Anand. Objective Detection of Newborn Infant Acute Procedural Pain Using EEG and Machine Learning Algorithms. Paediatric and Neonatal Pain. 2025;7(1):e70001. doi:10.1002/pne2.70001

Variable	Data from publication	Data summary for review
**publication_year**		2025
**data_country**	*"The study patients were recruited from October 2017 to October 2019 in the neonatal units and maternity ward at Lucile Packard Children's Hospital at **Stanford, CA**."*	United States of America
**sample_size**	*"EEG recordings of **30** newborn infants (18 males), aged 34 to 41.7 weeks at birth, all performed under real-life, bedside conditions with the newborns in their cot were included in the analysis"*	30
**pma_birth_avg**	Table 1: *"Gestational age at birth (GA), weeks **39** (37.7–39.8) Min 34.0; Max 41.7"*	39
**pma_study_avg**	Table 1: *"Postnatal GA, weeks **39.2** (39.3–39.9) Min 34.8; Max 41.8"*	39.2
**sex_male_pct**	Table 1: *"Female, n (%) 12 (40)"* Male percentage = 100% - 40% = 60%	60
**sex_female_pct**	Table 1: *"Female, n (%) 12 (**40**)"*	40
**pain_procedure**	*"The procedures were all **heel sticks** except for one patient in the testing dataset who was recorded during an **intramuscular injection (vaccine)**"*	Heel lance, Immunization
**analgesic_intervention**	"*Swaddling, non-nutritive sucking, with or without sucrose based on clinical unit protocols*"	
**electrode_placement_method**	*"An elastic geodesic **cap** with EEG leads was set up and placed on the infant's head before the clinically indicated painful procedure."*	Cap
**electrode_placement_system**	*"An elastic **geodesic** cap with EEG leads was set up and placed on the infant's head before the clinically indicated painful procedure... EEG was recorded via a **32-channel** actiCAP snap cap, actiCAP slim electrodes"*	32-channel Geodesic system
**electrode_positions**	*"The **FCz** electrode was used as a common reference, and the ground electrode was placed at the Fpz position"* Table 5: "Electrode pair" column.	FCz, T7, P4, Fz, CP5, FC1, TP10, CP6, Cz, F3, F8, P7, P8, FT10, TP9, P3, Fp1, O2, FC6
**eeg_data_loss_pct**	Not applicable	
**epoch_rej_method**	Epochs were not corrected, not rejected. *"**Automated IC rejection** was performed using the multiple artifact rejection algorithm (MARA) [44–46], evaluating each component*	

	on six features, based on temporal, spectral, and spatial information. ICs were rejected in cases where the probability that the signal is dominated by an artifact signal was > 50%. ***Rejected channels were imputed*** *using spherical interpolation with Legendre polynomials up to the 7th order."*	
**amplitude_threshold**	Not applicable	
**clinical_pain_scale**	*"Validated pain scores (**PIPP-- R** and **NFCS**) were assessed at the time of the painful procedure"*	PIPP/PIPP-R, NFCS
**non_eeg_recording**	Table 1	Heart rate, Oxygen saturation

Britney Benoit (n=1 records)

14. Britney Benoit, Aaron Newman, Ruth Martin-Misener, Margot Latimer, Marsha Campbell-Yeo. The influence of breastfeeding on cortical and bio-behavioural indicators of procedural pain in newborns: Findings of a randomized controlled trial. Early Human Development. 2021;154:105308. doi:10.1016/j.earlhumdev.2021.105308

Variable	***Data from publication***	***Data summary for review***
**publication_year**	2021	2021
**data_country**	"A single-blind, randomized controlled trial was completed on the Family Newborn Care Unit of the IWK Health Centre in Halifax, Nova Scotia, **Canada**..."	Canada
**sample_size**	Fig.2: number of babies that "*received allocated intervention*": 18+19=37	37
**pma_birth_avg**	Table 1: "*Gestational age (GA) in weeks, M(SD)*": (39.3+39.5)/2=39.4	39.4
**pma_study_avg**	Table 1: "*Postnatal age at heel lance in days, M(SD)*": (1+1)/2=1 day= 1/7 week= 0.14week. 0.14+39.4 week= 39.54 week	39.5
**sex_male_pct**	Table 1: "Male sex (frequency, %)": (60+57.9)/2= 58.95	58.95
**sex_female_pct**	100-58.95= 41.05	41.05
**pain_procedure**	*"39 full-term infants were randomized to receive breastfeeding or 0.24 mL of 24% oral sucrose plus offered non-nutritive sucking 2 min prior to* ***heel lance****."*	Heel lance
**analgesic_intervention**	*"Participating infants were randomized to one of two possible interventions during a clinically-required heel lance: 1) direct* ***breastfeeding*** *or 2)* ***24% oral sucrose*** *combined with offered non-nutritive sucking and containment in a blanket while in an infant cot."*	Breastfeeding, Sucrose
**electrode_placement_method**	Assumed to be 'cap' because "*32-channel Geodesic EEG System"* was used. *"The primary outcome was pain-related brain activity measured use neonatal electroencephalogram (****32-channel Geodesic EEG System*** *TM 400 series, Electrical Geodesic Inc. [EGI], Eugene, OR, USA)..."*	Cap
**electrode_placement_system**	*"The primary outcome was pain-related brain activity measured use neonatal electroencephalogram (****32-channel Geodesic EEG System*** *TM 400 series, Electrical Geodesic Inc. [EGI], Eugene, OR, USA)..."*	32-channel Geodesic system
**electrode_positions**	*"Pain-related event-related potential was specifically examined and isolated at vertex electrode E19 (EGI Hydrocel Geodesic Sensor Net, corresponding with electrode* ***Pz*** *of the international 10/10 electrode placement..."*	Pz

**eeg_data_loss_pct**	Fig.2: "*Movement artifact (**n=4**)"* + "*Movement artifact (**n=5**)"* = 9. 9/37*100=24.32%	24.32
**epoch_rej_method**	*"Individual infants were excluded if there was movement artifact detected (voltage change > than 100 microV over 50-milliseconds) in the 1500-millisecond epochs corresponding to stimuli and artifact correction was completed using independent component analysis."*	Objective
**amplitude_threshold**	*"Individual infants were excluded if there was movement artifact detected (**voltage change > than 100 microV over 50-milliseconds**) in the 1500-millisecond epochs corresponding to stimuli and artifact correction was completed using independent component analysis."*	Voltage change > 100 microV over 50-milliseconds
**clinical_pain_scale**	*"Secondary outcomes included Premature Infant **Pain Profile – Revised (PIPP-R)** score,..."*	PIPP/PIPP-R
**non_eeg_recording**	"The measurement of the main study outcomes relied on five data collection strategies: Continuous neonatal EEG recording, close-up **video recording of infant facial actions**, **pulse oximeter monitoring of heart rate and oxygen saturation**, observation of the data collection session for adverse events, and a chart review."	Oxygen saturation, Heart rate, Video recording of facial expression, Video recording of sleep and behavioral state

Caroline Hartley (n=4 records)

9. Alan Worley, Kirubin Pillay, Maria Cobo, Gabriela Schmidt Mellado; Marianne van der Vaart; Aomesh Bhatt, Caroline Hartley. The PiNe box: Development and validation of an electronic device to time-lock multimodal responses to sensory stimuli in hospitalised infants. PLOS ONE. 2023;18(7):e0288488. doi:10.1371/journal.pone.0288488

Variable	*Data from publication*	*Data summary for review*
**publication_year**	2023	2023
**data_country**	*"The infants were recruited from the Newborn Care Unit and Maternity wards of the John Radcliffe Hospital (Oxford University Hospitals NHS Foundation Trust, Oxford,* ***United Kingdom****)."*	United Kingdom

15. Caroline Hartley, Sezgi Goksan, Ravi Poorun, Kelly Brotherhood, Gabriela Schmidt Mellado, Fiona Moultrie, Richard Rogers, Eleri Adams, Rebeccah Slater. The relationship between nociceptive brain activity, spinal reflex withdrawal and behaviour in newborn infants. Sci Rep. 2015;5(1):12519. doi:10.1038/srep12519

Variable	*Data from publication*	*Data summary for review*
**publication_year**	2015	2015
**data_country**	*"30 infants were recruited from the Maternity Unit and Special Care Baby Unit at the* ***John Radcliffe Hospital*** *between May 2012 and January 2015."*	United Kingdom
**sample_size**	*"Heel lancing was performed as part of the infants' routine clinical care (n =* ***10****)"*	10
**pma_birth_avg**	Not provided	
**pma_study_avg**	Not provided	
**sex_male_pct**	Not provided	
**sex_female_pct**	Not provided	
**pain_procedure**	*"****Heel lancing*** *was performed as part of the infants' routine clinical care (n = 10)"*	Heel lance
**analgesic_intervention**	Not applicable	
**electrode_placement_method**	Electrode positions were listed and 'cap' was not mentioned, so we assume individual electrodes placement method were used.	Individual electrodes
**electrode_placement_system**	*"EEG was recorded at eight scalp electrodes (Ambu Neuroline disposable Ag/AgCl cup electrodes) in positions Cz, CPz, C3, C4, FCz, Oz, T3 and T4 according to* ***the modified international 10–20 system****."*	Modified 10-20 system
**electrode_positions**	*"EEG was recorded at eight scalp electrodes (Ambu Neuroline disposable Ag/AgCl cup electrodes) in* ***positions Cz, CPz, C3, C4, FCz, Oz, T3 and T4*** *according to the modified international 10–20 system. The reference electrode was positioned at* ***Fz*** *and the ground was placed on the forehead… In 3 other studies the reference electrode was placed at* ***Fpz*** *and re-referenced*	Cz, CPz, C3, C4, FCz, Oz, T7, T8, Fz

	to Fz post-acquisition..” T3 and T4, are standardised to T7 and T8, respectively, according to the current version of the 10-10 system.	
**eeg_data_loss_pct**	The number of babies with EEG recorded during skin-breaking procedure is n=10 (*“Heel lancing was performed as part of the infants' routine clinical care (n =* ***10****)”*). Six were used to characterize the brain activity (“*The second principal component (PC), which accounted for 42.5% of the variance, was defined as nociceptive-specific because the weight of this component was significantly greater in response to the noxious heel lance, compared with non-noxious control stimulation and background EEG activity (n =* ***6****; p = 0.0026, Fig. 1).”).* Four were analysed in subsequent analyses (“*Having characterised the pattern of nociceptive-specific brain activity following clinical heel lance in a sample of infants (Fig. 1), we then determined whether this activity was evoked by the experimental noxious stimuli (n=12), and for comparison, by a clinical heel lance performed in an independent sample of infants (n=****4****).*”). Thus, no EEG data was excluded after recording due to artefact.	0
**epoch_rej_method**	*“EEG data epochs were rejected* ***if gross movement artefacts were present.****”*	Subjective
**amplitude_threshold**	Not applicable	
**clinical_pain_scale**	None on babies who received skin-breaking procedure. *“Clinical pain scores were calculated in response to experimental noxious stimuli in an independent sample of infants (n = 10) using the Premature Infant Pain Profile (PIPP)”*	
**non_eeg_recording**	*“Bipolar* ***EMG*** *electrodes (Ambu Neuroline 700 solid gel surface electrodes) were placed on the biceps femoris of both legs.”*	EMG

19. Caroline Hartley, Luke Baxter, Fiona Moultrie, Ryan Purdy, Aomesh Bhatt, Richard Rogers, Chetan Patel, Eleri Adams, Rebeccah Slater. Predicting severity of adverse cardiorespiratory effects of morphine in premature infants: a post hoc analysis of Procedural Pain in Premature Infants trial data. British Journal of Anaesthesia. 2021;126(4):e133-e135. doi:10.1016/j.bja.2020.10.034

Variable	***Data from publication***	***Data summary for review***
**publication_year**	2021	2021
**data_country**	*“We conducted a post hoc analysis of Poppi trial data…Full*	United Kingdom

	details of recruitment, original trial design, and procedures are given elsewhere." As directed from the paper, relevant information was extracted from the reference cited (http://dx.doi.org/10.1016/S0140-6736(18)31813-0). *"In this single-centre masked trial, 31 infants at the John Radcliffe Hospital, Oxford,* ***UK****, were randomly allocated using a web-based facility with a minimisation algorithm to either 100 µg/kg oral morphine sulphate or placebo 1 h before a clinically required heel lance and retinopathy of prematurity screening examination, on the same occasion."*	
**sample_size**	*"**Fifteen** infants in the trial received oral morphine (100 mg kg 1) ~1 h before the clinical procedure."*	15
**pma_birth_avg**	*"We conducted a post hoc analysis of Poppi trial data…Full details of recruitment, original trial design, and procedures are given elsewhere."* As directed from the paper, relevant information was extracted from the reference cited (http://dx.doi.org/10.1016/S0140-6736(18)31813-0): Table 1: *"Gestational age (weeks)*† **28·1** (26·3–30·1)"*	28.1
**pma_study_avg**	*"We conducted a post hoc analysis of Poppi trial data…Full details of recruitment, original trial design, and procedures are given elsewhere."* As directed from the paper, relevant information was extracted from the reference cited (http://dx.doi.org/10.1016/S0140-6736(18)31813-0): Table 1: *"Gestational age (weeks)*† **34·7** (34·1–35·1)"*	34.7
**sex_male_pct**	*"We conducted a post hoc analysis of Poppi trial data…Full details of recruitment, original trial design, and procedures are given elsewhere."* As directed from the paper, relevant information was extracted from the reference cited (http://dx.doi.org/10.1016/S0140-6736(18)31813-0): Table 1: *"Male sex 12 (**80%**)"*	80
**sex_female_pct**	*"We conducted a post hoc analysis of Poppi trial data…Full details of recruitment, original trial design, and procedures are given elsewhere."* As directed from the paper, relevant information was extracted from the reference cited (http://dx.doi.org/10.1016/	20

	S0140-6736(18)31813-0): Table 1: *"Female sex 3 (**20%**)"*	
**pain_procedure**	*"The magnitude of the noxious-evoked brain activity after **heel lancing** was significantly lower in infants who received treatment for respiratory adverse effects than in infants who did not receive treatment"*	Heel lance
**analgesic_intervention**	*"Fifteen infants in the trial received oral **morphine** (100 mg kg 1) ~1 h before the clinical procedure."*	Morphine
**electrode_placement_method**	*"We conducted a post hoc analysis of Poppi trial data…Full details of recruitment, original trial design, and procedures are given elsewhere."* As directed from the paper, relevant information was extracted from the reference cited (http://dx.doi.org/10.1016/ S0140-6736(18)31813-0): Electrode positions were listed and 'cap' was not mentioned, so we assume individual electrodes placement method were used.	Individual electrodes
**electrode_placement_system**	*"We conducted a post hoc analysis of Poppi trial data…Full details of recruitment, original trial design, and procedures are given elsewhere."* As directed from the paper, relevant information was extracted from the reference cited (http://dx.doi.org/10.1016/ S0140-6736(18)31813-0): *"Eight EEG recording electrodes (Ambu Neuroline disposable Ag/AgCl cup electrodes) were positioned on the scalp at Cz, CPz, C3, C4, FCz, T3, T4 and Oz, according to **the modified international 10-20 System**, with reference and ground electrodes at Fz and the forehead respectively."*	Modified 10-20 system
**electrode_positions**	*"We conducted a post hoc analysis of Poppi trial data…Full details of recruitment, original trial design, and procedures are given elsewhere."* As directed from the paper, relevant information was extracted from the reference cited (http://dx.doi.org/10.1016/ S0140-6736(18)31813-0): *"Eight EEG recording electrodes (Ambu Neuroline disposable Ag/AgCl cup electrodes) were positioned on the scalp at **Cz, CPz, C3, C4, FCz, T3, T4 and Oz**, according to the modified international 10-20 System, with reference and ground electrodes at **Fz** and the forehead respectively."* T3 and T4 are standardised to T7 and T8,	Cz, CPz, C3, C4, FCz, T7, T8, Oz, Fz

	respectively, according to the current version of the 10-10 system.	
**eeg_data_loss_pct**	*"We conducted a post hoc analysis of Poppi trial data…Full details of recruitment, original trial design, and procedures are given elsewhere."* As directed from the paper, relevant information was extracted from the reference cited (http://dx.doi.org/10.1016/S0140-6736(18)31813-0): Figure 1: n=15 babies assigned morphine and n=15 babies included in EEG analysis, so percentage of data loss = 0.	0
**epoch_rej_method**	Not provided	
**amplitude_threshold**	Not applicable	
**clinical_pain_scale**	*"However, there was no significant difference between the two groups in **the Premature Infant Pain Profile-Revised score** (a composite behavioural and physiological pain score) after ROP screening … or heel lancing…"*	PIPP/PIPP-R
**non_eeg_recording**	*"We conducted a post hoc analysis of Poppi trial data…Full details of recruitment, original trial design, and procedures are given elsewhere."* As directed from the paper, relevant information was extracted from the reference cited (http://dx.doi.org/10.1016/S0140-6736(18)31813-0). *"Continuous electronic data capture of **heart rate, respiratory rate**, and **oxygen saturation** began approximately 24 h before the clinical procedure to establish a baseline of clinical stability for every infant. We recorded all changes in oxygen requirement, and measured **blood pressure** every 6 h. …Electroencephalography (EEG) and electromyography (**EMG**) electrodes were then sited, as described in the appendix…Shortly before the clinical procedure, we swaddled the infant (to provide non-pharmacological pain relief), began **video monitoring**…"* Supplementary appendix: "***Facial expressions were filmed throughout the clinical procedure**; a clear view of the face was recorded for 15 seconds before and 30 seconds after the heel lance control, the heel lance and the ROP screening."*	EMG, Heart rate, Respiratory rate, Oxygen saturation, Video recording of facial expression, Blood pressure

40. Marianne van der Vaart, Eugene Duff, Nader Raafat, Richard Rogers, Caroline Hartley, Rebeccah Slater. Multimodal pain assessment improves discrimination between noxious and non-noxious stimuli in infants. Paediatric and Neonatal Pain. 2019;1(1):21-30. doi:10.1002/pne2.12007

Variable	***Data from publication***	***Data summary for review***
**publication_year**	2019	2019
**data_country**	*"Infants were recruited between 2012 and 2017 from the Maternity Ward and the Neonatal Unit at the John Radcliffe Hospital, Oxford University Hospitals NHS Foundation Trust,* ***UK****."*	United Kingdom
**sample_size**	Figure 1: "EEG N=47...All features N= 32" Total sample size = 47+32 = 79	79
**pma_birth_avg**	Table 1: *"Gestational age at birth (wk)* ***37.0*** *(32.2-40.0)* ***39.7*** *(37.1-40.7)"* This table presented information from all babies recruited, so including babies who did not have EEG recording. Average PMA at birth = (37+39.7)/2 = 38.35	38.35
**pma_study_avg**	Table 1: *"Gestational age at study (wk)* ***38.9*** *(36.6-40.3)* ***40.2*** *(37.6-41.1)"* This table presented information from all babies recruited, so including babies who did not have EEG recording. Average PMA at study = (38.9+40.2)/2 = 39.55	39.55
**sex_male_pct**	Table 1: *"Sex Male 40 (****52****) 16 (****50****)"* This table presented information from all babies recruited, so including babies who did not have EEG recording. Average percentage of males = (52+50)/2 = 51	51
**sex_female_pct**	Table 1: *"Female 37 (****48****) 16 (****50****)"* This table presented information from all babies recruited, so including babies who did not have EEG recording. Average percentage of females = (48+50)/2 = 49	49
**pain_procedure**	*"In 109 infants who received a clinically required* ***heel lance*** *and a control non-noxious stimulus,..."*	Heel lance
**analgesic_intervention**	Not applicable	
**electrode_placement_method**	Electrode positions were listed and 'cap' was not mentioned, so we assume individual electrodes placement method were used.	Individual electrodes
**electrode_placement_system**	*"EEG was recorded using Ambu Neuroline disposable Ag/AgCl cup electrodes at Cz, CPz, C3, C4, FCz, Oz, T3, and T4 according to* ***the modified interna-tional 10-20 system****, with a reference electrode at Fz and a ground electrode on the forehead."*	Modified 10-20 system
**electrode_positions**	*"EEG was recorded*	Cz, CPz, C3, C4, FCz, Oz, T7, T8, Fz

	using Ambu Neuroline disposable Ag/AgCl cup electrodes at ***Cz, CPz, C3, C4, FCz, Oz, T3, and T4*** *according to the modified international 10-20 system, with a reference electrode at* ***Fz*** *and a ground electrode on the forehead."* T3 and T4 are standardised to T7 and T8, respectively, according to the current version of the 10-10 system.	
**eeg_data_loss_pct**	The number of babies with that were excluded due to artifact is not specified in the paper, only the total number of traces rejected was reported. "*Of the 168 available EEG traces, 16 were rejected due to movement artifacts.*"	
**epoch_rej_method**	Not provided	
**amplitude_threshold**	Not applicable	
**clinical_pain_scale**	Not applicable	
**non_eeg_recording**	*"Infants'* ***facial responses were recorded*** *with a handheld camera...* ***Oxygen saturation was measured with a pulse oximeter*** *placed on the infant's foot... ECG traces were preprocessed by extracting RR intervals as described previously.30 The* ***heart rate*** *in beats per minutes (bpm) was calculated at each second according to the mean RR interval in the previous 5 seconds... Bipolar* ***EMG*** *electrodes (Ambu Neuroline 700 solid gel surface electrodes) on the biceps femoris of both legs were used to measure reflexes."*	Video recording of facial expression, EMG, Heart rate, Oxygen saturation

Caterina Coviello (n=1 records)

20. Caterina Coviello, Silvia Lori, Giovanna Bertini, Simona Montano, Simonetta Gabbanini, Maria Bastianelli, Cesarina Cossu, Sara Cavaliere, Clara Lunardi, Carlo Dani. Evaluation of the Relationship between Pain Exposure and Somatosensory Evoked Potentials in Preterm Infants: A Prospective Cohort Study. Children. 2024;11(6):676. doi:10.3390/children11060676

Variable	Data from publication	Data summary for review
**publication_year**		2024
**data_country**	*"Preterm neonates born <32 weeks GA, admitted to the NICU of the Careggi University Hospital of* ***Florence*** *between September 2018 and May 2021, were recruited for this prospective cohort study... The healthy infants cohort was a historical control group enrolled at the Careggi University Hospital between January 2013 and December 2013."*	Italy
**sample_size**	Table 1: *"n =* ***86****"*	86
**pma_birth_avg**	Table 1: "*Gestational age (wks)* ***27.9*** *± 1.9*"	27.9
**pma_study_avg**	Table 1: *"PMA at SEP recording (wks)* ***39.3*** *± 1.2"*	39.3
**sex_male_pct**	*"Male 49 (****57****)"*	57
**sex_female_pct**	*"Male 49 (57)"* Female percentage = 100-57 = 43%	43
**pain_procedure**	*"The exposure to neonatal invasive procedures was quantified by recording the number of skin-breaking procedures (****heel lance****, endotracheal intubation,* ***peripheral intravenous or central line insertion****,* ***intra-muscular injection****,* ***chest tube*** *and urinary catheter insertion,* ***lumbar puncture****) during the first 4 weeks after birth."* Peripheral intravenous line insertion was standardized to venipuncture.	Heel lance, Venipuncture, Central line insertion, Intra-muscular injection, Chest tube, Lumbar puncture
**analgesic_intervention**	Not applicable	
**electrode_placement_method**	Not provided	Individual electrodes
**electrode_placement_system**	Not provided	10-20 system
**electrode_positions**	*"Continuous SEPs were simultaneously obtained with video EEG (VEEG) from the same pool of electrodes...The responses were recorded from the cortical (N1 and P1) and cervical (N13) levels and the reference electrode at Fz:* ***C3****'-Fz, C3'-****C4****', C4'-****Fz****, C4'-C3', and Cv7-Fz."* To standardize with the 10-20 system, for the EEG C3 was extracted as C3' as the location is close to C3, C4 was extracted as C4' as the location is close to C4.	C3', C4', Fz and Cv7
**eeg_data_loss_pct**	Not provided	

**epoch_rej_method**	Not applicable. No epoch rejection method was mentioned in the paper.	
**amplitude_threshold**	Not applicable. No epoch rejection method was mentioned in the paper.	
**clinical_pain_scale**	*"**The Neonatal Pain Agitation and Sedation Scale** was used for acute pain assessment"*	N-PASS
**non_eeg_recording**	*"Nemus-EB Neuro polygraph and GalNT/EP EXAM software (http://www.ebneuro. com/en/emg/nemus-1, 1 April 2024) were used for **SEP recordings**."*	SEP

Eilon Shany (n=1 records)

44. Neta Maimon, Ruth Grunau, Ivan Cepeda, Michael Friger, Leonel Selnovik, Shlomo Gilat, Eilon Shany. Electroencephalographic Activity in Response to Procedural Pain in Preterm Infants Born at 28 and 33 Weeks Gestational Age. The Clinical Journal of Pain. 2013;29(12):1044. doi:10.1097/AJP.0b013e318284e525

Variable	*Data from publication*	*Data summary for review*
**publication_year**	2013	2013
**data_country**	*"The study was a prospective cohort trial conducted in the NICU at the Soroka Medical Centre, Beer Sheva, **Israel**."*	Israel
**sample_size**	Figure 2: "*Group 1 (First Test, 30 Infants)","Group 3 (35 Infants)":* 30+35= 65. "Study protocol not followed" is included in sample size because assumed to still have EEG data during skin-breaking procedures.	65
**pma_birth_avg**	Table 1: "*GA at birth (wk)*" for Group 1 and Group 3 (Group 2 is not included because they are the second test of Group 1): (28.9+33)/2= 30.95	30.95
**pma_study_avg**	Table 1: "*PCA at time of test (wk)"* for Group 1 and Group 3 (Group 2 is not included because they are the second test of Group 1): (30+34.1)/2= 32.05	32.05
**sex_male_pct**	Table 1: "*Boys, n (%) 14 (**58**) 12 (54) 15 (**60**)".* Only Group 1 and Group 3 are included when averaging, because Group 2 is the second test of Group 1. (58+60)/2= 59	59
**sex_female_pct**	100-59= 41	41
**pain_procedure**	*"Our aim was to examine whether **heel lance** for blood collection induces changes in right-left frontal asymmetry,..."*	Heel lance
**analgesic_intervention**	Not applicable	
**electrode_placement_method**	Electrode positions were listed and 'cap' was not mentioned, so we assume individual electrodes placement method were used.	Individual electrodes
**electrode_placement_system**	*"...activity was recorded from midfrontal (Fp1,Fp2), central (C3, C4), and vertex (Pz) areas (according to **the international 10 to 20 placement system**) and referenced to Pz."*	10-20 system
**electrode_positions**	*"...activity was recorded from midfrontal (**Fp1, Fp2**), central (**C3, C4**), and vertex (**Pz**) areas (according to the international 10 to 20 placement system) and referenced to Pz."*	Fp1, Fp2, C3, C4, Pz
**eeg_data_loss_pct**	Fig.2. No EEG data was mentioned to be excluded due to artefact.	0
**epoch_rej_method**	*"The first 30-second period free of movements and electrical induction*	Subjective

	artifact detected before and after the hill prick and 10 minutes following the hill prick were chosen by one of the investigators (N.M.) and confirmed by a second investigator (E.S.) who is experienced with neonatal EEG interpretation.”	
**amplitude_threshold**	Not applicable	
**clinical_pain_scale**	*“**The Behavioral Indicators of Infant Pain (BIIP)** scored at bedside was used for data analysis...”*	BIIP
**non_eeg_recording**	*“Continuous EEG, video recording, and bedside behavior scoring were carried out during a blood collection by heel lance performed for clinical purposes... the video camera was set up for a close-up view of the **face and body**... **Video recording** was carried out as back-up for BIIP scoring”*	Video recording of facial expression, Video recording of movement

Elisabeth Norman (n=3 records)

1. Elisabeth Norman. Fentanyl and Clonidine for Analgesia During Hypothermia in Term Asphyxiated Infants (SANNI 1).; 2017. https://clinicaltrials.gov/study/NCT03177980

Variable	Data from publication	Data summary for review
**publication_year**	*"Study Start (Actual)<br>**2017**-04-24"*	2017
**data_country**	*"This study has 2 locations<br>**Sweden**<br>Lund, Sweden, 221 85<br><br>Skåne Uniersity Hospital<br>Stockholm, Sweden, 171 76<br><br>Karolinska University Hospital"*	Sweden

2. Elisabeth Norman. Clonidine for Analgesia to Preterm Infants During Neonatal Intensive Care.; 2018. https://clinicaltrials.gov/study/NCT04928651

Variable	Data from publication	Data summary for review
**publication_year**	"Study Start (Actual)<br>**2018**-04-06"	2018
**data_country**	"This study has 2 locations<br>**Sweden**<br><br>Lund, Sweden, 221 85<br>Skane University Hospital<br><br><br>Stockholm, Sweden, 171 76<br>Marco Bartocci"	Sweden

22. Elisabeth Norman, Ingmar Rosen, Sampsa Vanhatalo, Karin Stjernqvist, Ove Okland, Vineta Fellman, Lena Hellstrom-Westas. Electroencephalographic Response to Procedural Pain in Healthy Term Newborn Infants. Pediatr Res. 2008;64(4):429-434. doi:10.1203/PDR.0b013e3181825487

Variable	Data from publication	Data summary for review
**publication_year**	2008	2008
**data_country**	*"Three groups of healthy term infants were investigated at **Lund University Hospital, Sweden** and **Ålesund Hospital, Norway**…In group 2a, a venous blood sample from the dorsum of the left hand was obtained from 22 infants at an age of 72 h or more at the maternity unit at **Lund University Hospital**, all by the same experienced midwife. In group 3 (n 25), capillary blood sampling was performed with an automatic lancet (Tenderfoot, Skafte Medlab AB, Onsala, Sweden) by laboratory staff at the maternity ward, **Ålesund Hospital, Norway**."*	Sweden, Norway

**sample_size**	*"Six recordings (three in group 1,* ***one in group 2, and two in group 3) were excluded because of technical artifacts****, thus in total 72 recordings (12 + 16 in group 1,* ***21 in group 2 and 23 in group 3****) were included.*"* Infants receiving skin-breaking procedures with EEG recording = 1+21 (group 2) +2+23 (group 3) = 47	47
**pma_birth_avg**	Table 1: This table reported the data of only baby who were included in the analysis, so not all babies with EEG recording. The average of group 2 and 3 who received skin-breaking procedures are calculated. "*GA, wk 40 (39 – 42) 39.5 (37– 41)* ***40*** *(38 – 41)* ***40*** *(37– 41)"* Average GA = (40+40)/2 = 40 weeks	40
**pma_study_avg**	Table 1: This table reported the data of only baby who were included in the analysis, so not all babies with EEG recording. The average of group 2 and 3 who received skin-breaking procedures are calculated. PNA in hours unit are converted to weeks, and are added up to the average GA to get PMA in weeks. *"PNA at EEG, h 67.5 (42– 84) 56.5 (37–94)* ***120*** *(96 –143)* ***70*** *(61– 81)".* Average PNA = (120+70)/2 = 95 hours = 95/24/7 = 0.57 weeks. PMA = 0.57 + 40 = 40.57 weeks.	40.57
**sex_male_pct**	Table 1: This table reported the data of only baby who were included in the analysis, so not all babies with EEG recording. The percentage of male in group 2 and 3 who received skin-breaking procedures are calculated. *"Sex (male/female) 6/7 8/7* ***12****/9* ***10****/13"* Total male = 12+10 = 22. Total group 2+group 3 in the table = 21+23 = 44. Male percentage = 22/44*100 = 50%	50
**sex_female_pct**	Table 1: This table reported the data of only baby who were included in the analysis, so not all babies with EEG recording. The percentage of female in group 2 and 3 who received skin-breaking procedures are calculated. *"Sex (male/female) 6/7 8/7 12/****9*** *10/****13****"* Total female = 9+13 = 22. Total group 2+group 3 in the table = 21+23 = 44. Female percentage = 22/44*100 = 50%	50
**pain_procedure**	"*In group 2a, a* ***venous blood sample from the dorsum of the left hand*** *was obtained from 22 infants at an age of 72 h or more at the maternity unit at Lund University Hospital, all by the same experienced midwife. In group 3 (n 25),* ***capillary blood sampling was***	Venipuncture, Heel lance

	performed with an automatic lancet *(Tenderfoot, Skafte Medlab AB, Onsala, Sweden) by laboratory staff at the maternity ward, Ålesund Hospital, Norway.*"	
**analgesic_intervention**	Not applicable (No analgesic intervention was studied in babies receiving skin-breaking procedures).	
**electrode_placement_method**	Electrode positions were listed and 'cap' was not mentioned, so we assume individual electrodes placement method were used.	Individual electrodes
**electrode_placement_system**	Only reported for group 1: *"Group 1 consisted of 31 term infants who were recruited at the maternity unit and had a full EEG (electrode positions according to the International 10 –20 system…"* Group 1 did not receive skin-breaking procedures, while group 2 and 3 received skin-breaking procedures. No electrode placement system is reported for group 2 and 3.	
**electrode_positions**	*"The EEGs in groups 2 and 3 were recorded from five derivations (**F3, F4, Cz, P3, and P4**)"*	F3, F4, Cz, P3, P4
**eeg_data_loss_pct**	*"Six recordings (three in group 1, **one** in group 2, and **two** in group 3) were **excluded because of technical artifacts**, thus in total 72 recordings (12 + 16 in group 1, 21 in group 2 and 23 in group 3) were included."* Total number of infants receiving skin-breaking procedures that were excluded = 1 (group 2) +2 (group 3) = 3. Percentage of data loss = 3/47 (see 'sample size' of this table) *100 = 6.38%	6.38
**epoch_rej_method**	*"All EEG registrations were assessed for artifacts by the first author (E.N.) and, when in doubt, also by a clinical neurophysiologist (I.R.)"*	Subjective
**amplitude_threshold**	Not applicable	
**clinical_pain_scale**	*"Behavioral pain responses were assessed with the **Premature Infant Pain Profile Scale**."*	PIPP/PIPP-R
**non_eeg_recording**	"**Oxygen saturation** (SaO2) and **heart rate** were monitored (Nellcor N395, Tyco Healthcare Norden AB, Sweden) and **videotaped**."	Oxygen saturation, Heart rate

Geraldine Boylan (n=1 records)

12. Andreea Pavel, Farah Abu Dhais, Caoimhe Howard, John O'Toole, Elena Pavlidis, Daragh Finn, Vicki Livingstone, Anna Powell, Eugene Dempsey, Geraldine Boylan. GP252 The effect of music therapy on the electroencephalogram (EEG) and heart rate variability (HRV) of premature infants during routine painful procedures. Archives of Disease in Childhood. 2019;104(Suppl 3):A135-A135. doi:10.1136/archdischild-2019-epa.311

Variable	*Data from publication*	*Data summary for review*
**publication_year**	2019	2019
**data_country**	Based on the authors' affiliations	Ireland
**sample_size**	*"**Twenty-one** preterm infants were recruited"*	21
**pma_birth_avg**	Only maximum age at birth was provided: "*This was a randomised crossover study in newborns delivered before 32 weeks gestational age (GA)"*	
**pma_study_avg**	Only maximum age at birth was provided: *"This was a randomised crossover study in newborns delivered before 32 weeks gestational age (GA)"*	
**sex_male_pct**	"*Twenty-one preterm infants were recruited 8 males (**38%**) and 13 females (62%), mean birth weight 932 g."*	38
**sex_female_pct**	*"Twenty-one preterm infants were recruited 8 males (38%) and 13 females (**62%**), mean birth weight 932 g."*	62
**pain_procedure**	*"Infants were randomised to receive initially either sucrose or sucrose and music therapy (Brahms' lullaby) during routine **venepuncture**."*	Venipuncture
**analgesic_intervention**	*"Infants were randomised to receive initially either sucrose or sucrose and **music therapy** (Brahms' lullaby) during routine venepuncture."*	Music therapy
**electrode_placement_method**	Not provided	
**electrode_placement_system**	Not provided	
**electrode_positions**	Not provided	
**eeg_data_loss_pct**	Not provided	
**epoch_rej_method**	Not provided	
**amplitude_threshold**	Not provided	
**clinical_pain_scale**	Not applicable	
**non_eeg_recording**	*"Matlab was used to perform quantitative EEG and **HRV** analysis."*	Heart rate

Guy Dumont (n=1 records)

53. Shahbaz Askari, Zoya Bastany, Liisa Holsti, Guy Dumont. Lighting up babies' brains: development of a combined NIRS/EEG system for infants. In: Biophotonics in Exercise Science, Sports Medicine, Health Monitoring Technologies, and Wearables II. Vol 11638. SPIE; 2021:80-85. doi:10.1117/12.2595899

Variable	*Data from publication*	*Data summary for review*
**publication_year**	2021	2021
**data_country**	*"...are being recruited from the NICU at* ***BCWH****"*	Canada
**sample_size**	*"****Nine*** *subjects have been recorded in this research..."*	9
**pma_birth_avg**	Not provided. Only range of age was reported: *"born between 27-35 weeks gestational age..."*	
**pma_study_avg**	Not provided. Only range of age was reported: *"born between 27-35 weeks gestational age..."*	
**sex_male_pct**	Not provided	
**sex_female_pct**	Not provided	
**pain_procedure**	*"In this experiment, we investigate the change of cerebral hemodynamic across 3 phases of blood collection, baseline,* ***heel lance****, recovery"*	Heel lance
**analgesic_intervention**	Not applicable	
**electrode_placement_method**	"...we designed and developed a prototype NIRS/EEG instrument... The **novel NIRS/EEG probe consists of Ag/AgCl EEG electrodes**..."	Other
**electrode_placement_system**	Not provided	
**electrode_positions**	Not provided	
**eeg_data_loss_pct**	Not provided	
**epoch_rej_method**	Not provided	
**amplitude_threshold**	Not provided	
**clinical_pain_scale**	Not applicable	
**non_eeg_recording**	"...The regional oxygenation (**NIRS**) and neuronal activity (EEG) are measured in the forehead area, the **behavioural responses** of subjects are **recorded in real-time using a bedside video camera** in the NICU (Neonate ICU)."	NIRS, Video recording of sleep and behavioral state

Hisham Abdelsami Awad (n=1 records)

25. Hisham Abdelsami Awad, Sahar Hassanein, Rania Mohamed Abdou, L Taher Bassiouny. Analysis of pain effect on EEG recordings and oral sucrose suckling effect on pain reduction in neonates. QJM: An International Journal of Medicine. 2018;111(suppl_1):hcy200.158. doi:10.1093/qjmed/hcy200.158

Variable	*Data from publication*	*Data summary for review*
**publication_year**	2018	2018
**data_country**	Based on authors' affiliation	Egypt
**sample_size**	*"The EEG recordings of a cohort of **21** neonates..."*	21
**pma_birth_avg**	Not provided	
**pma_study_avg**	Not provided. Only postnatal age in days unit were provided. ("*Postnatal age ranged between 3 and 27 days, with a mean of 14.05 6 7.32 days.*") Calculation of PMA at study in weeks unit is not possible because PMA at birth (gestational age) is not provided.	
**sex_male_pct**	Not provided	
**sex_female_pct**	Not provided	
**pain_procedure**	*"EEG recording, vital data and Neonatal Infant Pain Scale (NIPS) scoring were performed before and following painful stimulation via **heel stick blood sampling** during routine blood glucose measurements via glucometer, during non-nutritive suckling (NNS) and during suckling of sucrose."*	Heel lance
**analgesic_intervention**	*"This study aimed at assessing the effects of pain on the EEG picture of neonates, and whether or not, **sucrose** administration can alleviate pain associated with invasive neonatal procedures."*	Sucrose
**electrode_placement_method**	Not provided	
**electrode_placement_system**	Not provided	
**electrode_positions**	*"Results also concluded that there is a significant positive relationship between heart rate and the frequency of beta waves at electrode position **F4**... A significant correlation between heart rate and the frequency of theta EEG waves at electrode position **F3**..."*	F3, F4
**eeg_data_loss_pct**	Not provided	
**epoch_rej_method**	Not provided	
**amplitude_threshold**	Not provided	
**clinical_pain_scale**	*"EEG recording, vital data and **Neonatal Infant Pain Scale (NIPS)** scoring were performed before and following painful stimulation via heel stick blood sampling during routine blood glucose measurements via glucometer, during non-nutritive suckling (NNS) and during suckling of sucrose."*	NIPS
**non_eeg_recording**	*"Analysis of obtained data revealed Significant rise in **heart rate**, lower*	Heart rate, Oxygen saturation, Blood glucose

	oxygen saturation *following nociceptive stimuli... The Results yielded a significant correlation between* ***random blood sugar levels****...”*	

Judith Meek (n=1 records)

35. Madeleine Verriotis, Lorenzo Fabrizi, Amy Lee, Robert Cooper, Maria Fitzgerald, Judith Meek. Mapping Cortical Responses to Somatosensory Stimuli in Human Infants with Simultaneous Near-Infrared Spectroscopy and Event-Related Potential Recording. eNeuro. 2016;3(2). doi:10.1523/ENEURO.0026-16.2016

Variable	***Data from publication***	***Data summary for review***
**publication_year**	2016	2016
**data_country**	*"Thirty-six healthy term infants were recruited from the postnatal ward and special care baby unit at **the Elizabeth Garrett Anderson Obstetric Wing, University College Hospital (UCH)**."*	United Kingdom
**sample_size**	*"Cortical activity was measured with simultaneous NIRS and EEG recordings in **30** infants."*	30
**pma_birth_avg**	Table 1: "*Age at birth (weeks)*" Note: this table presents the demographic information for n=36 infants, including infants that do not have EEG recordings.	39
**pma_study_avg**	Table 1: "*Age at study (weeks)*" Note: this table presents the demographic information for n=36 infants, including infants that do not have EEG recordings.	39.2
**sex_male_pct**	Calculated from the number of female infants reported in table 1. (36-15)/36*100= 58.33%	58.33
**sex_female_pct**	Table 1: "*Female infants*" Note: this table presents the demographic information for n=36 infants, including infants that do not have EEG recordings. 15/36*100= 41.67%	41.67
**pain_procedure**	*"Twenty-one infants were studied during a clinically required routine **heel lance**..."*	Heel lance
**analgesic_intervention**	Not applicable	
**electrode_placement_method**	Electrode positions were listed and 'cap' was not mentioned, so we assume individual electrodes placement method were used.	Individual electrodes
**electrode_placement_system**	*"Recording electrodes (disposable Ag/AgCl cup electrodes) were positioned according to a **modified international 10/20 electrode placement system** at Fp1, Fp2, Fz, F3, F4, Cz (vertex), C3, C4, CPz, CP3, CP4, T3, T4, T5, T6, O1, O2, and POz"*	Modified 10-20 system
**electrode_positions**	*"Recording electrodes (disposable Ag/AgCl cup electrodes) were positioned according to a modified international 10/20 electrode placement system at **Fp1, Fp2, Fz, F3, F4, Cz** (vertex), **C3, C4, CPz, CP3, CP4, T3, T4, T5, T6, O1, O2**, and **POz**... Reference and ground electrodes were placed at **FCz** and*	Fp1, Fp2, Fz, F3, F4, Cz, C3, C4, CPz, CP3, CP4, T7, T8, P7, P8, O1, O2, POz, FCz

	on the forehead, respectively". T3, T4, T5, T6 are standardised to T7, T8, P7, P8, respectively, according to the current version of the 10-10 system.	
**eeg_data_loss_pct**	The number of participants excluded due to artefact were not clear. ("*Five lance and five control trials were removed from the analysis for technical reasons (e.g., because the EEG was not performed or was of poor quality; or due to failed time locking of the stimulus to the EEG recording).*")	
**epoch_rej_method**	*"Channels containing a movement artifact (defined as activity exceeding 100 V) or high-frequency muscle activity were removed."*	Objective
**amplitude_threshold**	*"Channels containing a movement artifact (defined as* ***activity exceeding 100 V****) or high-frequency muscle activity were removed."*	Activity exceeding plus-minus 100 microV
**clinical_pain_scale**	Not applicable	
**non_eeg_recording**	*"...we have recorded* ***NIRS*** *and EEG simultaneously in individual healthy term babies"*	NIRS

Kiyoko Yokoyama (n=1 records)

45. Nusreena Hohsoh, Osuke Iwata, Tomoko Suzuki, Chinami Hanai, Ming Huang, Kiyoko Yokoyama. Quantification electroencephalography response to procedural pain during heel puncture in preterm infants. Physiol Meas. 2025;46(6):065004. doi:10.1088/1361-6579/addfa9

Variable	Data from publication	Data summary for review
**publication_year**		2025
**data_country**	*"A prospective cohort study was conducted on preterm infants (n = 42) in the NICU at Nagoya City University Hospital in* ***Japan*** *from January 2022 to April 2024."*	Japan
**sample_size**	*"A prospective cohort study was conducted on preterm infants (n =* ***42****) in the NICU at Nagoya City University Hospital in Japan from January 2022 to April 2024."*	42
**pma_birth_avg**	*"This study captured EEG signals from 42 preterm infants with a gestational age of* ***32.3*** *± 3.5 weeks..."*	32.3
**pma_study_avg**	Table 1: *"Postmenstrual age at puncture (weeks)* ***38.7*** *± 2.5"*	38.7
**sex_male_pct**	Table 1: *"Sex (male/female) 13/29"* Male percentage = 13/(13+29)*100 = 30.95%	30.95
**sex_female_pct**	Table 1: *"Sex (male/female) 13/29"* Female percentage = 29/(13+29)*100 = 69.05%	69.05
**pain_procedure**	"An automatic **heel lancet** (Quikeel Lancet, Becton, Dickinson and Company, USA) was used to puncture the lateral plantar surface of the left heel, followed by gentle squeezing of the heel to collect a blood sample."	Heel lance
**analgesic_intervention**	Not applicable	
**electrode_placement_method**	Electrode positions were listed and 'cap' was not mentioned, so we assume individual electrodes placement method were used.	Individual electrodes
**electrode_placement_system**	*"Eleven EEG electrodes (Fp1, Fp2, C3, Cz, C4, O1, O2, T3, T4, A1, and A2) were recorded according to the* ***10–20 international system*** *in this study"*	10–20 system
**electrode_positions**	*"Eleven EEG electrodes (****Fp1, Fp2, C3, Cz, C4, O1, O2, T3, T4, A1, and A2****) were recorded according to the 10–20 international system in this study"* T3, T4 are standardised to T7, T8, respectively, according to the current version of the 10-10 system.	Fp1, Fp2, C3, Cz, C4, O1, O2, T7, T8, A1, A2
**eeg_data_loss_pct**	Not applicable. Epochs were corrected instead of rejected. *"Amplitudes exceeding the defined threshold were replaced by linear*	

	interpolation" Not applicable. Epochs were corrected instead of rejected.	
**epoch_rej_method**	Not applicable. Epochs were corrected instead of rejected. *"Amplitudes exceeding the defined threshold were replaced by linear interpolation"* Not applicable. Epochs were corrected instead of rejected.	
**amplitude_threshold**	Not applicable. Epochs were corrected instead of rejected. *"Amplitudes exceeding the defined threshold were replaced by linear interpolation"* Not applicable. Epochs were corrected instead of rejected.	
**clinical_pain_scale**	Not applicable	
**non_eeg_recording**	Not applicable	

Laura Cornelissen (n=1 records)

3. Laura Cornelissen. Innovative Approaches to Assessment of Pain Control and Sedation in the NICU.; 2016. https://clinicaltrials.gov/study/NCT03057782

Variable	*Data from publication*	*Data summary for review*
**publication_year**	*"Study Start (Actual)<br>**2016**-10"*	2016
**data_country**	*"This study has 1 location<br>**United States**<br>Massachusetts Locations<br>Boston, Massachusetts, United States, 02115<br><br>Boston Children's Hospital"*	United States of America

Laura Jones (n=2 records)

29. Laura Jones, Maria Laudiano-Dray, Kimberley Whitehead, Madeleine Verriotis, Judith Meek, Maria Fitzgerald, Lorenzo Fabrizi. EEG, behavioural and physiological recordings following a painful procedure in human neonates. Sci Data. 2018;5(1):180248. doi:10.1038/sdata.2018.248

Variable	***Data from publication***	***Data summary for review***
**publication_year**	2018	2018
**data_country**	*"We present data from 112 infants (52 females; 29–47 weeks gestational age at study, 0.5–96 days postnatal age) recruited from the postnatal, special care, or intensive care wards at the* ***Elizabeth Garrett Anderson Obstetric Wing, University College London Hospital (UCLH)****."*	United Kingdom
**sample_size**	*"Cortical activity was recorded from* ***112*** *neonates (29–47 weeks gestational age at study) using a 20-channel electroencephalogram (EEG)..."*	112
**pma_birth_avg**	Table 1: *"Gestational age at birth (weeks + days)"*	35
**pma_study_avg**	Table 1: "*Gestational age at study (weeks + days)"*	36
**sex_male_pct**	Calculated from table 1: "*No. female 52 (****46%****)"* Male percentage 100-46= 54%	54
**sex_female_pct**	Table 1: "*No. female 52 (****46%****)"*	46
**pain_procedure**	*"Cortical activity was recorded from 112 neonates (29–47 weeks gestational age at study) using a 20-channel electroencephalogram (EEG), which was time-locked to a* ***heel lance****."*	Heel lance
**analgesic_intervention**	Not applicable	
**electrode_placement_method**	Electrode positions were listed and 'cap' was not mentioned, so we assume individual electrodes placement method were used.	Individual electrodes
**electrode_placement_system**	*"Recording electrodes were positioned according to a* ***modified international 10/10 electrode placement system****..."*	Modified 10-10 system
**electrode_positions**	*"...overlying primary visual (****O1, O2****), primary auditory (****T7, T8****), association (****F7, F3, F4, FCz, F8, P7, P8, TP9, TP10, POz****), and somatosensory cortices (****C3, Cz, C4, CP3, CPz, CP4****). The reference electrode was placed at* ***Fz*** *and the ground electrode at either FC1 or FC2 (depending on the position of the infant)."*	O1, O2, T7, T8, F7, F3, F4, FCz, F8, P7, P8, TP9, TP10, POz, C3, Cz, C4, CP3, CPz, CP4, Fz
**eeg_data_loss_pct**	"**Heel lance epochs are available for all 112 subjects**, sham and auditory controls are available for 99 subjects."	0
**epoch_rej_method**	*"Epochs contaminated with movement artifact (signal exceeding*	Objective

	± 100 µV) or delta brush activity (characterised by high voltage delta activity with over-riding alpha-beta oscillations, typically ranging between 50 and 300 µV), around the time of the nERP were removed."	
**amplitude_threshold**	*"Epochs contaminated with movement artifact (**signal exceeding ± 100 µV**) or **delta brush activity** (characterised by high voltage delta activity with over-riding alpha-beta oscillations, typically ranging between 50 and 300 µV), around the time of the nERP were removed."*	Signal exceeding plus-minus 100 microV
**clinical_pain_scale**	*"This data is linked to pain-related behaviour (facial expression), physiology (heart rate, oxygenation) and a composite clinical score (**Premature Infant Pain Profile, PIPP**). "*	PIPP/PIPP-R
**non_eeg_recording**	*"This data is linked to pain-related behaviour (**facial expression**), physiology (**heart rate, oxygenation**) and a composite clinical score (Premature Infant Pain Profile, PIPP)... To determine the PIPP score following the stimuli, the infant's pulse, blood oxygen saturation and facial expression were monitored using a pulse oximeter and a **video camera**...EEG was recorded according to clinical standards by an experienced clinical scientist. Clinical guidelines suggest recording ECG and **respiratory data** with the EEG."*	Video recording of facial expression, Heart rate, Oxygen saturation, Respiratory rate

46. Oana Bucsea, Mohammed Rupawala, Ilana Shiff, Xiaogang Wang, Judith Meek, Maria Fitzgerald, Lorenzo Fabrizi, Rebecca Pillai Riddell, Laura Jones. Clinical thresholds in pain-related facial activity linked to differences in cortical network activation in neonates. PAIN. 2023;164(5):1039. doi:10.1097/j.pain.0000000000002798

Variable	***Data from publication***	***Data summary for review***
**publication_year**	2023	2023
**data_country**	*"The current study draws on an archival sample of 78 neonates (41 late preterm and 37 full-term neonates [Table 1]), ranging from 0 to 14 days postnatal age recruited from the postnatal, special care, or intensive care wards at the Elizabeth Garett Anderson Obstetric Wing, University College London Hospital (UCLH) in London, **England**"*	United Kingdom
**sample_size**	*"The current study draws on an archival sample of **78** neonates..."*	78
**pma_birth_avg**	Table 1: "*GA (wk)":* (35.24+38.91)/2= 37.075	37.075
**pma_study_avg**	Table 1: "*PNA (d)"* Days are converted to weeks and added up to GA to get PMA (weeks). Average	37.87

	PNA = (6.1+5.03)/2 = 5.565 days = 5.565/7 = 0.795 weeks + 37.075 = 37.87 weeks	
**sex_male_pct**	Calculated from the number of female and sample size reported in Table 1: (78-(24+12))/78*100 = 53.85%	53.85
**sex_female_pct**	Table 1: "*Females*": (24+12)/78*100 = 46.15%	46.15
**pain_procedure**	*"Brain activity (up to 18 electrode EEG) and facial expressions (video) were recorded following a single clinically required noxious* ***heel lance*** *at bedside in the neonatal unit."*	Heel lance
**analgesic_intervention**	Not applicable	
**electrode_placement_method**	Electrode positions were listed and 'cap' was not mentioned, so we assume individual electrodes placement method were used.	Individual electrodes
**electrode_placement_system**	*"Electrodes were placed on the scalp according to* ***the international 10/20 electrode placement system****..."*	10-20 system
**electrode_positions**	*"...covering the primary visual (****O1 and O2****), primary auditory (****T7 and T8****), association (****F7, F3, F4, F8, P7, P8, TP9, and TP10****), and somatosensory (****C3, Cz, C4, CP3, CPz, and CP4****) scalp areas. A reference electrode was placed at* ***Fz*** *and the ground electrode at FC1/2, depending on the positioning of the infant during the procedure."*	O1, O2, T7, T8, F7, F3, F4, F8, P7, P8, TP9, TP10, C3, Cz, C4, CP3, CPz, CP4, Fz
**eeg_data_loss_pct**	*"Overall, only one participant had a missing NFCS-P-3 score and was thus excluded from the analyses."* One participant with EEG recording was excluded from analysis, but not due to artefact.	0
**epoch_rej_method**	Not applicable. Epochs were cleaned instead of rejected. *"Remaining artifacts (eg, high amplitude activity or ECG signals) were removed using independent component analysis in EEGLAB. Artifactual independent components were selected manually using the spatial maps and frequency content of the components."*	
**amplitude_threshold**	Not applicable	
**clinical_pain_scale**	*"Video footage of neonates' pain-related facial actions was coded using* ***the 7-item version of the Neonatal Facial Coding System*** *(NFCS) (brow bulge, eye squeeze, nasolabial furrow, open lips, vertical stretch mouth, horizontal stretch mouth, and taut tongue)."*	
**non_eeg_recording**	*"Brain activity (up to 18 electrode EEG) and* ***facial expressions (video)*** *were recorded following a single*	Video recording of facial expression

	clinically required noxious heel lance at bedside in the neonatal unit."	

Lorenzo Fabrizi (n=4 records)

30. Laura Jones, Maria Laudiano-Dray, Kimberley Whitehead, Judith Meek, Maria Fitzgerald, Lorenzo Fabrizi, Rebecca Pillai Riddell. The impact of parental contact upon cortical noxious-related activity in human neonates. European Journal of Pain. 2021;25(1):149-159. doi:10.1002/ejp.1656

Variable	***Data from publication***	***Data summary for review***
**publication_year**	2021	2021
**data_country**	*"Twenty-seven infants (23–41 gestational weeks at birth, 0–96 days old, 12 female; Table 1) were recruited from the postnatal, special care and high dependency wards within the neonatal unit at* ***University College London Hospital*** *between June 2015 and May 2018."*	United Kingdom
**sample_size**	*"**Twenty-seven** infants (23–41 gestational weeks at birth, 0–96 days old, 12 female; Table 1) were recruited from the postnatal, special care and high dependency wards within the neonatal unit at University College London Hospital between June 2015 and May 2018."*	27
**pma_birth_avg**	Table 1: "*GA (weeks) **33** (23–40) **35** (25–40) **34** (26–41)"* Average GA = (33+35+34)/3 = 34 weeks	34
**pma_study_avg**	Table 1: "*PNA (days) **20** (1–63) **24** (2–96) **14** (0–41)*" Average PNA (days) = (20+24+14)/3 = 19.33 days = 19.33/7 = 2.76 weeks. PMA(weeks) = average GA (see section 'pma_birth_avg') + average PNA(weeks) = 34+2.76 = 36.76 weeks	36.76
**sex_male_pct**	Percentage of males is calculated from percentage of females reported in Table 1: "*No. female 4 **(44%)** 4 **(44%)** 4 **(44%)***" Percentage of females = (44+44+44)/3 = 44%. Percentage of males = 100-44% = 56%	56
**sex_female_pct**	Table 1: "*No. female 4 **(44%)** 4 **(44%)** 4 **(44%)***" Percentage of females = (44+44+44)/3 = 44%	44
**pain_procedure**	*"EEG was recorded during a clinically required* ***heel lance*** *in three age and sex-matched groups of neonates (a) while held by a parent in skin-to-skin (n = 9), (b) while held by a parent with clothing (n = 9) or (c) not held at all, but in individualized care (n = 9)."*	Heel lance
**analgesic_intervention**	*"EEG was recorded during a clinically required heel lance in three age and sex-matched groups of neonates (a) while* ***held by a parent in skin-to-skin*** *(n = 9), (b) while held by a parent with clothing (n = 9) or (c) not held at all, but in individualized care (n = 9)."*	Parental holding

**electrode_placement_method**	*"**Recording electrodes were positioned individually** by a clinical neurophysiologist (KW) according to the international 10/20 electrode placement system (F7, F8, F3, F4, Cz, C3, C4, T7, T8, P7, P8, O1, O2), with additional central-parietal and temporal coverage (CPz, CP3, CP4, TP9, TP10)."*	Individual electrodes
**electrode_placement_system**	*"Recording electrodes were positioned individually by a clinical neurophysiologist (KW) according to **the international 10/20 electrode placement system** (F7, F8, F3, F4, Cz, C3, C4, T7, T8, P7, P8, O1, O2), with additional central-parietal and temporal coverage (CPz, CP3, CP4, TP9, TP10). Reference and ground electrodes were respectively placed at Fz and FC1/2."*	10-20 system
**electrode_positions**	*"Recording electrodes were positioned individually by a clinical neurophysiologist (KW) according to the international 10/20 electrode placement system (**F7, F8, F3, F4, Cz, C3, C4, T7, T8, P7, P8, O1, O2**), with additional central-parietal and temporal coverage (**CPz, CP3, CP4, TP9, TP10**). Reference and ground electrodes were respectively placed at **Fz** and FC1/2."*	F7, F8, F3, F4, Cz, C3, C4, T7, T8, P7, P8, O1, O2, CPz, CP3, CP4, TP9, TP10, Fz
**eeg_data_loss_pct**	Not provided	
**epoch_rej_method**	*"Epochs contaminated with movement artifact (signal exceeding ± 150 µV) were removed."*	Objective
**amplitude_threshold**	*"Epochs contaminated with movement artifact (**signal exceeding ± 150 µV**) were removed."*	Signal exceeding plus-minus 150 microV
**clinical_pain_scale**	Not applicable	
**non_eeg_recording**	*"Brain activity (electroencephalography, EEG), **facial response** (nasolabial furrow, eye-squeeze, and brow bulge) and **heart rate** (electrocardiography, ECG) to a single clinically -required noxious heel lance were recorded."*	Video recording of facial expression, Heart rate

32. Lorenzo Fabrizi, Rebeccah Slater, Alan Worley, Judith Meek, Stewart Boyd, Sofia Olhede, Maria Fitzgerald. A Shift in Sensory Processing that Enables the Developing Human Brain to Discriminate Touch from Pain. Current Biology. 2011;21(18):1552-1558. doi:10.1016/j.cub.2011.08.010

Variable	*Data from publication*	*Data summary for review*
**publication_year**	2011	2011
**data_country**	*"Forty-six infants, recruited from the intensive care unit, special care baby unit, and postnatal ward at the **Elizabeth Garrett Anderson and Obstetric Hospital**, participated in this study."*	United Kingdom

**sample_size**	*"**Forty-six** infants, recruited from the intensive care unit, special care baby unit, and postnatal ward at the Elizabeth Garrett Anderson and Obstetric Hospital, participated in this study."* And from Table 1, *"Number of infants **46...** The occurrence of tactile and nociceptive-specific potentials and of delta brushes was assessed on 60 EEG recordings following time-locked touch or noxious lance of the heel of **41** infants"* The number of babies receiving heel lance and was analysed was reported as '41'. It is unclear if the other 5 among the 46 babies had their EEG recorded.	41
**pma_birth_avg**	Table 1: *"Mean (SD) GA at birth (weeks), n = 46 **35.1** (5.4); range 24–41.6"*	35.1
**pma_study_avg**	Table 1: *"Mean (SD) GA at time of study (weeks), n = 68 **37.3** (3.7); range 28.4–45.3"* It is the mean of all trials (n=68), not the mean of all infants (n =64).	37.3
**sex_male_pct**	Table 1: "Percentage of males, n = 46 **60**"	60
**sex_female_pct**	Percentage of females are calculated as 100 – percentage of males. Table 1: "Percentage of males, n = 46 **60**" Percentage of females = 100-60 = 40%.	40
**pain_procedure**	*"We have conducted noninvasive electroencephalogram (EEG) recording of the brain neuronal activity in response to time-locked touches and clinically essential **noxious lances of the heel** in infants aged 28–45 weeks gestation."*	Heel lance
**analgesic_intervention**	Not applicable	
**electrode_placement_method**	Electrode positions were listed and 'cap' was not mentioned, so we assume individual electrodes placement method were used.	Individual electrodes
**electrode_placement_system**	*"Recording electrodes (disposable Ag/AgCl cup electrodes) were positioned according to the **modified international 10/20 electrode placement system** at F7, F8, Cz, CPz, C3, C4, CP3, CP4, T3, T4, T5, T6, O1, and O2."*	Modified 10-20 system
**electrode_positions**	*"Recording electrodes (disposable Ag/AgCl cup electrodes) were positioned according to the modified international 10/20 electrode placement system at **F7, F8, Cz, CPz, C3, C4, CP3, CP4, T3, T4, T5, T6, O1, and O2**... Reference and ground electrodes were placed at **FCz** and the chest, respectively."* T3, T4, T5, T6 are standardised to T7, T8, P7, P8, respectively,	F7, F8, Cz, CPz, C3, C4, CP3, CP4, T7, T8, P7, P8, O1, O2, FCz

	according to the current version of the 10-10 system.	
**eeg_data_loss_pct**	The number of trials excluded due to artefact was reported, but the number of babies is unclear. *"A total of 68 noxious heel lances were analyzed; of these, 3 were excluded from analysis because automatic event marking of the EEG did not occur and 5 because of the presence of movement artifacts in the EEG recording."*	
**epoch_rej_method**	Not provided. *"A total of 68 noxious heel lances were analyzed; of these, 3 were excluded from analysis because automatic event marking of the EEG did not occur and 5 because of the presence of movement artifacts in the EEG recording."*	
**amplitude_threshold**	Not provided	
**clinical_pain_scale**	Not applicable	
**non_eeg_recording**	Not applicable	

33. Lorenzo Fabrizi, Madeleine Verriotis, Gemma Williams, Amy Lee, Judith Meek, Sofia Olhede, Maria Fitzgerald. Encoding of mechanical nociception differs in the adult and infant brain. Sci Rep. 2016;6(1):28642. doi:10.1038/srep28642

Variable	***Data from publication***	***Data summary for review***
**publication_year**	2016	2016
**data_country**	*"Eighteen healthy full term 0–19 day old (5.8 ± 4.3, mean ± SD) infants (twelve males; born at 37–42 weeks gestational age) from the **Elizabeth Garrett Anderson and Obstetric Hospital** were included in this study."*	United Kingdom
**sample_size**	*"**Eighteen** healthy full term 0–19 day old (5.8 ± 4.3, mean ± SD) infants (twelve males; born at 37–42 weeks gestational age) from the Elizabeth Garrett Anderson and Obstetric Hospital were included in this study."*	18
**pma_birth_avg**	Not provided. Only range of gestational age was provided. *"Eighteen healthy full term 0–19 day old (5.8 ± 4.3, mean ± SD) infants (twelve males; **born at 37–42 weeks gestational age**) from the Elizabeth Garrett Anderson and Obstetric Hospital were included in this study."*	
**pma_study_avg**	Not provided. Only mean of postnatal age and range of gestational age was provided. *"Eighteen healthy full term 0–19 day old (**5.8** ± 4.3, mean ± SD) infants (twelve males; **born at 37–42 weeks gestational age**) from the Elizabeth Garrett Anderson and Obstetric*	

	Hospital were included in this study."	
**sex_male_pct**	*"Eighteen healthy full term 0–19 day old (5.8 ± 4.3, mean ± SD) infants (**twelve males**; born at 37–42 weeks gestational age) from the Elizabeth Garrett Anderson and Obstetric Hospital were included in this study."* Percentage of males = 12/18*100 = 66.67%	66.67
**sex_female_pct**	Percentage of females is calculated from the number of males provided in the paper. *"Eighteen healthy full term 0–19 day old (5.8 ± 4.3, mean ± SD) infants (**twelve males**; born at 37–42 weeks gestational age) from the Elizabeth Garrett Anderson and Obstetric Hospital were included in this study."* Percentage of females = (18-12)/18*100 = 33.33%	33.33
**pain_procedure**	*"For all **heel lances**, standard hospital practice was followed"*	Heel lance
**analgesic_intervention**	Not applicable	
**electrode_placement_method**	Electrode positions were listed and 'cap' was not mentioned, so we assume individual electrodes placement method were used.	Individual electrodes
**electrode_placement_system**	*"Recording electrodes (disposable Ag/AgCl cup electrodes) were positioned according to **the modified international 10/20 electrode placement system** at Fp1, Fp2, F7, F8, Fz, Cz, CPz, C3, C4, CP3, CP4, T3, T4, T5, T6, O1 and O2."*	Modified 10-20 system
**electrode_positions**	*"Recording electrodes (disposable Ag/AgCl cup electrodes) were positioned according to the modified international 10/20 electrode placement system at **Fp1, Fp2, F7, F8, Fz, Cz, CPz, C3, C4, CP3, CP4, T3, T4, T5, T6, O1 and O2**. Recordings were referenced to **FCz** (all infants and eleven adults) or to linked earlobes (ten adults) and were all re-referenced to Fz post-acquisition."* T3, T4, T5, T6 are standardised to T7, T8, P7, P8, respectively, according to the current version of the 10-10 system.	Fp1, Fp2, F7, F8, Fz, Cz, CPz, C3, C4, CP3, CP4, T7, T8, P7, P8, O1, O2, FCz
**eeg_data_loss_pct**	*"**Two infant lance epochs** were rejected due to movement artefact...Eleven epochs were rejected due to movement artefacts (1 adult lance, 1 adult control, **6 infant lances** and 1 infant control epochs)."* Since there are two analyses, the number of data loss is averaged among the 2 analyses= (2+6)/2 = 4. Percentage of data loss = 4/18 *100 = 22.22%	22.22
**epoch_rej_method**	*"Epochs contaminated by gross movement artefacts (**signal***	Objective

	exceeding ± 100 μ V at any time point *or* ***larger than 2* SD of the baseline period for more than ¾ of the epoch length****) were rejected."*	
**amplitude_threshold**	*"Epochs contaminated by gross movement artefacts (****signal exceeding ± 100 μ V at any time point*** *or larger than 2* SD of the baseline period for more than ¾ of the epoch length) were rejected."*	Signal exceeding plus-minus 100 microV at any time point or larger than 2* SD of the baseline period for more than three-fourths of the epoch length
**clinical_pain_scale**	Not applicable	
**non_eeg_recording**	Not applicable	

42. Mohammed Rupawala, Oana Bucsea, Maria Laudiano-Dray, Kimberley Whitehead, Judith Meek, Maria Fitzgerald, Sofia Olhede, Laura Jones, Lorenzo Fabrizi. A developmental shift in habituation to pain in human neonates. Current Biology. 2023;33(8):1397-1406.e5. doi:10.1016/j.cub.2023.02.071

Variable	***Data from publication***	***Data summary for review***
**publication_year**	2023	2023
**data_country**	*"This was an opportunistic sample from a larger database of 283 infants who were recruited from the Maternity and Neonatal Units at* ***University College London Hospitals (UCLH)*** *over 12 years between December 2007 and November 2019."*	United Kingdom
**sample_size**	*"****Twenty*** *human infants (Table 1) underwent two blood tests (heel lances) in brief succession (3–18 min separation, as clinically required) in the same heel skin area (Figure 1)."*	20
**pma_birth_avg**	Table 1: "*GA at birth (weeks+days)* ***31+66*** *(24+22–34+5)* ***38+0*** *(31+66–41+4)".* There seems to be a typography in the number of days; we assume '31+66' is '31+6', which is consistent with the preprint (https://doi.org/10.1101/2022.04.05.486988): Table 1: "*GA at birth (weeks+days)* ***31+6*** *(24+2– 34+5)* ***38+0*** *(24+6– 41+4)*". 'Weeks+days' unit is converted to 'weeks' = 31+(6/7)=31.86 and 38, then averaged= (31.86+38)/2 = 34.93.	34.93
**pma_study_avg**	Table 1: "*PMA at study (weeks+days)* ***34+0*** *(32+66–36+4)* ***39+44*** *(37+44–44+2)".* There seems to be a typography in the number of days; we assume '39+44' is '39+4' 'Weeks+days' unit is converted to 'weeks' = 34 and 39+(4/7)= 39.57, then averaged= (34+39.57)/2= 36.79	36.79
**sex_male_pct**	Percentage of males is calculated from the percentage of females provided. Table 1: "*Number of female 5 (****50%****) 4 (****40%****)"* Average percentage of females = (50+40)/2 = 45%. Average percentage of males= 100%-45% = 55%	55
**sex_female_pct**	Table 1: "*Number of female 5 (****50%****) 4 (40%)"* Average percentage of females = (50+40)/2 = 45%	45

**pain_procedure**	*"Here, we used electroencephalography to investigate changes in cortical microstates (representing the complex sequential processing of noxious inputs) following two consecutive clinically required* ***heel lances*** *in term and preterm infants."*	Heel lance
**analgesic_intervention**	Not applicable	
**electrode_placement_method**	Electrode positions were listed and 'cap' was not mentioned, so we assume individual electrodes placement method were used.	Individual electrodes
**electrode_placement_system**	*"The electroencephalogram (EEG) was recorded from a subset of 19 recording electrodes (disposable Ag/AgCl cup electrodes) from* ***the international 10/20 electrode placement system****."*	10-20 system
**electrode_positions**	*"Those included electrodes overlying primary visual (**O1, O2**), primary auditory (**T7, T8**), association (**F7, F3, Fz, F4, F8, P7, P8, T9, TP10**), and somatosensory (**C3, Cz, C4, CP3, CPz, CP4**) cortices (Figure 1A)... The reference electrode was placed at **FCz** or **Fz** and the ground electrode at FC1 or FC2 (depending on the position of the infant)."* T9 is corrected to TP9, as in Figure 1A.	Fz, F3, F4, F7, F8, Cz, C3, C4, T7, T8, CPz, CP3, CP4, TP9, TP10, P7, P8, O1, O2, FCz
**eeg_data_loss_pct**	Not provided	
**epoch_rej_method**	Not applicable. Epochs were cleaned instead of rejected. *"Raw data were subsequently de-noised using independent component analysis (between 0-3 discrete independent components per trial were removed corresponding to ECG breakthrough or muscle, movement or equipment artifacts, median: 0). Artifactual independent components were selected manually using the spatial maps and frequency content of the components."*	
**amplitude_threshold**	Not applicable	
**clinical_pain_scale**	*"Videos were epoched between 10 s prior to and 10 s following the stimulus and infant facial expression were scored second-by-second according to* ***the 7-item version of the Neonatal Facial Coding System (NFCS)*** *(brow bulge, eye squeeze, nasolabial furrow, open lips, vertical stretch mouth, horizontal stretch mouth, and taut tongue)."* NFCS is not extracted as a clinical pain scale in this data extraction, since not all component of NFCS is used.	
**non_eeg_recording**	*"Brain electrical activity at the scalp (electroencephalography, EEG), flexion withdrawal reflex of the lanced leg (surface electromyography,* ***EMG****),* ***heart rate*** *changes (electrocardiography, ECG) and* ***facial expressions (video)*** *time-locked to the clinically-required heel lances were recorded"*	EMG, Heart rate, Video recording of facial expression

Luke Baxter (n=1 records)

23. Gabriela Schmidt Mellado, Kirubin Pillay, Eleri Adams, Ana Alarcon, Foteini Andritsou, Maria Cobo, Ria Evans Fry, Sean Fitzgibbon, Fiona Moultrie, Luke Baxter, Rebeccah Slater. The impact of premature extrauterine exposure on infants' stimulus-evoked brain activity across multiple sensory systems. NeuroImage: Clinical. 2022;33:102914. doi:10.1016/j.nicl.2021.102914

Variable	*Data from publication*	*Data summary for review*
**publication_year**	2022	2022
**data_country**	*"Infants born between February 2018 to February 2020 from the Maternity Unit and Special Care Baby Unit at the John Radcliffe Hospital, Oxford, **UK**, were enrolled in this study after informed, written consent was obtained from the parents."*	United Kingdom
**sample_size**	Fig.1: number of babies who received skin-breaking procedure: n=10 in orange group, n=7 in yellow group, n=0 in green group. Total= 10+7+0= 17.	17
**pma_birth_avg**	Table 1, but since no babies in the 'Full-term' group received skin-breaking procedures (see Fig 1), so only 'Very preterm' and 'Late preterm' group is extracted in this section. Note that one baby in 'Late preterm' group does not receive skin-breaking procedure, but this Table 1 includes that baby. "*Gestational age at birth (weeks) 38.2 (36.1–41.0) **29.0** (27.1–31.4) **34.7** (34–35.7)*". Average PMA at birth = (29+34.7)/2 = 31.85	31.85
**pma_study_avg**	Table 1, but since no babies in the 'Full-term' group received skin-breaking procedures (see Fig 1), so only 'Very preterm' and 'Late preterm' group is extracted in this section. Note that one baby in 'Late preterm' group does not receive skin-breaking procedure, but this Table 1 includes that baby. For 'Very preterm' group, PMA at the first test is taken in this data extraction. *"Postmenstrual age at study (weeks) 38.5 (36.6–41.7) **30.4** (28–31.9) 32.7 (32–33.3) 35.1 (34.3–36.3) **35.5** (34.7–36.4)"* Average of PMA at study (weeks) = (30.4+35.5)/2 = 32.95.	32.95
**sex_male_pct**	Percentage of males is calculated from the number of females presented in Table 1. Since no babies in the 'Full-term' group received skin-breaking procedures (see Fig 1), so only 'Very preterm' and 'Late preterm' group is extracted in this section. Note that one baby in 'Late preterm' group does not receive	55.56

	skin-breaking procedure, but this Table 1 includes that baby. *"Female 6* ***5 3****"* Percentage of males = (total number of participants in very preterm and later preterm group - number of females in very preterm and late preterm group)/ (total number of participants in very preterm and later preterm group) *100 = ((10+8)-(5+3))/(10+8) *100 = 10/18*100 = 55.56%	
**sex_female_pct**	Table 1, but since no babies in the 'Full-term' group received skin-breaking procedures (see Fig 1), so only 'Very preterm' and 'Late preterm' group is extracted in this section. Note that one baby in 'Late preterm' group does not receive skin-breaking procedure, but this Table 1 includes that baby. *"Female 6* ***5 3****"* Percentage of females = (number of females in very preterm and late preterm group)/ (total number of participants in very preterm and later preterm group) *100 = (5+3)/(10+8) *100 = 44.44%	44.44
**pain_procedure**	*"Experimental or clinical noxious stimuli were applied depending on the cohort of infants and clinical procedures required. The two preterm cohorts (Fig. 1, Very preterm and Late preterm) had a clinical noxious stimulus in the form of a* ***heel lance*** *(BD Quikheel Preemie Lancet – Becton, Dickinson and Company) as they clinically required blood sampling at the time of study... In the healthy full-term cohort (Fig. 1, Full-term), no infants required a clinical noxious stimulus, so a mild 128 mN experimental sharp-touch stimulus (PinPrick Stimulator, MRC Systems) was applied to the heel of the foot instead (20–30 trials)."*	Heel lance
**analgesic_intervention**	Not applicable	
**electrode_placement_method**	Electrode positions were listed and 'cap' was not mentioned, so we assume individual electrodes placement method were used.	Individual electrodes
**electrode_placement_system**	*"Electrodes were placed according to* ***the 10–20 International Electrode Measuring System****"*	10-20 system
**electrode_positions**	*"Channels included* ***Fp1, Fp2, F3, F4, F7, F8, FCz, T3, T4, C3, C4, Cz, CP3, CP4, CPz, T5, T6, P3, P4, Pz, O1, O2, Oz, A1 and A2*** *electrodes, with* ***Fz*** *as reference and FPz as ground."* T3, T4, T5, T6 are standardised to T7, T8, P7, P8,	Fp1, Fp2, F3, F4, F7, F8, FCz, T7, T8, C3, C4, Cz, CP3, CP4, CPz, P7, P8, P3, P4, Pz, O1, O2, Oz, A1, A2, Fz

	respectively, according to the current version of the 10-10 system.	
**eeg_data_loss_pct**	Number of participants that were excluded due to artefact was not reported; only the number of trials was reported. *"In total, 6 heel lance trials out of 36 were rejected due to artefact."*	
**epoch_rej_method**	*"All trials were visually assessed for quality by a trained clinical neurophysiologist (GSM). Trials were rejected only* ***if the pre-stimulus baseline or the post-stimulus activity contained gross movement arte-facts (e.g. signal amplitudes > 800 µV on any channel), or if the pre-stimulus baseline was unsettled (i.e. when stimulation was preceded by spontaneous bursting or other activity in the background or when EEG activity prior to stimulus was fluctuating > 20 µV)****."*	Mixed
**amplitude_threshold**	*"All trials were visually assessed for quality by a trained clinical neurophysiologist (GSM). Trials were rejected only* ***if the pre-stimulus baseline or the post-stimulus activity contained gross movement arte-facts (e.g. signal amplitudes > 800 µV on any channel), or if the pre-stimulus baseline was unsettled (i.e. when stimulation was preceded by spontaneous bursting or other activity in the background or when EEG activity prior to stimulus was fluctuating > 20 µV)****."*	Signal amplitudes > 800 µV on any channel, or when stimulation was preceded by spontaneous bursting or other activity in the background, or when EEG activity prior to stimulus was fluctuating > 20 µV
**clinical_pain_scale**	Not applicable	
**non_eeg_recording**	Not applicable	

Maria Fitzgerald (n=3 records)

28. Laura Jones, Lorenzo Fabrizi, Maria Laudiano-Dray, Kimberley Whitehead, Judith Meek, Madeleine Verriotis, Maria Fitzgerald. Nociceptive Cortical Activity Is Dissociated from Nociceptive Behavior in Newborn Human Infants under Stress. Current Biology. 2017;27(24):3846-3851.e3. doi:10.1016/j.cub.2017.10.063

Variable	***Data from publication***	***Data summary for review***
**publication_year**	2017	2017
**data_country**	*"Fifty-six healthy term born infants (29 males; 36–42 weeks corrected age, mean 38 weeks + 5 days) aged between 0.5–14 days (3.9 ± 2.4, mean ± SD) were recruited from the postnatal ward and special care baby unit at* ***the Elizabeth Garrett Anderson Obstetric Wing, University College Hospital (UCH).****"*	United Kingdom
**sample_size**	*Figure S2: "Of the 56 infants recruited:* ***49*** *had EEG recorded…"*	49
**pma_birth_avg**	The data reported was for the 56 recruited babies, so including those without EEG recording. Mean GA/ PMA at birth = mean "*corrected age*"-mean postnatal age = **(38 weeks + 5 days)-3.9 days** = 38 weeks + 1.1 days = 38 + (1.1/7) = 38.16 weeks	38.16
**pma_study_avg**	The data reported was for the 56 recruited babies, so including those without EEG recording. *"Fifty-six healthy term born infants (29 males; 36–42 weeks corrected age,* ***mean 38 weeks + 5 days****) aged between 0.5–14 days (3.9 ± 2.4, mean ± SD) were recruited from the postnatal ward and special care baby unit at the Elizabeth Garrett Anderson Obstetric Wing, University College Hospital (UCH)." 38 weeks + 5 days are converted to weeks = 38 + (5/7) = 38.71 weeks.*	38.71
**sex_male_pct**	The data reported was for the 56 recruited babies, so including those without EEG recording. *"****Fifty-six*** *healthy term born infants (****29*** *males; 36–42 weeks corrected age, mean 38 weeks + 5 days) aged between 0.5–14 days (3.9 ± 2.4, mean ± SD) were recruited from the postnatal ward and special care baby unit at the Elizabeth Garrett Anderson Obstetric Wing, University College Hospital (UCH)."* Male percentage = 29/56 *100 = 51.79%.	51.79
**sex_female_pct**	The data reported was for the 56 recruited babies, so including those without EEG recording. *"****Fifty-six*** *healthy term born infants (****29*** *males; 36–42 weeks corrected age, mean 38 weeks + 5 days) aged between 0.5–*	48.21

	14 days (3.9 ± 2.4, mean ± SD) were recruited from the postnatal ward and special care baby unit at the Elizabeth Garrett Anderson Obstetric Wing, University College Hospital (UCH).” Female percentage is calculated from number of male. Total female = 56-29 = 27. Female percentage = 27/56 *100 = 48.21%.	
**pain_procedure**	*“The noxious stimulus was a* ***heel lance*** *that was clinically required to collect a blood sample.”*	Heel lance
**analgesic_intervention**	Not applicable	
**electrode_placement_method**	Electrode positions were listed and ‘cap’ was not mentioned, so we assume individual electrodes placement method were used.	Individual electrodes
**electrode_placement_system**	*“EEG recording: Standard electrode placement, included nineteen electrodes (disposable Ag/AgCl cup electrodes) that were placed according to the* ***modified international 10/20 system*** *at F7, F3, T7, O1, F4, F8, T8, O2, C3, Cz, C4, CPz, CP3, CP4, TP9, TP10, P7, P8, and FCz.”*	Modified 10-20 system
**electrode_positions**	*“EEG recording: Standard electrode placement, included nineteen electrodes (disposable Ag/AgCl cup electrodes) that were placed according to the modified international 10/20 system at* ***F7, F3, T7, O1, F4, F8, T8, O2, C3, Cz, C4, CPz, CP3, CP4, TP9, TP10, P7, P8, and FCz.*** *Reference and ground electrodes were respectively placed at* ***Fz*** *and FC6/5.”*	F7, F3, T7, O1, F4, F8, T8, O2, C3, Cz, C4, CPz, CP3, CP4, TP9, TP10, P7, P8, FCz, Fz
**eeg_data_loss_pct**	*“The median N3P3 peak-to-peak amplitude of the whole sample, including non-responders, was 44.18 mV (range: 0–146.88 mV, n = 49).”* The number of babies with EEG analysed is the same with the number of babies with EEG recording (see ‘sample size’ section of this table), so there is 0 data loss.	0
**epoch_rej_method**	*“Epochs contaminated with move-ment artifact (****signal exceeding*** ***± 100cμV****) were rejected.”* It was assumed that ‘cμV’ was a printing typography, and so was interpreted as ‘μV’.	Objective
**amplitude_threshold**	*“Epochs contaminated with move-ment artifact (****signal exceeding*** ***± 100cμV****) were rejected.”* It was assumed that ‘cμV’ was a printing typography, and so was interpreted as ‘μV’.	Signal exceeding plus-minus 100 microV
**clinical_pain_scale**	*“A* ***PIPP score*** *was calculated for each test occasion combining behavioral and physiological*	PIPP/PIPP-R

	measures"	
**non_eeg_recording**	*"Behavior was measured using noxious evoked **facial grimaces** scored from **video recordings**…The overall pain score (premature infant pain profile [PIPP], a composite behavioral and physiological measure) was also calculated for each baby. The physiological (**heart rate** and **oxygen saturation**) response was included for completeness…Brain activation, behavioral and physiological responses to a clinically required noxious heel lance and the background **salivary cortisol level** and **heart rate** variability (sampled before and after the lance) were recorded"* and Figure S1.	Video recording of facial expression, Heart rate, Salivary cortisol, Oxygen saturation

31. Lorenzo Fabrizi, Rebeccah Slater, Alan Worley, Judith Meek, Sofia Olhede, Stewart Boyd, Maria Fitzgerald. P14-24 Development of a cortical electrophysiological response to noxious stimulation in human infants. Clinical Neurophysiology. 2010;121:S190. doi:10.1016/S1388-2457(10)60782-1

Variable	*Data from publication*	*Data summary for review*
**publication_year**	2010	2010
**data_country**	Based on the authors' affiliation	United Kingdom
**sample_size**	*"nERP was defined from EEG epochs (n = 23) recorded following noxious heel lances at electrodes CPz of **18 term infants** (37 46-week PMA) using Principal Component (PC) Analysis. The weights of the defined nERP were calculated in equivalent EEG epochs (n = 63) at 16 electrode positions from **44 infants** (28 46-week)." Total number of babies with EEG recording = 18 + 44 = 62.*	62
**pma_birth_avg**	Not provided	
**pma_study_avg**	Not provided. Only range was provided. *"nERP was defined from EEG epochs (n = 23) recorded following noxious heel lances at electrodes CPz of 18 term infants (37 46-week PMA) using Principal Component (PC) Analysis. The weights of the defined nERP were calculated in equivalent EEG epochs (n = 63) at 16 electrode positions from 44 infants (28 46-week)."*	
**sex_male_pct**	Not provided	
**sex_female_pct**	Not provided	
**pain_procedure**	*"To describe age related changes in scalp potentials evoked by clinically essential noxious **heel lances** in human infants."*	Heel lance
**analgesic_intervention**	Not applicable	
**electrode_placement_method**	Not provided	

**electrode_placement_system**	Not provided	
**electrode_positions**	*"nERP was defined from EEG epochs (n = 23) recorded following noxious heel lances at electrodes* ***CPz*** *of 18 term infants (37 46-week PMA) using Principal Component (PC) Analysis… The associated PC weights had a significant increasing linear trend with respect to PMA at* ***CPz/Cz*** *(0.008 and 0.006 week 1; p< 0.05)."*	CPz, Cz
**eeg_data_loss_pct**	Not provided	
**epoch_rej_method**	Not provided	
**amplitude_threshold**	Not provided	
**clinical_pain_scale**	Not applicable	
**non_eeg_recording**	Not applicable	

36. Madeleine Verriotis, Laura Jones, Kimberley Whitehead, Maria Laudiano-Dray, Ismini Panayotidis, Hemani Patel, Judith Meek, Lorenzo Fabrizi, Maria Fitzgerald. The distribution of pain activity across the human neonatal brain is sex dependent. NeuroImage. 2018;178:69-77. doi:10.1016/j.neuroimage.2018.05.030

Variable	***Data from publication***	***Data summary for review***
**publication_year**	2018	2018
**data_country**	*"Babies were recruited from the postnatal, special care, or intensive care wards at* ***the Elizabeth Garrett Anderson Obstetric Wing, University College London Hospital (UCLH)*** *between 2007 and 2016."*	United Kingdom
**sample_size**	*"We measured the cortical response time-locked to a clinically required heel lance in* ***81*** *neonates born between 29 and 42 weeks gestational age (median postnatal age 4 days)."*	81
**pma_birth_avg**	Table 1: "*Gestational age at birth (weeks)* ***36*** *(29–42)*"	36
**pma_study_avg**	Table 1: "*Age at study (weeks)* ***37*** *(29–43)"*	37
**sex_male_pct**	The percentage of male is calculated from the percentage of male reported in Table 1. Table 1: *"No. female 34 (42%)"* Percentage of male = 100-42 = 58%	58
**sex_female_pct**	Table 1: *"No. female 34 (**42%**)"*	42
**pain_procedure**	*"We measured the cortical response time-locked to a clinically required* ***heel lance*** *in 81 neonates born between 29 and 42 weeks gestational age (median postnatal age 4 days)."*	Heel lance
**analgesic_intervention**	Not applicable	
**electrode_placement_method**	*"Recording electrodes (Ambu Neuroline disposable Ag/AgCl cup* ***electrodes) were positioned individually****"*	Individual electrodes
**electrode_placement_system**	*"Recording electrodes (Ambu Neuroline disposable Ag/AgCl cup electrodes) were positioned individually by a clinical physiologist according to a* ***modified***	Modified 10-10 system

	international 10/10 electrode placement system"	
**electrode_positions**	*"Recording electrodes (Ambu Neuroline disposable Ag/AgCl cup electrodes) were positioned individually by a clinical physiologist according to a modified international 10/10 electrode placement system at* ***Fp1, Fp2, F3, F4, F7, F8, FCz, Cz, C3, C4, T7, T8, CPz, CP3, CP4, TP9, TP10, P7, P8, POz, O1, and O2****... The reference electrode was placed at either* ***FCz*** *or* ***Fz****, and all trials were re-referenced to Fz to allow comparison across trials. The ground electrode was placed on the chest, forehead, or at FC1/2."*	Fp1, Fp2, F3, F4, F7, F8, FCz, Cz, C3, C4, T7, T8, CPz, CP3, CP4, TP9, TP10, P7, P8, POz, O1, O2, Fz
**eeg_data_loss_pct**	*Table 2: "The incidence of a nERP at the vertex was assessed in the full sample (****n = 81****). Twelve babies without a response at the vertex were removed from subsequent analysis. Of the remaining 69 babies,* ***2 were removed because they did not have enough electrodes, after channel rejections****, for assessment of their distribution pattern, and 4 were removed from the regression analysis because they were classified as having a "focused elsewhere" distribution pattern, which was too small a group for the regression analysis."* Percentage of data loss = 2/81 *100 = 2.47%.	2.47
**epoch_rej_method**	*"EEG activity was inspected visually, and* ***channels containing movement artefact (defined as activity exceeding 100 µV), high-frequency muscle activity, or delta brush bursting activity*** *(characterised by high voltage delta activity with over-riding alpha-beta oscillations), around the time of the nERP were removed"*	Objective
**amplitude_threshold**	*"EEG activity was inspected visually, and channels containing movement artefact (defined as* ***activity exceeding 100 µV****), high-frequency muscle activity, or delta brush bursting activity (characterised by high voltage delta activity with over-riding alpha-beta oscillations), around the time of the nERP were removed"*	Activity exceeding plus-minus 100 microV
**Non_amplitude_rej_method**	*"EEG activity was inspected visually, and channels containing movement artefact (defined as activity exceeding 100 µV),* ***high-frequency muscle activity****, or delta brush bursting activity (char-*	High-frequency muscle activity

	acterised by high voltage delta activity with over-riding alpha-beta oscillations), around the time of the nERP were removed"	
**clinical_pain_scale**	Not applicable	
**non_eeg_recording**	Not applicable	

Madeleine Verriotis (n=1 records)

34. Madeleine Verriotis, Lorenzo Fabrizi, Amy Lee, Sheryl Ledwidge, Judith Meek, Maria Fitzgerald. Cortical activity evoked by inoculation needle prick in infants up to one-year old. Pain. 2015;156(2):222-230. doi:10.1097/01.j.pain.0000460302.56325.0c

Variable	*Data from publication*	*Data summary for review*
**publication_year**	2015	2015
**data_country**	*"Infants were recruited from outpatient clinics at the Elizabeth Garrett Anderson Wing, University College London Hospital."*	United Kingdom
**sample_size**	*"Fifteen healthy term-born infants were* ***studied at 1 to 2 months (n= 12)*** *or at 12 months (n= 5) of age. Two infants were studied at both ages, and 1 infant was studied at both 1 and 2 months."* Infants studied at 12 months are not included in sample size because this review focuses on newborns.	12
**pma_birth_avg**	Table 1: "Age at birth, completed wk" for "*1- to 2-month-olds*"	38.9
**pma_study_avg**	Table 1: "*Age at study, mo*" Number is converted to weeks unit and are added up to age at birth. 1.4months = 1.4*4= 5.6weeks, 5.6+38.9= 44.5weeks	44.5
**sex_male_pct**	Calculated from sample size and the number of female infants in Table 1: (12-6)/12*100= 50%	50
**sex_female_pct**	Table 1: "*No. of female infants* ***6/12***" 6/12*100= 50%	50
**pain_procedure**	*"The noxious stimulus was a needle puncture as part of a clinically required routine* ***inoculation*** *administered intramuscularly into the thigh by a nurse or doctor."*	Immunization
**analgesic_intervention**	Not applicable	
**electrode_placement_method**	Electrode positions were listed and 'cap' was not mentioned, so we assume individual electrodes placement method were used.	Individual electrodes
**electrode_placement_system**	*"Recording electrodes (disposable Ag/AgCl cup electrodes) were positioned according to* ***the modified international 10/20 electrode placement system*** *at Fp1, Fp2, Fz, F3, F4, Cz, C3, C4, CPz, CP3, CP4, T3, T4, T5, T6, O1, O2, and POz."*	Modified 10-20 system
**electrode_positions**	*"Recording electrodes (disposable Ag/AgCl cup electrodes) were positioned according to the modified international 10/20 electrode placement system at* ***Fp1, Fp2, Fz, F3, F4, Cz, C3, C4, CPz, CP3, CP4, T3, T4, T5, T6, O1, O2, and POz****... Reference and ground electrodes were placed at* ***FCz*** *and on the forehead, respectively."* T3, T4, T5, T6 are standardised to T7, T8, P7,	Fp1, Fp2, Fz, F3, F4, Cz, C3, C4, CPz, CP3, CP4, T7, T8, P7, P8, O1, O2, POz, FCz

	P8, respectively, according to the current version of the 10-10 system.	
**eeg_data_loss_pct**	"Figure 3. Average (±SD) inoculation event–related potential (iERP) after the first needle contact (at time= 0 milliseconds; vertical line) in 1- to 2-month-olds (n= 13 inoculations from **12 infants**" Since the number of infants analysed is the same with the number of infants studied in 'Methods' section, it is inferred that there is no data loss.	0
**epoch_rej_method**	*"Channels containing movement artifact (defined as **activity exceeding ±100 μV**) or high-frequency muscle activity were removed."*	Objective
**amplitude_threshold**	*"Channels containing movement artifact (defined as **activity exceeding ±100 μV**) or high-frequency muscle activity were removed."*	Activity exceeding plus-minus 100 microV
**clinical_pain_scale**	*"Pain behavior was assessed using the **Modified Behavioral Pain Scale (MBPS)**,..."*	MBPS
**non_eeg_recording**	*"**Infants were video recorded** during the inoculation procedure using a standard handheld camcorder..."* It was not reported in the paper, but it is assumed that the video recording included both facial expression and movement, since MBPS covers the 2 domains. ("*The MBPS is based on 3 parameters (**facial expression, cry, and movements**)..."*)	Video recording of facial expression, Video recording of movement

Marsha Campbell-Yeo (n=1 records)

11. Marsha Campbell-Yeo, Britney Benoit, Aaron Newman, Celeste Johnston, Tim Bardouille, Bonnie Stevens, Arlene Jiang. The influence of skin-to-skin contact on Cortical Activity during Painful procedures in preterm infants in the neonatal intensive care unit (iCAP mini): study protocol for a randomized control trial. Trials. 2022;23(1):512. doi:10.1186/s13063-022-06424-4

Variable	***Data from publication***	***Data summary for review***
**publication_year**	2022	2022
**data_country**	*"The study will be conducted in the NICU of the IWK Health in **Halifax, Nova Scotia**, a 40-bed tertiary level referral unit."*	Canada

Mohammad Reza Daliri (n=1 records)

51. Reyhane Shafiee, Mohammad Reza Daliri. Decoding of pain during heel lancing in human neonates with EEG signal and machine learning approach. Sci Rep. 2024;14(1):31244. doi:10.1038/s41598-024-82631-0

Variable	Data from publication	Data summary for review
**publication_year**		2024
**data_country**	*"In this research, we used the EEG signal of 112 neonates (52 females; 29–47 weeks corrected age, median 36 weeks + 5 days) aged between 0.5 and 96 days, who were recruited from the postnatal, special care, or intensive care wards at the* ***Elizabeth Garrett Anderson Obstetric Wing, University College London Hospital (UCLH)*** *from June 2015 to June 2017"*	United Kingdom
**sample_size**	*"In this research, we used the EEG signal of* ***112*** *neonates (52 females; 29–47 weeks corrected age, median 36 weeks + 5 days) aged between 0.5 and 96 days, who were recruited from the postnatal, special care, or intensive care wards at the Elizabeth Garrett Anderson Obstetric Wing, University College London Hospital (UCLH) from June 2015 to June 2017"*	112
**pma_birth_avg**	Not provided	
**pma_study_avg**	*"In this research, we used the EEG signal of 112 neonates (52 females; 29–47 weeks corrected age, median* ***36*** *weeks + 5 days)..."*	36
**sex_male_pct**	*"In this research, we used the EEG signal of 112 neonates (****52 females****; 29–47 weeks corrected age, median 36 weeks + 5 days) aged between 0.5 and 96 days, who were recruited from the postnatal, special care, or intensive care wards at the Elizabeth Garrett Anderson Obstetric Wing, University College London Hospital (UCLH) from June 2015 to June 2017"* Male percentage = (112-52)/112*100% = 53.57%	53.57
**sex_female_pct**	*"In this research, we used the EEG signal of 112 neonates (****52 females****; 29–47 weeks corrected age, median 36 weeks + 5 days) aged between 0.5 and 96 days, who were recruited*	46.43

	from the postnatal, special care, or intensive care wards at the Elizabeth Garrett Anderson Obstetric Wing, University College London Hospital (UCLH) from June 2015 to June 2017" Female percentage = 52/112*100% = 46.43%	
**pain_procedure**	*"The brain activity of these infants was recorded under* ***heel incision using a lancet...****"*	Heel lance
**analgesic_intervention**	Not applicable	
**electrode_placement_method**	Not provided	
**electrode_placement_system**	*"The brain activity of these infants was recorded under heel incision using a lancet and using up to 20 electrodes that were scattered on the head according to* ***the standard of 10–10****..."*	10-10 system
**electrode_positions**	Not provided	
**eeg_data_loss_pct**	Considering that this research only focuses on pain processing, among the 112 available data files, only the data related to 107 neonates, which includes the information of 18 channels (the information of and channels was not available for all neonates), was used. Many of these data lacked PIPP rating for reasons such as incomplete behavioural and physiological information; in some of the data, there was a high movement noise that could not be resolved, so in total, out of 107 subjects, 70 subjects with clear pain levels according to the PIPP criteria and 37 subjects who were subjected to heel incision stimulation, but their pain levels were not known, were used for the pain-free condition. ((112-107)/112).	4.46
**epoch_rej_method**	Not applicable. No epoch rejection method was mentioned in the paper.	
**amplitude_threshold**	Not applicable. No epoch rejection method was mentioned in the paper.	
**clinical_pain_scale**	*"...by using the Premature Infant Pain Profile (****PIPP****), it was determined that the level of pain in infants is 21 according to the parameters in this criterion"*	PIPP/PIPP-R
**non_eeg_recording**	Not applicable	

Nathalie Maitre (n=2 records)

27. Lance Relland, Caitlin Kjeldsen, Arnaud Jeanvoine, Lelia Emery, Kathleen Adderley, Rachelle Srinivas, Maeve McLoughlin, Nathalie Maitre. Vibration-based mitigation of noxious-evoked responses to skin puncture in neonates and infants: a randomised controlled trial. Archives of Disease in Childhood - Fetal and Neonatal Edition. Published online March 13, 2024. doi:10.1136/archdischild-2023-326588

Variable	*Data from publication*	*Data summary for review*
**publication_year**	2024	2024
**data_country**	*"Ethics approval This study involved human participants and was approved by* ***Nationwide Children's Hospital*** *Institutional Review Board"*	United States of America
**sample_size**	*"We enrolled 134 infants..."*	134
**pma_birth_avg**	Table 1. This table gives information on the 81 participants that were analysed, not all the participants with EEG recorded.*" Age in weeks, median (IQR) Gestational* ***36.4*** *(33.9– 37.8)* ***35.3*** *(30.1–37.0)".* We calculate the average of the 2 groups = (36.4+35.3)/2 = 35.85	35.85
**pma_study_avg**	Table 1. This table gives information on the 81 participants that were analysed, not all the participants with EEG recorded.*" Age in weeks, median (IQR) Postmenstrual* ***39.5*** *(38.1–40.6)* ***39.1*** *(37.6–40.6)".* We calculate the average of the 2 groups = (39.5+39.1)/2 = 39.3	39.3
**sex_male_pct**	Table 1. This table gives information on the 81 participants that were analysed, not all the participants with EEG recorded. "*Male sex, n (%) 23* ***(57.5)*** *27* ***(65.9)****"* We calculate the average of the percentage reported for the 2 groups = (57.5+65.9)/2 = 61.7%	61.7
**sex_female_pct**	Table 1. This table gives information on the 81 participants that were analysed, not all the participants with EEG recorded. "*Male sex, n (%)* ***23*** *(57.5)* ***27*** *(65.9)"* We calculate the percentage of female from the number of males reported for the 2 groups = (81-(23+27))/81* 100 = 38.27% =38.3%	38.3
**pain_procedure**	*"In the current prospective randomised controlled trial, we used time- locked EEG methodologies to test the hypothesis that a concomitant non- noxious vibratory stimulus is able to mitigate noxious-evoked responses to* ***heel lance*** *during routine blood sampling."*	Heel lance
**analgesic_intervention**	*"****Vibration****- based mitigation of noxious- evoked responses to skin puncture in neonates and infants: a randomised controlled trial"*	Vibration

**electrode_placement_method**	***"An appropriately sized (based on head circumference) 128- channel EEG soft- sponge net*** *(Geodesic Sensor Net, EGI, Eugene, Oregon, USA) soaked in a warm saline solution was applied to the infant's head."*	Cap
**electrode_placement_system**	*"An appropriately sized (based on head circumference)* ***128- channel EEG*** *soft- sponge net (****Geodesic*** *Sensor Net, EGI, Eugene, Oregon, USA) soaked in a warm saline solution was applied to the infant's head."*	128-channel Geodesic system
**electrode_positions**	*"The midline (****Cz****) electrode was used as the reference"*	Cz
**eeg_data_loss_pct**	Figure 2: Number of participants "*excluded due to artefact*" = 27+26 = 53. To get the percentage, we divided 53 with the sample size (134) = 39.55%	39.55
**epoch_rej_method**	*"Outlier segments contaminated by electrical, motion, and/or ocular artefacts were excluded using* ***Net Station software*** *in tandem with* ***customised computations*** *written using Python software and verified by* ***manual review****."*	Mixed
**amplitude_threshold**	Not provided	
**clinical_pain_scale**	Figure 1D. The facial coding system looks like NFCS, but only 7 among 10 were used.	
**non_eeg_recording**	*"Components of facial coding outlined in figure 1D were assessed as present or absent within a 10 s* ***video clip of the patient at rest*** *(i.e., before any unswaddling/skin prep), after the onset of vibration (if applicable),* ***and following skin puncture****."*	Video recording of facial expression, Video recording of sleep and behavioral state

43. Nathalie Maitre, Ann Stark, Carrie McCoy Menser, Olena Chorna, Daniel France, Alexandra Key, Ken Wilkens, Melissa Moore-Clingenpeel, Don Wilkes, Stephen Bruehl. Cry presence and amplitude do not reflect cortical processing of painful stimuli in newborns with distinct responses to touch or cold. Arch Dis Child Fetal Neonatal Ed. 2017;102(5):F428-F433. doi:10.1136/archdischild-2016-312279

Variable	***Data from publication***	***Data summary for review***
**publication_year**	2017	2017
**data_country**	*"Ethics approval* ***Vanderbilt University*** *Institutional Review Board."*	United States of America
**sample_size**	*"We studied 54 full-term infants (table 1).* ***Data for one participant were excluded due to an insufficient number of artefact-free ERP trials in all conditions****. Of the remaining 53 subjects, all had measurable, artefact-free cortical responses for light touch, 32 for cold and* ***33 for heel stick*** *response."* From this, it is	34

	assumed that EEG recording is available for 33 (analysed)+1 (excluded due to artefact) = 34 babies.	
**pma_birth_avg**	Table 1 is for n=54 babies, so not exclusive to baby with EEG recording during heel lance only. Since no separate demographic data was reported for n=34 babies with EEG data during heel lance, the data from this table was extracted. "*EGA in weeks, median (IQR)* ***39*** *(38, 40)"*	39
**pma_study_avg**	Table 1 is for n=54 babies, so not exclusive to baby with EEG recording during heel lance only. Since no separate demographic data was reported for n=34 babies with EEG data during heel lance, the data from this table was extracted. "*Hours between feeding and heel stick, median (IQR)* ***1.8*** *(0.8, 3.1)"* The unit is converted to weeks as follows: 1.8/24/7 = 0.01 weeks, and added to the average of GA: 0.01+39 = 39.01 weeks.	39.01
**sex_male_pct**	Table 1 is for n=54 babies, so not exclusive to baby with EEG recording during heel lance only. Since no separate demographic data was reported for n=34 babies with EEG data during heel lance, the data from this table was extracted. *"Male sex 25* ***46.2****"*	46.2
**sex_female_pct**	Table 1 is for n=54 babies, so not exclusive to baby with EEG recording during heel lance only. Since no separate demographic data was reported for n=34 babies with EEG data during heel lance, the data from this table was extracted. The female percentage is calculated from the percentage of male reported. *"Male sex 25* ***46.2****"* Female = 100-46.2 = 53.8	53.8
**pain_procedure**	*"Interventions We prospectively studied newborn cortical responses to light touch, cold and* ***heel stick****..."*	Heel lance
**analgesic_intervention**	Not applicable	
**electrode_placement_method**	*"A* ***128-channel EEG*** *soft-sponge net (Geodesic Sensor Net, EGI, Eugene, Oregon, USA) soaked in warm saline was applied to the infant's head."*	Cap
**electrode_placement_system**	*"A* ***128-channel EEG*** *soft-sponge net (****Geodesic*** *Sensor Net, EGI, Eugene, Oregon, USA) soaked in warm saline was applied to the infant's head."*	128-channel Geodesic system
**electrode_positions**	Figure 1, and *"...the midline* ***Cz*** *electrode was used as the reference"*	F3, F4, C3, C4, Cz
**eeg_data_loss_pct**	*"We studied 54 full-term infants (table 1).* ***Data for one participant***	2.94

	were excluded due to an insufficient number of artefact-free ERP trials in all conditions*. Of the remaining 53 subjects, all had measurable, artefact-free cortical responses for light touch, 32 for cold and* ***33 for heel stick*** *response."* From this, it is assumed that EEG recording is available for 33 (analysed)+1 (excluded due to artefact) = 34 babies. So the number of data loss is 1/34 * 100 = 2.94%	
**epoch_rej_method**	*"Segments contaminated by motor or ocular artefacts were excluded using* ***standard algorithms included in NetStation and verified by manual review."***	Mixed
**amplitude_threshold**	Not provided	
**clinical_pain_scale**	Not applicable	
**non_eeg_recording**	*"We recorded vocalisations using a portable high-quality field recorder (TASCAM DR-100) with a unidirectional external microphone located 25 cm from the subject's mouth. Cry samples were digitised using a 32-bit analogue to digital converter at 44.1 kHz sampling rate."*	Acoustical recordings of cry

Paul Castillo (n=1 records)

47. Paul Castillo, Sampsa Vanhatalo, Marit Lundblad, Mats Blennow, Per-Arne Lonnqvist. EEG response to a high volume (1.5 mL/kg) caudal block in infants less than 3 months. Reg Anesth Pain Med. 2023;49(3):163-167. doi:10.1136/rapm-2023-104452

Variable	***Data from publication***	***Data summary for review***
**publication_year**	2023	2023
**data_country**	It was not reported, so it is inferred based on the corresponding author's affiliation.	Sweden
**sample_size**	*"**Eleven** infants less than 3 months of postnatal age and scheduled to undergo hernial repair were recruited."*	11
**pma_birth_avg**	Not provided	
**pma_study_avg**	Not provided	
**sex_male_pct**	Not provided	
**sex_female_pct**	Not provided	
**pain_procedure**	*"Following a 5 min baseline period, a **caudal block** was performed (1.5 mL/kg), whereafter the EEG, hemodynamic, and cerebral near-infrared spectroscopy responses were followed during a 20 min observation period that was divided into four 5 min segments."*	Lumbar puncture
**analgesic_intervention**	*"Anesthetic protocol<br>No premedication was used. Depending on the presence or absence of peripheral venous access, anesthesia was either induced by intravenous **propofol** (3mg/kg) or inhalation of **sevoflurane** (8% in oxygen), followed by obtaining intravenous access. A laryngeal mask airway was inserted, and the anesthesia was subsequently maintained by spontaneous ventilation with pressure support of **sevoflurane** 2.5% in oxygen/air (50% oxygen). The pressure support was adjusted to result in an end-tidal CO2 concentration within the 4.5%–6.0%range."*	Propofol, Sevoflurane
**electrode_placement_method**	Electrode positions were listed and 'cap' was not mentioned, so we assume individual electrodes placement method were used.	Individual electrodes
**electrode_placement_system**	*"EEG electrodes (using nine electrodes according to **the 10–20 standard**) were applied"*	10-20 system
**electrode_positions**	*"Since the EEG signal collected in the operation theater did exhibit variable levels of artifacts, we decided to focus on the EEG channels that are least sensitive to movement artifacts and best described in the literature on infant brain monitoring: frontal and*	F3, F4, P3, P4

	*parietal electrodes in the left and right side (**F3, F4, P3, P4**).”*	
**eeg_data_loss_pct**	“The inclusion was stopped after including **11** of the planned 15 patients... The main finding of the present study was that a high-volume caudal block in 10 out of **11** of the patients produced transient global EEG changes (mainly an increase in delta relative power).” Since the number of participants recruited in the method section and discussed in the discussion section are the same (11), it could be inferred that no data was excluded after EEG recording.	0
**epoch_rej_method**	*“All EEG signals were initially assessed **visually** to identify overall signal quality and the type of background activity.”*	Subjective
**amplitude_threshold**	Not applicable	
**clinical_pain_scale**	Not applicable	
**non_eeg_recording**	*“Hemodynamics and end-tidal CO2 **Heart rate, NIBPs, and end- tidal CO2** remained stable throughout the study period (figure 2). Near-infrared spectroscopy The **NIRS** values varied within the −12 to +8 percentage point range between the baseline value and the 5 min postinjection recording (table 2).”*	Heart rate, End-tidal CO2, NIRS, Blood pressure

Pierre Kuhn (n=1 records)

4. Pierre Kuhn. Multimodal Approach to the Ontogenesis of Nociception in Very Preterm and Term Infants (NOCI-Prem).; 2019. https://clinicaltrials.gov/study/NCT05404594

Variable	*Data from publication*	*Data summary for review*
**publication_year**	*"Study Start (Actual)<br>**2019**-07-17"*	2019
**data_country**	*"This study has 1 location<br>**France**<br>Strasbourg, France, 67000<br>Recruiting<br>Service de Pédiatrie – Hôpital d'Hautepierre"*	France

Rebecca Pillai Riddell (n=1 records)

5. Rebecca Pillai Riddell. Rebooting Infant Pain Assessment: Using Machine Learning to Exponentially Improve Neonatal Intensive Care Unit Practice (BabyAI).; 2020. https://clinicaltrials.gov/study/NCT05579496

Variable	*Data from publication*	*Data summary for review*
**publication_year**	*"Study Start (Actual)<br>**2020**-11-01"*	2020
**data_country**	*"This study has 2 locations<br>**Canada**<br>Ontario Locations<br>Toronto, Ontario, Canada, M5G 1X5<br>Recruiting<br>Mount Sinai Hospital<br>Contact:<br>Vibhuti Shah, MD<br>416-586-4816<br>vibhuti.shah@sinaihealthsystem.ca<br>Contact:<br>Carol Cheng, MSc<br>416-586-4816<br>carol.cheng@sinaihealthsystem.ca<br>**United Kingdom**<br>No Province Locations<br>London, No Province, United Kingdom, N1 2EP<br>Recruiting<br>University College London Hospital<br>Contact:<br>Judith Meek, MD<br>020 3447 8094 judith.meek@nhs.net<br>Contact:<br>Pureza Laudano-Dray, BScN<br>020 3447 8094 Pureza m.laudiano-dray@ucl.ac.uk"*	Canada, United Kingdom

Rebeccah Slater (n=14 records)

8. Alan Worley, Lorenzo Fabrizi, Stewart Boyd, Rebeccah Slater. Multi-modal pain measurements in infants. J Neurosci Methods. 2012;205(2):252-257. Doi:10.1016/j.jneumeth.2012.01.009

Variable	*Data from publication*	*Data summary for review*
**publication_year**	2012	2012
**data_country**	Based on the authors' affiliations	United Kingdom

10. Lorenzo Fabrizi, Alan Worley, Debbie Patten, Siân Roberts-Holdridge, Laura Cornelissen, Judith Meek, Stewart Boyd, Rebeccah Slater. Electrophysiological Measurements and Analysis of Nociception in Human Infants. JoVE (Journal of Visualized Experiments). 2011;(58):e3118. Doi:10.3791/3118

Variable	*Data from publication*	*Data summary for review*
**publication_year**	2011	2011
**data_country**	Based on the authors' affiliations	United Kingdom

13. Annalisa Hauck, Marianne van der Vaart, Eleri Adams, Luke Baxter, Aomesh Bhatt, Daniel Crankshaw, Amraj Dhami, Ria Evans Fry, Marina Freire, Caroline Hartley, Roshni Mansfield, Simon Marchant, Vaneesha Monk, Fiona Moultrie, Mariska Peck, Shellie Robinson, Jean Yong, Ravi Poorun, Maria Cobo, Rebeccah Slater. Effect of parental touch on relieving acute procedural pain in neonates and parental anxiety (Petal): a multicentre, randomised controlled trial in the UK. The Lancet Child & Adolescent Health. 2024;8(4):259-269. doi:10.1016/S2352-4642(23)00340-1

Variable	*Data from publication*	*Data summary for review*
**publication_year**	2024	2024
**data_country**	"Effect of parental touch on relieving acute procedural pain in neonates and parental anxiety (Petal): a multicentre, randomised controlled trial in the **UK**"	United Kingdom
**sample_size**	"*Figure 1*": *"…received the allocated intervention":* **54**+**54** = 108	108
**pma_birth_avg**	"*Table: Baseline characteristics*": "*Gestational age at birth (weeks)":* (**38.8**+**38.0**)/2=38.4	38.4
**pma_study_avg**	"*Table: Baseline characteristics*": "*Postmenstrual age at time of study (weeks)"*: (**38.9**+**38.4**)/2=38.65	38.65
**sex_male_pct**	"*Table: Baseline characteristics*": "*Sex"*: "*Male*": (**61**+**61**)/2=61	61
**sex_female_pct**	"*Table: Baseline characteristics*": "*Sex"*: "*Female*": (**39**+**39**)/2=39	39
**pain_procedure**	*"Neonates without neurological abnormalities who were born at 35 weeks gestational age or more and required a blood test via a heel lance in the first week of life were randomly assigned (1:1) to receive parental touch for 10 s either before (intervention group) or after (control group) the clinically required **heel lance."***	Heel lance

**analgesic_intervention**	*"Neonates without neurological abnormalities who were born at 35 weeks gestational age or more and required a blood test via a heel lance in the first week of life were randomly assigned (1:1) to receive* ***parental touch*** *for 10 s either before (intervention group) or after (control group) the clinically required heel lance."*	Parental stroke
**electrode_placement_method**	Electrode positions were listed and 'cap' was not mentioned, so we assume individual electrodes placement method were used.	Individual electrodes
**electrode_placement_system**	*"Eight EEG recording electrodes were positioned on the scalp at Cz, CPz, C3, C4, FCz, T3, T4, and Oz according to* ***the modified international 10–20 system****. Reference and ground electrodes were placed at Fz and Fpz, respectively."*	Modified 10-20 system
**electrode_positions**	*"Eight EEG recording electrodes were positioned on the scalp at* ***Cz, CPz, C3, C4, FCz, T3, T4,*** *and* ***Oz*** *according to the modified international 10–20 system. Reference and ground electrodes were placed at* ***Fz*** *and Fpz, respectively."* T3 and T4 were standardised to T7 and T8, respectively, according to the current version of the 10-10 system.	Cz, CPz, C3, C4, FCz, T7, T8, Oz, Fz
**eeg_data_loss_pct**	*"Figure 1: ... 15 excluded from analysis of the primary outcome... 11 due to artefacts... 11 excluded from analysis of the primary outcome due to artefacts."*: (**11**+**11**)/(**54**+**54**)*100=20.37%	20.37
**epoch_rej_method**	*"****Subjective*** *quality assessment of the EEG and vital signs data for artefact detection was performed by two masked investigators, with any discrepancies in assessment resolved by discussion."*	Subjective
**amplitude_threshold**	Not applicable	
**clinical_pain_scale**	*"Secondary outcome measures were* ***Premature Infant Pain Profile-Revised (PIPP-R) score****, development of tachycardia, and parental anxiety score."*	PIPP/PIPP-R
**non_eeg_recording**	"During both sham procedure and clinical heel lance, a **video of the baby's facial expressions** was recorded. The videos were used to **categorise the behavioural state of the babies** before the heel lance using the groups described in the PIPP-R score...vital signs were used to calculate the occurrence of tachycardia in response to the heel lance and to calculate the **heart rate**	Oxygen saturation, Heart rate, Video recording of facial expression, Video recording of sleep and behavioral state

	and **oxygen saturation** components of the PIPP-R score.”	

16. Caroline Hartley, Fiona Moultrie, Deniz Gursul, Amy Hoskin, Eleri Adams, Richard Rogers, Rebeccah Slater. Changing Balance of Spinal Cord Excitability and Nociceptive Brain Activity in Early Human Development. Current Biology. 2016;26(15):1998-2002. doi:10.1016/j.cub.2016.05.054

Variable	***Data from publication***	***Data summary for review***
**publication_year**	2016	2016
**data_country**	*“40 infants were recruited between May 2012 and June 2015 from the Neonatal and Maternity Units of the John Radcliffe Hospital, **Oxford**.”*	United Kingdom
**sample_size**	*“**40** infants were recruited between May 2012 and June 2015 from the Neonatal and Maternity Units of the John Radcliffe Hospital, Oxford.”*	40
**pma_birth_avg**	Table 1: “*Gestational age at birth (weeks)— median (IQR) **34.4** (29.6–40.6)*”	34.4
**pma_study_avg**	Table 1: “*Gestational age at time of study (weeks)—median (IQR) **36.4** (33.3–40.9)*”	36.4
**sex_male_pct**	Table 1: *“Male infants (%) 20 (**50**)”*	50
**sex_female_pct**	Percentage of females is calculated from percentage of males in table 1. *“Male infants (%) 20 (**50**)”* Percentage of females = 100-50 = 50%	50
**pain_procedure**	*“All **heel lances** performed in the study were clinically required as part of the infant's medical care.”*	Heel lance
**analgesic_intervention**	Not applicable	
**electrode_placement_method**	Electrode positions were listed and ‘cap’ was not mentioned, so we assume individual electrodes placement method were used.	Individual electrodes
**electrode_placement_system**	*“Recording electrodes (Ambu Neuroline disposable Ag/AgCl cup electrodes) were positioned at Cz, CPz, C3, C4, FCz, Oz, T3 and T4, according to **the modified international 10/20 electrode placement system**.”*	Modified 10-20 system
**electrode_positions**	*“Recording electrodes (Ambu Neuroline disposable Ag/AgCl cup electrodes) were positioned at **Cz, CPz, C3, C4, FCz, Oz, T3 and T4**, according to the modified international 10/20 electrode placement system. Reference and ground electrodes were placed at **Fz** and on the*	Cz, CPz, C3, C4, FCz, Oz, T7, T8, Fz, Fpz

	*forehead respectively. A reduced electrode montage was applied in 8 infants but activity was always recorded at the Cz electrode. In 10 infants the recordings were acquired with reference to **Fpz** and re-referenced post-acquisition to Fz."* T3, T4 are standardised to T7, T8, respectively, according to the current version of the 10-10 system.	
**eeg_data_loss_pct**	"*A total of **four** infants were rejected from EEG analysis because epochs contained gross movement or signal artifacts (for example, one epoch was rejected due to repetitive artifacts caused by the infant's respiratory support).*" Percentage of data loss = 4/40*100 = 10%	10
**epoch_rej_method**	Not provided. "*A total of four infants were rejected from EEG analysis because epochs contained gross movement or signal artifacts (for example, one epoch was rejected due to repetitive artifacts caused by the infant's respiratory support).*"	
**amplitude_threshold**	Not provided	
**clinical_pain_scale**	Not applicable	
**non_eeg_recording**	*"**EMG** and EEG activity was recorded during a background rest period and during a clinically required heel lance"*	EMG

17. Caroline Hartley, Eugene Duff, Gabrielle Green, Gabriela Schmidt Mellado, Alan Worley, Richard Rogers, Rebeccah Slater. Nociceptive brain activity as a measure of analgesic efficacy in infants. Science Translational Medicine. 2017;9(388):eaah6122. doi:10.1126/scitranslmed.aah6122

Variable	***Data from publication***	***Data summary for review***
**publication_year**	2017	2017
**data_country**	*"Infants were recruited from the Maternity Unit, Special Care Baby Unit, and Neonatal Outpatient Clinics at the John Radcliffe Hospital, **Oxford** University Hospitals National Health Service Foundation Trust."*	United Kingdom
**sample_size**	Table 1. "*Number of infants **18** 14 **12*** 28* 12... The term infants in study 4 were an independent sample of infants not included in the other studies (n = **16**).*" Infants receiving skin-breaking procedure (heel lance) = 18 (study 1) + 12 (study 3) + 16 (independent infants in study 4) = 46	46
**pma_birth_avg**	Table 1: "*Gestational age at birth (weeks) **39.3** (37.5, 41.4) 39.6 (37.2, 41.2) **33.0** (31.9, 36.1) **36.1** (31.7, 40.4) 38.7 (38.0, 39.8)*" Only infants	36.33

	receiving heel lance are included in this data extraction, so only data from study 1, 3, and 4 are included in the averaging of PMA at birth. Note that for study 4 the data presented includes some independent babies. Average PMA at birth = (39.9+33.0+36.1)/3 = 38.2	
**pma_study_avg**	Table 1: "*Gestational age at time of study (weeks)* ***39.9*** *(37.9, 41.7) 39.8 (37.8, 41.3)* ***36.1*** *(35.1, 36.6)* ***38.6*** *(36.4, 40.9) 42.1 (41.1, 43.6)"* Only infants receiving heel lance are included in this data extraction, so only data from study 1, 3, and 4 are included in the averaging of PMA at study. Note that for study 4 the data presented includes some independent babies. Average PMA at study = (39.9+36.1+38.6)/3 = 38.2	38.2
**sex_male_pct**	Table 1: "*Number of males* ***11*** *3* ***7 16*** *5*" Only infants receiving heel lance are included in this data extraction, so only number of males and number of infants from study 1, 3, and 4 are included in the averaging of percentage of males. Note that for study 4 the data presented includes some independent babies. Average percentage of males = (11+7+16)/(18+12+28)*100 = 34/58*100 = 58.62%	58.62
**sex_female_pct**	Calculated from percentage of males (see 'sex_male_pct' section): 100-58.62= 41.38%	41.38
**pain_procedure**	Table 1: "*Applied stimuli* ***Heel lance****, control heel lance, experimental noxious, tactile Experimental noxious, visual, auditory, tactile* ***Heel lance****, control heel lance* ***Heel lance****, control heel lance Experimental noxious*"	Heel lance
**analgesic_intervention**	Not applicable (Analgesic intervention is only on babies receiving non-skin-breaking procedure)	
**electrode_placement_method**	Electrode positions were listed and 'cap' was not mentioned, so we assume individual electrodes placement method were used.	Individual electrodes
**electrode_placement_system**	Not provided	
**electrode_positions**	"*Reference and ground electrodes were placed at* ***Fz*** *and on the fore-head, respectively. For three infants, the reference electrode was placed at* ***Fpz*** *and re-referenced to Fz after acquisition. In studies 1, 2, 3, and 4,*	Cz, CPz, C3, C4, T7, T8, FCz, Oz, Fz, Fpz

	EEG recording electrodes (Ambu Neuroline disposable Ag/AgCl cup electrodes) were placed on the surface of the scalp at ***Cz, CPz, C3, C4, T3, T4, FCz, and Oz****. To limit the study time to about 15 min, a reduced electrode montage, which always included the Cz electrode, was used in all infants in study 5 and in three infants in study 1."* T3, T4 are standardised to T7, T8, respectively, according to the current version of the 10-10 system.	
**eeg_data_loss_pct**	Not provided	
**epoch_rej_method**	Not provided. "*EEG epochs with gross movement artifact were rejected from the analysis*."	
**amplitude_threshold**	Not applicable	
**clinical_pain_scale**	*"Videos were reviewed after the procedures, and the* ***facial expression component of the Premature Infant Pain Profile (PIPP)*** *was calculated in the 30 s after the procedure by identifying the presence of nasolabial furrow, brow bulge, and eye squeeze"* Only the facial expression component of PIPP was calculated.	
**non_eeg_recording**	"***ECG*** *activity was recorded using an ECG electrode (Ambu Neuroline 700 solid gel surface electrodes) that was placed on the chest... Each infant's* ***facial expression*** *was recorded using a* ***video*** *camera for 15 s before and 30 s after the heel lance and control heel lance procedures."*	Heart rate, Video recording of facial expression

18. Caroline Hartley, Fiona Moultrie, Amy Hoskin, Gabrielle Green, Vaneesha Monk, Jennifer Bell, Andrew King, Miranda Buckle, Marianne van der Vaart, Deniz Gursul, Sezgi Goksan, Edmund Juszczak, Jane Norman, Richard Rogers, Chetan Patel, Eleri Adams, Rebeccah Slater. Analgesic efficacy and safety of morphine in the Procedural Pain in Premature Infants (Poppi) study: randomised placebo-controlled trial. The Lancet. 2018;392(10164):2595-2605. doi:10.1016/S0140-6736(18)31813-0

Variable	***Data from publication***	***Data summary for review***
**publication_year**	2018	2018
**data_country**	*"In this single-centre masked trial, 31 infants at the John Radcliffe Hospital, Oxford,* ***UK****, were randomly allocated using a web-based facility with a minimisation algorithm to either 100 µg/kg oral morphine sulphate or placebo 1 h before a clinically required heel lance and retinopathy of prematurity screening examination, on the same occasion."*	United Kingdom

**sample_size**	From figure 1, n=15 assigned morphine and had EEG recorded, n = 15 assigned placebo and had EEG recorded (including 1 excluded from EEG analysis because of artifact, excluding 1 that was withdrawn before study commenced). 15+15 = 30.	30
**pma_birth_avg**	Table 1: *"Gestational age (weeks)*† **28·1** (26·3–30·1) **28·6** (27·9–29·7)"* (28.1+28.6)/2 = 28.35	28.35
**pma_study_avg**	Table 1: *"Gestational age (weeks)*† **34·7** (34·1–35·1) **34·7** (34·1–35·1)"* (34.7+34.7)/2 = 34.7	34.7
**sex_male_pct**	Table 1: "Male sex 12 (**80**%) 8 (**53**%)" (80+53)/2 = 66.5	66.5
**sex_female_pct**	Table 1: *"Female sex 3 (**20%**) 7 (47%)"*(20+47)/2 = 33.5	33.5
**pain_procedure**	*"In this single-centre masked trial, 31 infants at the John Radcliffe Hospital, Oxford, UK, were randomly allocated using a web-based facility with a minimisation algorithm to either 100 µg/kg oral morphine sulphate or placebo 1 h before a clinically required **heel lance** and retinopathy of prematurity screening examination, on the same occasion."*	Heel lance
**analgesic_intervention**	*"In this single-centre masked trial, 31 infants at the John Radcliffe Hospital, Oxford, UK, were randomly allocated using a web-based facility with a minimisation algorithm to either 100 µg/kg oral **morphine sulphate** or placebo 1 h before a clinically required heel lance and retinopathy of prematurity screening examination, on the same occasion."* Figure 1*:* n=15 babies received morphine.	Morphine
**electrode_placement_method**	Electrode positions were listed and 'cap' was not mentioned, so we assume individual electrodes placement method were used.	Individual electrodes
**electrode_placement_system**	*"Eight EEG recording electrodes (Ambu Neuroline disposable Ag/AgCl cup electrodes) were positioned on the scalp at Cz, CPz, C3, C4, FCz, T3, T4 and Oz, according to **the modified international 10-20 System**, with reference and ground electrodes at Fz and the forehead respectively."*	Modified 10-20 system
**electrode_positions**	*"Eight EEG recording electrodes (Ambu Neuroline disposable Ag/AgCl cup electrodes) were positioned on the scalp at **Cz, CPz, C3, C4, FCz, T3, T4 and Oz**, according to the modified*	Cz, CPz, C3, C4, FCz, T7, T8, Oz, Fz

	international 10-20 System, with reference and ground electrodes at ***Fz*** *and the forehead respectively."* T3, T4 are standardised to T7, T8, respectively, according to the current version of the 10-10 system.	
**eeg_data_loss_pct**	Figure 1: "One excluded because of artifact." Percentage of data loss = 1/30*100 = 3.33%.	3.33
**epoch_rej_method**	Not provided. Fig.1:" *One excluded because of artifact*."	
**amplitude_threshold**	Not applicable	
**clinical_pain_scale**	*"Secondary outcome measures were reflex withdrawal and the* ***PIPP-R score*** *after heel lancing"*	PIPP/PIPP-R
**non_eeg_recording**	*"Continuous electronic data capture of* ***heart rate, respiratory rate****, and* ***oxygen saturation*** *began approximately 24 h before the clinical procedure to establish a baseline of clinical stability for every infant…Electroencephalography (EEG) and electromyography (****EMG****) electrodes were then sited, as described in the appendix…Shortly before the clinical procedure, we swaddled the infant (to provide non-pharmacological pain relief), began* ***video monitoring****…"*Supplementary appendix: "***Facial expressions were filmed throughout the clinical procedure****; a clear view of the face was recorded for 15 seconds before and 30 seconds after the heel lance control, the heel lance and the ROP screening."*	EMG, Heart rate, Respiratory rate, Oxygen saturation, Video recording of facial expression

21. Deniz Gursul, Sezgi Goksan, Caroline Hartley, Gabriela Schmidt Mellado, Fiona Moultrie, Amy Hoskin, Eleri Adams, Gareth Hathway, Susannah Walker, Francis McGlone, Rebeccah Slater. Stroking modulates noxious-evoked brain activity in human infants. Current Biology. 2018;28(24):R1380-R1381. doi:10.1016/j.cub.2018.11.014

Variable	*Data from publication*	*Data summary for review*
**publication_year**	2018	2018
**data_country**	Supplemental information: "*Infants were recruited from the Maternity Unit at the John Radcliffe Hospital, Oxford University Hospitals National Health Service Foundation Trust,* ***UK****."*	United Kingdom
**sample_size**	Supplemental information: *"We recruited* ***20*** *infants (inflated sample size to account for missing data) to receive CT-optimal touch before a clinically necessary heel lance. 4 infants were removed from the analysis due to artefacts.* ***16*** *age-*	36

	matched infants, who had not received touch stimulation prior to heel lancing, were selected from a group of infants whose heel lance responses had previously been recorded as part of other research studies." Sample size of babies receiving heel lance = 20+16 = 36.	
**pma_birth_avg**	Not provided	
**pma_study_avg**	Supplemental information: "*Table of infant demographics: Gestational age at study (weeks) 40.0 (39.0, 41.0)* ***39.9*** *(38.0, 41.7)* ***39.8*** *(37.9, 41.3)*" There is no skin-breaking procedure involved in study 1, so only data from study 2 is recorded in this data extraction. This table only consists of information for babies who were analysed. Average PMA at study = (39.9+39.8)/2 = 39.85%.	39.85
**sex_male_pct**	Supplemental information: *"Table of infant demographics: Number of males 14 (46.7 %) 10 (**62.5** %) 5 (**31.3** %)"* There is no skin-breaking procedure involved in study 1, so only data from study 2 is recorded in this data extraction. This table only consists of information for babies who were analysed. Average percentage of males = (62.5+31.3)/2 = 46.9%	46.9
**sex_female_pct**	Percentage of females is calculated from percentage of males (see section 'sex_male_pct'): 100-46.9 = 53.1%	53.1
**pain_procedure**	*"We demonstrate that stroking (at 3 cm/s) prior to an experimental noxious stimulus or clinical* ***heel lance*** *can attenuate noxious-evoked brain activity in infants."*	Heel lance
**analgesic_intervention**	Supplemental information: "*In 20 infants,* ***CT-optimal touch*** *(3 cm/s) was applied for approximately 10 seconds prior to the heel lance. The duration of the* ***brush*** *stimulation was increased to 10 seconds as this was consistent with previous studies*"	Brush stroking
**electrode_placement_method**	Electrode positions were listed and 'cap' was not mentioned, so we assume individual electrodes placement method were used.	Individual electrodes
**electrode_placement_system**	Supplemental information: *"EEG electrodes were placed at Cz, CPz, C3, C4, Oz, FCz, T3, T4, with*	Modified 10-20 system

	reference at Fz and ground at FPz, according to ***the modified international 10-20 system.*** *The reference electrode was positioned at Fz"* *system. The reference electrode was positioned at Fz"*	
**electrode_positions**	Supplemental information: *"EEG electrodes were placed at* ***Cz, CPz, C3, C4, Oz, FCz, T3, T4,*** *with reference at* ***Fz*** *and ground at FPz, according to the modified international 10-20 system. The reference electrode was positioned at* ***Fz****"*. T3, T4 are standardised to T7, T8, respectively, according to the current version of the 10-10 system.	Cz, CPz, C3, C4, Oz, FCz, T7, T8, Fz
**eeg_data_loss_pct**	Supplemental information: *"We recruited 20 infants (inflated sample size to account for missing data) to receive CT-optimal touch before a clinically necessary heel lance.* ***4*** *infants were removed from the analysis due to artefacts. 16 age-matched infants, who had not received touch stimulation prior to heel lancing, were selected from a group of infants whose heel lance responses had previously been recorded as part of other research studies."* Percentage of data loss = 4/(20+16)*100 = 4/36*100 = 11.11%	11.11
**epoch_rej_method**	Not provided. Supplemental information: *"Individual epochs were rejected* ***if they contained artefact such as gross movement artefact****, or* ***if there was movement in the baseline period.****"*	
**amplitude_threshold**	Not provided	
**clinical_pain_scale**	Supplemental information: *"In Study 2, two blinded trained observers assessed* ***the duration of nasolabial furrow, brow bulge and eye squeeze (according to the Premature Infant Pain Profile-Revised (PIPP-R) i****n the 30 seconds after the heel lance retrospectively from the video recordings."* Only facial expression component of PIPP-R that was recorded.	
**non_eeg_recording**	Supplemental information: *"****EMG*** *was recorded with bipolar electrodes placed on the biceps femoris of the leg... In Study 2* ***facial expressions***	EMG, Video recording of facial expression

	were recorded using a video camera...”	

24. Gabrielle Green, Caroline Hartley, Amy Hoskin, Eugene Duff, Adam Shriver, Dominic Wilkinson, Eleri Adams, Richard Rogers, Fiona Moultrie, Rebeccah Slater. Behavioural discrimination of noxious stimuli in infants is dependent on brain maturation. PAIN. 2019;160(2):493. doi:10.1097/j.pain.0000000000001425

Variable	***Data from publication***	***Data summary for review***
**publication_year**	2019	2019
**data_country**	*“Between April 2012 and May 2017, a total of 122 infants were recruited from the Newborn Care Unit and Maternity wards of the John Radcliffe Hospital, Oxford University Hospital NHS Foundation Trust, Oxford,* ***United Kingdom****.”*	United Kingdom
**sample_size**	*“...****49*** *infants were included in an analysis of evoked brain activity across the preterm period.”*	49
**pma_birth_avg**	Table 1. “*Gestational age at birth (wk)*” Note: This table represents all infants in the study, including infants without EEG data.	31.9
**pma_study_avg**	Table 1. “*Gestational age at time of study (wk)*” Note: This table represents all infants in the study, including infants without EEG data.	36.5
**sex_male_pct**	Table 1. “*Male infants (%)*” Note: This table represents all infants in the study, including infants without EEG data.	57
**sex_female_pct**	Calculated from the percentage of male infants in Table 1. 100%-57%=	43
**pain_procedure**	*“2.2. Experimental procedures 2.2.1.* ***Heel lancing*** *and control heel lance”*	Heel lance
**analgesic_intervention**	Not applicable	
**electrode_placement_method**	Electrode positions were listed and ‘cap’ was not mentioned, so we assume individual electrodes placement method were used.	Individual electrodes
**electrode_placement_system**	Not provided	
**electrode_positions**	*“EEG was recorded at the* ***Cz, CPz, C3, C4, FCz, Oz, T3, and T4*** *electrode sites, with the reference electrode at* ***Fz*** *and a ground electrode on the forehead. In 11 infants, EEG was recorded at Cz, CPz, C3, and C4 only.”* T3, T4 are standardised to T7, T8, respectively, according to the current version of the 10-10 system.	Cz, CPz, C3, C4, FCz, Oz, T7, T8, Fz
**eeg_data_loss_pct**	*“In the subset of 46 infants who had both facial expression and brain activity recorded,* ***44*** *infants had facial expression and EEG activity recorded in response to both the control heel lance and the heel lance without artefact.”* Data loss = (46-44)/49*100= 4.08%. Note: the	4.08

	denominator is 49 according to the number of infants with EEG recorded.	
**epoch_rej_method**	Not provided. *"Individual EEG channels contaminated with artefacts, such as movement artefact, were removed from the analysis."*	
**amplitude_threshold**	Not provided	
**clinical_pain_scale**	Baseline behavioural state and facial expression part of PIPP-R. *"**The baseline behavioural state was scored** in the 15 seconds preceding the control lance and again in the 15 seconds preceding the heel lance. A score between 0 and 3 was given according to whether the infant was active and awake, quiet and awake, active and asleep, or quiet and asleep, respectively, **as per the Premature Infant Pain Profile—Revised (PIPP-R)...**Each facial expression was also taken individually to calculate a **facial expression score using the facial component of the PIPP/PIPP-R**"*	
**non_eeg_recording**	*"A **video camera was used to record facial expressions** throughout the procedure for post hoc analysis."*	Video recording of facial expression

37. Maria Cobo, Caroline Hartley, Deniz Gursul, Foteini Andritsou, Marianne van der Vaart, Gabriela Schmidt Mellado, Luke Baxter, Eugene Duff, Miranda Buckle, Ria Evans Fry, Gabrielle Green, Amy Hoskin, Richard Rogers, Eleri Adams, Fiona Moultrie, Rebeccah Slater. Quantifying noxious-evoked baseline sensitivity in neonates to optimise analgesic trials. Ploner M, Büchel C, Tibboel D, Ploner M, eds. eLife. 2021;10:e65266. doi:10.7554/eLife.65266

Variable	***Data from publication***	***Data summary for review***
**publication_year**	2021	2021
**data_country**	*"The participants were recruited from the Maternity Unit and Newborn Care Unit at the John Radcliffe Hospital, Oxford University Hospitals National Health Service Foundation Trust, Oxford, **UK**."*	United Kingdom
**sample_size**	*Study 1: Characterising individual noxious-evoked baseline sensitivity using brain activity in neonates The aim of this study was to investigate the relationship between noxious-evoked brain activity in response to experimental stimuli and clinically required **heel lance** within-subjects in a group of term neonates... We identified **nine** neonates" "Study 3: Testing the paradigm: a non-pharmacological pain-relieving intervention study The aim of this study was to test the noxious-evoked baseline sensitivity paradigm using a gentle*	81

	touch intervention of known effect in reducing the noxious-evoked brain activity following a clinically required ***heel lance****...A total of* ***40*** *neonates aged 35–42 weeks' postmenstrual age (PMA) were prospectively recruited to the study.*"<br>*"Study 4: A pharmacological analgesic study Premature-born neonates aged 33–43 weeks' PMA and due to receive* ***immunisations*** *as inpatients in the neonatal unit were recruited for this study... A total of* ***16*** *neonates (Control Group, Figure 5A) were recruited to the study before the clinical practice guidelines were updated in our local neonatal unit to administer paracetamol 1 hr prior to the MenB vaccine. Following the guideline change,* ***16*** *neonates were recruited (Intervention Group)"*<br>Total sample size of babies receiving skin-breaking procedures = 9+40+16+16= 81	
**pma_birth_avg**	Table 1. 'Template validation' group is not included in the averaging because does not consist of babies receiving skin-breaking procedures.<br>*"Gestational age (GA) at birth (weeks)* ***40.7*** *(40.3, 41)* ***40*** *(37.1, 40.7)* ***39.1*** *(37.1, 40.6)* ***27.6*** *(25.6, 28.8)* ***27.3*** *(26.3, 28.3) 40.6 (40, 41)"*<br>Average PMA at birth = (40.7+40+39.1+27.6+27.3)/5 = 34.94	34.94
**pma_study_avg**	Table 1. 'Template validation' group is not included in the averaging because does not consist of babies receiving skin-breaking procedures.<br>*"Postmenstrual age (PMA) at time of study (weeks)* ***41*** *(40.9, 41.7)* ***40.5*** *(37.6, 40.9)* ***39.5*** *(37.8, 41.1)* ***38*** *(37.2, 39.4)* ***37.2*** *(36.2, 38.1) 40.7 (40.1, 41.4)"*<br>Average PMA at study = (41+40.5+39.5+38+37.2)/5 = 39.24	39.24
**sex_male_pct**	Table 1. 'Template validation' group is not included in the averaging because does not consist of babies receiving skin-breaking procedures. There seems to be a mistake in percentage calculation for 'Sex Male' and 'Sex Female' of 'Study 4 - Intervention group': male should be 64% from the number of males provided (9 out of 14), and female should be 36% (5 out of 14). The total male percentage is calculated	55.26

	from the number (not the percentages) provided: *“Male **4** (44) **11** (61) **9** (45) **9** (60) **9** (36) 12 (75)”* Average percentage of males = (4+11+9+9+9) / (9+18+20+15+14) *100 = 42/76*100 = 55.26	
**sex_female_pct**	Table 1. ‘Template validation’ group is not included in the averaging because does not consist of babies receiving skin-breaking procedures. There seems to be a mistake in percentage calculation for ‘Sex Male’ and ‘Sex Female’ of ‘Study 4 - Intervention group’: male should be 64% from the number of males provided (9 out of 14), and female should be 36% (5 out of 14). The total male percentage is calculated from the number (not the percentages) provided: *“Female **5** (56) 7 (39) **11** (55) **6** (40) **5** (64) 4 (25)”* Average percentage of females = (5+7+11+6+5) / (9+18+20+15+14) *100 = 34/76*100 = 44.74	44.74
**pain_procedure**	*“Study 1: Characterising individual noxious-evoked baseline sensitivity using brain activity in neonates The aim of this study was to investigate the relationship between noxious-evoked brain activity in response to experimental stimuli and clinically required **heel lance** within-subjects in a group of term neonates.” “Study 3: Testing the paradigm: a non-pharmacological pain-relieving intervention study The aim of this study was to test the noxious-evoked baseline sensitivity paradigm using a gentle touch intervention of known effect in reducing the noxious-evoked brain activity following a clinically required **heel lance**” “Study 4: A pharmacological analgesic study Premature-born neonates aged 33–43 weeks’ PMA and due to receive **immunisations** as inpatients in the neonatal unit were recruited for this study”*	Heel lance, Immunization
**analgesic_intervention**	*“Study 3: Testing the paradigm: a non-pharmacological pain-relieving intervention study The aim of this study was to test the noxious-evoked baseline sensitivity paradigm using a **gentle touch intervention** of known effect in reducing the noxious-evoked brain activity following a*	Brush stroking, Paracetamol

	clinically required heel lance... The gentle touch stimulus was provided by a ***brush*** *stimulator"* *"Study 4: A pharmacological analgesic study The average time between* ***paracetamol*** *administration and immunisation in neonates in the Intervention Group was 79 min"*	
**electrode_placement_method**	Electrode positions were listed and 'cap' was not mentioned, so we assume individual electrodes placement method were used.	Individual electrodes
**electrode_placement_system**	*"EEG was recorded from eight locations on the scalp (Cz, CPz, C3, C4, Oz, FCz, T3, T4), with reference at Fz and ground at Fpz (forehead) according to* ***the modified international 10–20 system."***	Modified 10-20 system
**electrode_positions**	*"EEG was recorded from eight locations on the scalp (****Cz, CPz, C3, C4, Oz, FCz, T3, T4****), with reference at* ***Fz*** *and ground at Fpz (forehead) according to the modified international 10–20 system."* T3, T4 are standardised to T7, T8, respectively, according to the current version of the 10-10 system.	Cz, CPz, C3, C4, Oz, FCz, T7, T8, Fz
**eeg_data_loss_pct**	Study 1: Not provided. Study 3: 5 (*"EEG responses were rejected for gross movement artefacts. Following removal of neonates whose lances recording were rejected (n =* ***5****)..."*) Study 4: unclear. So the total percentage of data loss is unclear.	
**epoch_rej_method**	Not provided. Method for defining artefact was not reported. *"Epochs were rejected if they contained gross movement artefact."*	
**amplitude_threshold**	Not provided	
**clinical_pain_scale**	Not applicable	
**non_eeg_recording**	*"In Study 3, surface* ***EMG*** *was recorded from the limb ipsilateral to the site of stimulation."*	EMG

38. Maria Cobo, Gabrielle Green, Foteini Andritsou, Luke Baxter, Ria Evans Fry, Annika Grabbe, Deniz Gursul, Amy Hoskin, Gabriela Schmidt Mellado, Marianne van der Vaart, Eleri Adams, Aomesh Bhatt, Franziska Denk, Caroline Hartley, Rebeccah Slater. Early life inflammation is associated with spinal cord excitability and nociceptive sensitivity in human infants. Nat Commun. 2022;13(1):3943. doi:10.1038/s41467-022-31505-y

Variable	***Data from publication***	***Data summary for review***
**publication_year**	2022	2022
**data_country**	*"A total of 65 term neonates were recruited for our hypothesis-testing study from the Newborn Care Unit and Maternity wards of the John Radcliffe*	United Kingdom

	Hospital (Oxford University Hospitals NHS Foundation Trust, Oxford, ***United Kingdom****)"*	
**sample_size**	*"A total of 65 term neonates were recruited for our hypothesis-testing study from the Newborn Care Unit and Maternity wards of the John Radcliffe Hospital (Oxford University Hospitals NHS Foundation Trust, Oxford, United Kingdom) between September 2014 and November 2019 (Supplementary Figure 2 shows the study profile). Data acquisition was completed in* ***61*** *studies... The follow-up exploratory study included 20 participants (2 from the hypothesis-testing study cohort and* ***18*** *from an independent sample)..."* Sample size = 61+18 = 79	79
**pma_birth_avg**	Table 1: *"Gestational age (GA) at birth (weeks)* ***39.9*** *(37.8, 40.7)* ***40*** *(39.2, 41)* ***38.4*** *(36.7, 41)* ***40.4*** *(39.9, 40.7)"* Average PMA at birth = (39.9+40+38.4+40.4)/4 = 39.68	39.68
**pma_study_avg**	Table 1: *"Postmenstrual age (PMA) at the time of the study (weeks)* ***40.1*** *(38, 40.9)* ***40.1*** *(39.4, 41)* ***39.1*** *(37.4, 41.8)* ***41.1*** *(40.5, 41.4)"* Average PMA at study = (40.1+40.1+39.1+41.1)/4 = 40.1	40.1
**sex_male_pct**	The total male percentage is calculated from the number (not the percentages) provided. Table 1: *"Male* ***23*** *(61)* ***13*** *(57)* ***2*** *(25)* ***8*** *(67)"* Average percentage of males = (23+13+2+8) / (38+23+8+12)*100 = 46/81*100 = 56.79%	56.79
**sex_female_pct**	The total male percentage is calculated from the number (not the percentages) provided. Table 1: *"Female* ***15*** *(39)* ***10*** *(43)* ***6*** *(75)* ***4*** *(33)"* Average percentage of females = (15+10+6+4) / (38+23+8+12)*100 = 35/81*100 = 43.21	43.21
**pain_procedure**	*"Neonates were only studied if a* ***heel lance*** *was necessary as part of their clinical care to assess their CRP levels or for routine screening."*	Heel lance
**analgesic_intervention**	Not applicable	
**electrode_placement_method**	Electrode positions were listed and 'cap' was not mentioned, so we assume individual electrodes placement method were used.	Individual electrodes

**electrode_placement_system**	*"Electroencephalography (EEG) was recorded from eight electrode sites (Cz, CPz, C3, C4, Oz, FCz, T3, T4), according to the **modified international 10–20 system** with reference at Fz and ground at Fpz."*	Modified 10-20 system
**electrode_positions**	*"Electroencephalography (EEG) was recorded from eight electrode sites (**Cz, CPz, C3, C4, Oz, FCz, T3, T4**), according to the modified international 10–20 system with reference at **Fz** and ground at Fpz."* T3, T4 are standardised to T7, T8, respectively, according to the current version of the 10-10 system.	Cz, CPz, C3, C4, Oz, FCz, T7, T8, Fz
**eeg_data_loss_pct**	*"Following rejections, a total of 51 participants were included in the noxious-evoked EEG data analysis (**6/38 traces with artefact** were rejected from the Neonatal Control Group and 4/23 traces—**3 with artefact** and 1 due to technical failure—were rejected from the Neonatal Inflammation Group, Supplementary Figure 2). The exploratory study EEG data were processed and analysed using the same methodology and 20 participants (Neonatal Antibiotic Treatment Group: n = 12, Neonatal Antibiotic Control Group: n = 8) were included in the final noxious-evoked EEG data analysis."* Percentage of data loss = (6+3)/79 (see 'sample size' section) *100 = 11.39%	11.39
**epoch_rej_method**	Not provided. "*Epochs were rejected if they contained gross movement artefact.*"	
**amplitude_threshold**	Not applicable	
**clinical_pain_scale**	"*A **PIPP-R** score, which combines beha-vioural and physiological measures and contextual factors, was calculated in response to the stimuli for all the participants in the hypothesis-testing study."*	PIPP/PIPP-R
**non_eeg_recording**	*"Surface electromyography (**EMG**) was recorded using bipolar EMG electrodes (Ambu Neuroline 700 solid gel surface electrodes) placed on the bicep femoris muscles from both legs... The*	EMG, Video recording of facial expression, Heart rate, Oxygen saturation

	neonates' behavioural responses to the stimuli were evaluated by ***recording their facial expressions with a handheld camera****...* ***Oxygen saturation*** *and* ***heart rate*** *were acquired with a pulse oximeter and ECG"*	

41. Marianne van der Vaart, Caroline Hartley, Luke Baxter, Gabriela Schmidt Mellado, Foteini Andritsou, Maria Cobo, Ria Evans Fry, Eleri Adams, Sean Fitzgibbon, Rebeccah Slater. Premature infants display discriminable behavioral, physiological, and brain responses to noxious and nonnoxious stimuli. Cerebral Cortex. 2022;32(17):3799-3815. doi:10.1093/cercor/bhab449

Variable	***Data from publication***	***Data summary for review***
**publication_year**	2022	2022
**data_country**	*"The Oxford Dataset Participants and Research Governance Infants were selected from a database of all data previously recorded by our research group between 2012 and 2021 at the John Radcliffe Hospital, Oxford University Hospitals NHS Foundation Trust, Oxford,* ***UK****... The UCL study was approved by the NHS Health Research Authority (****London****—Surrey Borders) and conformed to the declaration of Helsinki"*	United Kingdom
**sample_size**	*"A total of* ***144*** *infants were included in this study in three datasets"*	144
**pma_birth_avg**	Table 1: "*Gestational age at birth (weeks)* ***34.3*** *(29.2–37.2)* ***34.1*** *(30.2–36.7)* ***34.3*** *(31.0–36.3)*" Average PMA at birth = (34.3+34.1+34.3)/3 = 34.23	34.23
**pma_study_avg**	Table 1: *"PMA at test occasion (weeks)* ***35.3*** *(32.2–37.6)* ***34.9*** *(32.4–37.4)* ***35.6*** *(33.0–37.1)"* Average PMA at study = (35.3+34.9+35.6)/3 = 35.27	35.27
**sex_male_pct**	Table 1: *"Male* ***26*** *(55)* ***13*** *(57)* ***36*** *(49)"* Average percentage of males = (26+13+36) / (47+23+74) *100 = 75/144*100 = 52.08	52.08
**sex_female_pct**	Table 1: *"Female* ***21*** *(45)* ***10*** *(43)* ***38*** *(51)"* Average percentage of females = (21+10+38) / (47+23+74) *100 = 69/144*100= 47.92	47.92
**pain_procedure**	<u>Oxford</u>: *"All infants were studied during a noxious stimulus, a clinically required* ***heel lance*** *performed for blood sampling, and a nonnoxious stimulus..."* <u>UCL</u>: *"The dataset from Jones and coworkers, which is available*	Heel lance

	on request through the UK Data Service (Jones et al. 2018a, 2018b), contains anonymized EEG recordings, ECG recordings, and facial expression scores for 112 infants during a ***heel lance****, control heel lance, and auditory stimulus."*	
**analgesic_intervention**	Not applicable	
**electrode_placement_method**	Oxford: Electrode positions were listed and 'cap' was not mentioned, so we assume individual electrodes placement method were used.<br>UCL: "*Further details on research gover- nance and recording methods can be found in the associ- ated publication (Jones et al. 2018b)."* From the referred paper (doi:10.1038/sdata.2018.248): Electrode positions were listed and 'cap' was not mentioned, so we assume individual electrodes placement method were used.	Individual electrodes
**electrode_placement_system**	Oxford: *"Activity was recorded at Cz and 3–20 other electrodes with the reference electrode at Fz and a ground elec- trode at FPz/forehead according to* ***the international 10– 20 system.****"*<br>UCL: "*Further details on research gover- nance and recording methods can be found in the associ- ated publication (Jones et al. 2018b)."* From the referred paper (doi:10.1038/sdata.2018.248): *"Recording electrodes were positioned according to a* ***modified international 10/10 electrode placement system****..."*	10-20 system, Modified 10-10 system
**electrode_positions**	Oxford: *"Activity was recorded at* ***Cz*** *and 3–20 other electrodes with the reference electrode at* ***Fz*** *and a ground elec- trode at FPz/forehead according to the international 10– 20 system. In two infants, data were recorded with a reference electrode at* ***FPz*** *and re- referenced to Fz during analysis."*<br>UCL: "*Further details on research gover- nance and recording methods can be found in the associ- ated publication (Jones et al. 2018b)."* From the referred paper (doi:10.1038/sdata.2018.248): *"...overlying primary visual (****O1, O2****), primary auditory (****T7, T8****),*	O1, O2, T7, T8, F7, F3, F4, FCz, F8, P7, P8, TP9, TP10, POz, C3, Cz, C4, CP3, CPz, CP4, Fz, Fpz

	*association (**F7, F3, F4, FCz, F8, P7, P8, TP9, TP10, POz**), and somatosensory cortices (**C3, Cz, C4, CP3, CPz, CP4**). The reference electrode was placed at **Fz** and the ground electrode at either FC1 or FC2 (depending on the position of the infant)."*	
**eeg_data_loss_pct**	Table 4*: "EEG ERSP analysis (4 s poststimulus)<br>3/47 heel lances excluded due to movement artifact occurring after 1 s<br>1/23 heel lances excluded due to movement artifact occurring after 1 s<br>Not applicable: only 2 s of poststimulus EEG data available"* Percentage of data loss = (3+1)/(47+23) *100 = 4/144*100 = 2.78%.	2.78
**epoch_rej_method**	*"Epochs were **visually inspected** and rejected if any artifact was present"*	Subjective
**amplitude_threshold**	Not applicable	
**clinical_pain_scale**	Oxford: Not applicable. UCL: "*Further details on research gover-nance and recording methods can be found in the associ-ated publication (Jones et al. 2018b)."* From the referred paper (doi:10.1038/sdata.2018.248): *"This data is linked to pain-related behaviour (facial expression), physiology (heart rate, oxygenation) and a composite clinical score (**Premature Infant Pain Profile, PIPP**). "*	PIPP/PIPP-R
**non_eeg_recording**	Oxford: *"Electrocardiogram (**ECG**) was recorded with an electrode on the chest which was referenced to the EEG reference electrode. Electromyogram (**EMG**) was recorded... Infants' **facial expressions were recorded using a video camera f**or 15 s before and until 30 s after the heel lance and control heel lance"*<br>UCL: *"The dataset from Jones and coworkers, which is available on request through the UK Data Service (Jones et al. 2018a, 2018b), contains anonymized EEG recordings, **ECG** recordings, and **facial expression scores** for 112 infants during a heel lance, control heel lance, and auditory stimulus."* "*Further details on research gover-*	Video recording of facial expression, EMG, Heart rate, Oxygen saturation, Respiratory rate

	nance and recording methods can be found in the associ-ated publication (Jones et al. 2018b).” From the referred paper (doi:10.1038/sdata.2018.248): *“This data is linked to pain-related behaviour (**facial expression**), physiology (**heart rate, oxygenation**) and a composite clinical score (Premature Infant Pain Profile, PIPP)... To determine the PIPP score following the stimuli, the infant’s pulse, blood oxygen saturation and facial expression were monitored using a pulse oximeter and a **video camera**...EEG was recorded according to clinical standards by an experienced clinical scientist. Clinical guidelines suggest recording ECG and **respiratory data** with the EEG.”*	

48. Rebeccah Slater, Alan Worley, Lorenzo Fabrizi, Siân Roberts-Holdridge, Judith Meek, Stewart Boyd, Maria Fitzgerald. Evoked potentials generated by noxious stimulation in the human infant brain. European Journal of Pain. 2010;14(3):321-326. doi:10.1016/j.ejpain.2009.05.005

Variable	***Data from publication***	***Data summary for review***
**publication_year**	2010	2010
**data_country**	*“Twelve infants, 35–39 weeks PMA, recruited from the special care baby unit at the **Elizabeth Garrett Anderson and Obstetric Hospital** participated in the study.”*	United Kingdom
**sample_size**	*“**Twelve** infants, 35–39 weeks PMA, recruited from the special care baby unit at the Elizabeth Garrett Anderson and Obstetric Hospital participated in the study.”*	12
**pma_birth_avg**	Table 1: *“Mean PMA at birth (weeks) **36.4** (1.4); range 34.7–39.0”*	36.4
**pma_study_avg**	Table 1: *“Mean PMA at time of study (weeks) **37.3** (1.2); range 35.9–39.7”*	37.3
**sex_male_pct**	Table 1: “*No. of males 8*” Percentage of males = 8/12 *100 = 66.67%	66.67
**sex_female_pct**	Table 1: “*No. of males 8*” Percentage of females = (12-8)/12 *100 = 33.33%	33.33
**pain_procedure**	*“Here, using a novel approach to time-lock an EEG recording to a clinically required **heel lance**, we show the presence of a distinct nociceptive-specific potential in newborn infants (35–39 weeks postmenstrual age).”*	Heel lance
**analgesic_intervention**	Not applicable	
**electrode_placement_method**	Electrode positions were listed and ‘cap’ was not mentioned, so we assume individual electrodes placement method were used.	Individual electrodes

**electrode_placement_system**	*"Seventeen recording electrodes (Ambu Neuroline disposable Ag/AgCl cup electrodes) were positioned according to* ***the modified international 10/20 electrode placement system****…"*	Modified 10-20 system
**electrode_positions**	*"Seventeen recording electrodes (Ambu Neuroline disposable Ag/AgCl cup electrodes) were positioned according to the modified international 10/20 electrode placement system positioned at* ***Fz, Fp1, Fp2, F7, F8, Cz, Cpz, C3, C4, Cp3, Cp4, T3, T4, T5, T6, O1 and O2.*** *Reference and ground electrodes were placed at* ***FCz*** *and the chest respectively."* T3, T4, T5, T6 are standardised to T7, T8, P7, P8, respectively, according to the current version of the 10-10 system.	Fz, Fp1, Fp2, F7, F8, Cz, CPz, C3, C4, CP3, CP4, T7, T8, P7, P8, O1, O2, FCz
**eeg_data_loss_pct**	*"****Two*** *epochs were contaminated with gross movement artefact and were excluded from all subsequent analysis."* Percentage of data loss = 2/12*100 = 16.67%	16.67
**epoch_rej_method**	*"The EEG record was* ***assessed offline by a Clinical Physiologist and reviewed by a consultant Clinical Neurophysiologist****... The background EEG was as-sessed for continuity, movement artefact and muscle artefact."*	Subjective
**amplitude_threshold**	Not applicable	
**clinical_pain_scale**	Not applicable	
**non_eeg_recording**	"Infants were **video recorded** throughout the study (JVC, Everio hard disk camcorder) to assess the **sleep state** and detect **behav-ioural activity** such as, facial twitching, sucking and, eye and limb movements."	Video recording of facial expression, Video recording of sleep and behavioral state

49. Rebeccah Slater, Lorenzo Fabrizi, Alan Worley, Judith Meek, Stewart Boyd, Maria Fitzgerald. Premature infants display increased noxious-evoked neuronal activity in the brain compared to healthy age-matched term-born infants. NeuroImage. 2010;52(2):583-589. doi:10.1016/j.neuroimage.2010.04.253

Variable	*Data from publication*	*Data summary for review*
**publication_year**	2010	2010
**data_country**	*"Two groups of infants, recruited from the special care baby unit at the* ***Elizabeth Garrett Anderson and Obstetric Hospital****, participated in the study."*	United Kingdom
**sample_size**	*"The first group (no. of infants =* ***8****; age range: 37–40 weeks PMA at birth) were normal term infants who were less than 7*	17

	*postnatal days ('term-term'). The second group (no. of infants = 7; age range: 24–32 weeks PMA at birth) had been born prematurely and were studied when they reached a PMA equivalent to term ('prem-term')…**Two** additional infants who fulfilled the eligibility criteria were not included in this final sample of infants because movement artefact was identified in the EEG recordings."* Sample size = 8+7+2 = 17	
**pma_birth_avg**	Table 1: *"Mean PMA at birth (weeks) **26.9** (3.4); range 24.0–32.6 **38.6** (1.2); range 37.0–40.6"* Mean PMA at birth = (26.9+38.6)/2 = 32.75	32.75
**pma_study_avg**	Table 1: *"Mean PMA at time of study (weeks) **39.3** (1.2); range 37.6–41.4 **39.1** (1.2); range 37.7–41.0"* Mean PMA at study = (39.3+39.1)/2 = 39.2	39.2
**sex_male_pct**	Table 1: *"No. of males **3 6**"* Percentage of males = (3+6)/(8+7) = 9/15 = 60%	60
**sex_female_pct**	Percentage of females = 100 – percentage of males (see section 'sex_male_pct) = 100-60 = 40%	40
**pain_procedure**	*"We have measured evoked potentials generated by noxious clinically-essential **heel lances** in infants born at term (8 infants; born 37–40 weeks) and in infants born prematurely (7 infants; born 24–32 weeks) who had reached the same postmenstrual age (mean age at time of heel lance 39.2 ± 1.2 weeks)."*	Heel lance
**analgesic_intervention**	Not applicable	
**electrode_placement_method**	Electrode positions were listed and 'cap' was not mentioned, so we assume individual electrodes placement method were used.	Individual electrodes
**electrode_placement_system**	*"17 recording electrodes (Ambu Neuroline disposable Ag/AgCl cup electrodes) were positioned according to **the modified international 10/20 electrode placement system** positioned at Fz, Fp1, Fp2, F7, F8, Cz, CPz, C3, C4, CP3, CP4, T3, T4, T5, T6, O1 and O2."*	Modified 10-20 system
**electrode_positions**	*"17 recording electrodes (Ambu Neuroline disposable Ag/AgCl cup*	Fz, Fp1, Fp2, F7, F8, Cz, CPz, C3, C4, CP3, CP4, T7, T8, P7, P8, O1, O2, FCz

	*electrodes) were positioned according to the modified international 10/20 electrode placement system positioned at **Fz, Fp1, Fp2, F7, F8, Cz, CPz, C3, C4, CP3, CP4, T3, T4, T5, T6, O1 and O2**… Reference and ground electrodes were placed at **FCz** and the chest Respectively."* T3, T4, T5, T6 are standardised to T7, T8, P7, P8, respectively, according to the current version of the 10-10 system.	
**eeg_data_loss_pct**	*"The first group (no. of infants = **8**; age range: 37–40 weeks PMA at birth) were normal term infants who were less than 7 postnatal days ('term-term'). The second group (no. of infants = **7**; age range: 24–32 weeks PMA at birth) had been born prematurely and were studied when they reached a PMA equivalent to term ('prem-term')…**Two** additional infants who fulfilled the eligibility criteria were not included in this final sample of infants because movement artefact was identified in the EEG recordings."* Percentage of data loss = 2/(8+7+2) = 2/17 = 11.76%	11.76
**epoch_rej_method**	*"All data was free of movement artefact, which was defined as **a voltage change greater 100 µV that occurred within 100 ms** at either electrode site Cz or CPz."*	Objective
**amplitude_threshold**	*"All data was free of movement artefact, which was defined as **a voltage change greater 100 µV that occurred within 100 ms** at either electrode site Cz or CPz."*	Voltage change greater 100 microV that occurred within 100 ms
**clinical_pain_scale**	Not applicable	
**non_eeg_recording**	*"**Sleep states** were characterised using EEG criteria and **behavioural data from the video recording.** Regularity of **respiration** was measured using a movement transducer (Unimed) placed on the abdomen and **heart rate** was measured using lead 1 ECG. To check that the evoked activity was not generated by stimulus-triggered electro-oculographic activity the **video footage** and EEG recordings (activity at electrode site Fp1 and Fp2) were assessed to ensure that concurrent **eye movement** did not occur following the stimulation."*	Video recording of sleep and behavioral state, Video recording of facial expression, Respiratory rate, Heart rate

50. Rebeccah Slater, Laura Cornelissen, Lorenzo Fabrizi, Debbie Patten, Jan Yoxen, Alan Worley, Stewart Boyd, Judith Meek, Maria Fitzgerald. Oral sucrose as an analgesic drug for procedural pain in newborn infants: a randomised controlled trial. The Lancet. 2010;376(9748):1225-1232. doi:10.1016/S0140-6736(10)61303-7

Variable	***Data from publication***	***Data summary for review***
**publication_year**	2010	2010
**data_country**	*"In this double-blind, randomised controlled trial, 59 newborn infants at University College Hospital (London,* ***UK****) were randomly assigned to receive 0·5 mL 24% sucrose solution or 0·5 mL sterile water 2 min before undergoing a clinically required heel lance."*	United Kingdom
**sample_size**	Figure 2: From 59 baby randomised, 1 withdrawn, so 59-1 = 58 babies with EEG recording	58
**pma_birth_avg**	Table 1: *"PMA at birth (weeks)* ***39·8*** *(1·1)* ***39·8*** *(1·3)"* Table 1 reported data of babies with EEG analysed only. Average PMA at birth = (39.8+39.8)/2 = 39.8	39.8
**pma_study_avg**	Table 1: *"PMA at time of study (weeks)* ***40·1*** *(1·1)* ***40·3*** *(1·4)"* Table 1 reported data of babies with EEG analysed only. Average PMA at study = (40.1+40.3)/2 = 40.2	40.2
**sex_male_pct**	Table 1: *"Boys 11/20 (****55****%) 15/24 (****63****%)"* Table 1 reported data of babies with EEG analysed only. Percentage of males = (55+63)/2 = 59	59
**sex_female_pct**	Percentage of females = 100-average percentage of males = 100-59 = 41	41
**pain_procedure**	*"In this double-blind, randomised controlled trial, 59 newborn infants at University College Hospital (London, UK) were randomly assigned to receive 0·5 mL 24% sucrose solution or 0·5 mL sterile water 2 min before undergoing a clinically required* ***heel lance****."*	Heel lance
**analgesic_intervention**	*"In this double-blind, randomised controlled trial, 59 newborn infants at University College Hospital (London, UK) were randomly assigned to receive 0·5 mL 24%* ***sucrose*** *solution or 0·5 mL sterile water 2 min before undergoing a clinically required heel lance."*	Sucrose
**electrode_placement_method**	*"A neonatal EEG* ***cap*** *(WaveGuard EEG cap, Advanced NeuroTechnology, Enschede, Netherlands) was used to record EEG activity in the infants."*	Cap
**electrode_placement_system**	*"32 recording electrodes*	Modified 10-20 system

	were positioned according to ***the modified international 10/20 electrode placement system*** *at Fz, Fp1, Fp2, F3, F4, F7, F8, FT9, FT10, FC5, FC6, Cz, CPz, C3, C4, CP3, CP4, CP5, CP6, Pz, POz, P3, P4, P9, P10, PP07, PP08, T7, T8, Oz, O1, and O2."*	
**electrode_positions**	*"32 recording electrodes were positioned according to the modifi ed international 10/20 electrode placement system at* ***Fz, Fp1, Fp2, F3, F4, F7, F8, FT9, FT10, FC5, FC6, Cz, CPz, C3, C4, CP3, CP4, CP5, CP6, Pz, POz, P3, P4, P9, P10, PP07, PP08, T7, T8, Oz, O1, and O2****. Reference and ground electrodes were placed at* ***FCz*** *and the chest, respectively."* Electrode positions PP07 and PP08 are not found in 10-10 system and in manufacturer's guide, so they are adjusted to PO7 and PO8 after confirming with the corresponding author.	Fz, Fp1, Fp2, F3, F4, F7, F8, FT9, FT10, FC5, FC6, Cz, CPz, C3, C4, CP3, CP4, CP5, CP6, Pz, POz, P3, P4, P9, P10, PO7, PO8, T7, T8, Oz, O1, O2, FCz
**eeg_data_loss_pct**	Figure 2: *"**3** movement artifact in the EEG…**4** movement artifact in the EEG"* Percentage of data loss = number of babies excluded due to movement artifact / number of babies with EEG = (3+4)/(29+29) = 7/58 = 12.07%	12.07
**epoch_rej_method**	*"Infants were excluded from the analysis if technical failure occurred in the EEG recording or if movement artifact—defined as* ***a voltage change greater than 50 µV over 50 ms in 15 or more electrodes****— was identified in the alignment window."*	Objective
**amplitude_threshold**	*"Infants were excluded from the analysis if technical failure occurred in the EEG recording or if movement artifact—defined as* ***a voltage change greater than 50 µV over 50 ms in 15 or more electrodes****— was identified in the alignment window."*	Voltage change greater than 50 microV over 50 ms
**clinical_pain_scale**	*"Secondary measures were baseline behavioural and physiological measures, observational pain scores (**PIPP**), and spinal nociceptive refl ex withdrawal activity."*	PIPP/PIPP-R
**non_eeg_recording**	*"…**heart rate** measured with lead 1 electrocardiograph (ECG) electrodes placed on the chest.* ***Oxygen saturation*** *and heart rate were continuously*	Heart rate, Oxygen saturation, EMG, Video recording of facial expression, Video recording of sleep and behavioral state

	measured with a Nellcor N-560 transcutaneous pulse oximeter… ***EMG*** *activity, from 1 to 500 Hz, was recorded from the ipsilateral biceps femoris muscle with self-adhesive bipolar surface silver/silver chloride (Ag/AgCl) electrodes…* ***Facial expression*** *was recorded with a portable tripod-mounted camcorder."* and Figure 1.	

Roshni Mansfield (n=1 records)

39. Marianne Aspbury, Roshni Mansfield, Luke Baxter, Aomesh Bhatt, Maria Cobo, Sean Fitzgibbon, Caroline Hartley, Annalisa Hauck, Simon Marchant, Vaneesha Monk, Kirubin Pillay, Ravi Poorun, Marianne van der Vaart, Rebeccah Slater. Establishing a standardised approach for the measurement of neonatal noxious-evoked brain activity in response to an acute somatic nociceptive heel lance stimulus. Cortex. 2024;179:215-234. doi:10.1016/j.cortex.2024.05.023

Variable	Data from publication	Data summary for review
**publication_year**		2024
**data_country**	*"This study aimed to test whether a measure of noxious-evoked EEG activity called the noxious neurodynamic response function (n-NRF), that was originally derived in a sample of term-aged infants at the Oxford John Radcliffe Hospital, UK, in 2017, can reliably distinguish noxious from non-noxious events in two independent datasets collected at* ***University College London Hospital*** *and at* ***Royal Devon & Exeter Hospital.****"*	United Kingdom
**sample_size**	Fig.1(a) and (b), top boxes, n for heel lance recordings. 112+39=151.	151
**pma_birth_avg**	Table 2: "*Gestational age at birth (weeks)* ***36.6*** *(35.1, 39.3)* ***32.7*** *(29.9, 34.8)* ***37.4*** *(36.1, 39.9)* ***34.1*** *(32.9, 35.3)*" There are likely some participants that are overlapped between Dataset A and Dataset B from each site, so getting an accurate average of demographic data among all participants with EEG recorded was not possible. Furthermore, the demographic table only includes participants who were analysed, so did not cover all participants with EEG recorded. Here, we are getting the average of all reported groups in table 2, though this will not represent the true demographic of all participants with EEG recorded. (36.6 + 32.7 + 37.4 + 34.1)/4 = 35.2.	35.2
**pma_study_avg**	Table 2: "*Postmenstrual age at time of study (weeks)* ***37.4*** *(35.8, 39.8)* ***34.6*** *(32.7, 35.7)* ***38.1*** *(36.8, 40.4)* ***35.7*** *(35.2, 35.7)*" There are likely some participants that are overlapped between Dataset A and Dataset B from each site, so getting an accurate average of demographic data among all participants with EEG recorded was not possible. Furthermore, the demographic table only includes participants who were analysed, so did not cover all participants with EEG recorded. Here, we are getting the average of all reported groups in	36.45

	table 2, though this will not represent the true demographic of all participants with EEG recorded. (37.4 + 34.6 + 38.1 + 35.7)/4 = 36.45.	
**sex_male_pct**	Table 2: "*Male 35 (**49%**) 26 (**48%**) 16 (**46%**) 5 (**45%**)*" There are likely some participants that are overlapped between Dataset A and Dataset B from each site, so getting an accurate average of demographic data among all participants with EEG recorded was not possible. Furthermore, the demographic table only includes participants who were analysed, so did not cover all participants with EEG recorded. Here, we are getting the average of all reported groups in table 2, though this will not represent the true demographic of all participants with EEG recorded. (49 + 48 + 46 + 45)/4 = 47.	47
**sex_female_pct**	Table 2: "*Female 37 (**51%**) 28 (**52%**) 19 (**54%**) 6 (**55%**)*" There are likely some participants that are overlapped between Dataset A and Dataset B from each site, so getting an accurate average of demographic data among all participants with EEG recorded was not possible. Furthermore, the demographic table only includes participants who were analysed, so did not cover all participants with EEG recorded. Here, we are getting the average of all reported groups in table 2, though this will not represent the true demographic of all participants with EEG recorded. (51 + 52 + 54 + 55)/4 = 53.	53
**pain_procedure**	*"The noxious stimulus used throughout is the **heel lance**..."*	Heel lance
**analgesic_intervention**	Not applicable	
**electrode_placement_method**	*"**Eight EEG recording electrodes were positioned on the scalp** at Cz, CPz, C3, C4, FCz, T3, T4 and Oz according to the modified inter-national 10-20 System. Reference and ground electrodes were placed at Fz and the forehead respectively."*	Individual electrodes
**electrode_placement_system**	*"Eight EEG recording electrodes were positioned on the scalp at Cz, CPz, C3, C4, FCz, T3, T4 and Oz according to **the modified inter-national 10-20 System**. Reference and ground electrodes were placed at Fz and the forehead respectively."*	Modified 10-20 system

**electrode_positions**	*"Eight EEG recording electrodes were positioned on the scalp at **Cz, CPz, C3, C4, FCz, T3, T4** and **Oz** according to the modified international 10-20 System. Reference and ground electrodes were placed at **Fz** and the forehead respectively.".*	Cz, CPz, C3, C4, FCz, T3, T4, Oz, Fz
**eeg_data_loss_pct**	Fig.1: "*Lances excluded: Artefact: n=**9***" (from UCL), "*Lances excluded: Technical error: n=1*" (from Exeter). "*Eligible UCL dataset: Heel lance recordings: n=**106**. Eligible Exeter dataset: Heel lance recordings: n=**39***). EEG data loss percentage = 9/(106+39) = 6.21%.	6.21
**epoch_rej_method**	*"Traces with signal **amplitude greater than ±150 mV** were rejected. Traces with a **greater than 100 mV difference between maximum and minimum baseline amplitude in the pre-stimulus period** were rejected. Traces with **visually apparent artefact falling within these pre-defined amplitude bounds** were also rejected (e.g., gross movement artefact, muscle artefact, line noise or other technical artefact)."*	Mixed
**amplitude_threshold**	*"Traces with signal **amplitude greater than ±150 mV** were rejected. Traces with a **greater than 100 mV difference between maximum and minimum baseline amplitude in the pre-stimulus period** were rejected. Traces with visually apparent artefact falling within these pre-defined amplitude bounds were also rejected (e.g., gross movement artefact, muscle artefact, line noise or other technical artefact)."*	Amplitude greater than ±150 mV, greater than 100 mV difference between maximum and minimum baseline amplitude in the pre-stimulus period
**clinical_pain_scale**	Not applicable	
**non_eeg_recording**	"An ECG electrode was placed on the left clavicle to record **heart rate**."	Heart rate

Safa Talebi (n=1 records)

52. Safa Talebi, Javad Frounchi, Behzad Mozaffari Tazehkand. A novel channel selection approach for human neonate's pain EEG data analysis. SIViP. 2025;19(5):364. doi:10.1007/s11760-025-03934-x

Variable	Data from publication	Data summary for review
**publication_year**		2025
**data_country**	*"In this study, we used some of the EEG data collected in **the Elizabeth Garrett Anderson Obstetric Wing, University College London Hospital (UCLH)**."*	United Kingdom
**sample_size**	*"...we included the data of **18** neonates in this study."*	18
**pma_birth_avg**	Not provided	
**pma_study_avg**	Not provided	
**sex_male_pct**	Not provided	
**sex_female_pct**	Not provided	
**pain_procedure**	*"The stimulations were performed using a **lancet**... we included the data related to stimulation through the left **heel** in this study"*	Heel lance
**analgesic_intervention**	Not applicable	
**electrode_placement_method**	Electrode positions were listed and 'cap' was not mentioned, so we assume individual electrodes placement method were used.	Individual electrodes
**electrode_placement_system**	*"The EEG data recording was performed through 20 electrodes O1, O2, T7, T8, F7, F3, F4, FCz, F8, P7, P8, TP9, TP10, POz, C3, Cz, C4, CP3, CPz CP4, which were placed on the head according to a **modified international 10–10 electrode placement system**"*	Modified 10-10 system
**electrode_positions**	"The EEG data recording was performed through 20 electrodes **O1, O2, T7, T8, F7, F3, F4, FCz, F8, P7, P8, TP9, TP10, POz, C3, Cz, C4, CP3, CPz CP4**... The reference electrode was positioned in **Fz**, and the ground electrode was positioned in FC1 or FC2 (depending on the position of the neonate)."	O1, O2, T7, T8, F7, F3, F4, FCz, F8, P7, P8, TP9, TP10, POz, C3, Cz, C4, CP3, CPz, CP4, Fz
**eeg_data_loss_pct**	No data were mentioned to be excluded due to artefact. *"Overall, the data of the neonates whose PIPP scores are more than 6 (moderate pain and severe pain) and whose pain stimulations have been performed through the left heel have been included in this study."*	0
**epoch_rej_method**	Not applicable. No epoch rejection method was mentioned in the paper.	
**amplitude_threshold**	Not applicable. No epoch rejection method was mentioned in the paper.	
**clinical_pain_scale**	*"The **PIPP** score is also available for some of these data. We considered the data that PIPP scores are reported for that."*	PIPP/PIPP-R
**non_eeg_recording**	Not applicable	

Simon Marchant (n=1 records)

54. Simon Marchant, Marianne van der Vaart, Kirubin Pillay, Luke Baxter, Aomesh Bhatt, Sean Fitzgibbon, Caroline Hartley, Rebeccah Slater. A machine learning artefact detection method for single-channel infant event-related potential studies. J Neural Eng. 2024;21(4):046021. doi:10.1088/1741-2552/ad5c04

Variable	Data from publication	Data summary for review
**publication_year**		2024
**data_country**	*"These data were collected as part of ongoing studies in John Radcliffe Hospital (Oxford, **UK**)... These data were collected as part of Petal, a multi-site clinical trial conducted in the John Radcliffe Hospital (Oxford, **UK**) and the Royal Devon and Exeter Hospital (Exeter, **UK**)"*	United Kingdom
**sample_size**	*"The 'Oxford multimodal' dataset consists of 410 unique EEG epochs from **160** infants 28–43 weeks postmenstrual age... we also tested the model in a clinical trial dataset. The 'Petal' dataset consists of 316 unique epochs from **108** infants 35–42 weeks postmenstrual age"* Total sample size = 160 + 108 = 268	268
**pma_birth_avg**	Average is not provided; only provided in range. *"Infants included were all born between 23–43 weeks gestational age."*	
**pma_study_avg**	Average is not provided; only provided in range. *"At the time that EEG recordings were made, infants were aged 28–43 weeks postmenstrual age."*	
**sex_male_pct**	Not provided	
**sex_female_pct**	Not provided	
**pain_procedure**	*"EEG was recorded in rest and in response to experimental auditory, visual, tactile, vibrotactile and mild noxious stimulation, as well as in response to clinically required **heel lances**."*	Heel lance
**analgesic_intervention**	Not applicable	
**electrode_placement_method**	*"EEG recording electrodes (AmbuNeuroline disposable Ag/AgCl cup electrodes)* ***were positioned on the scalp*** *according to the modified international 10–20 System, with reference and ground electrodes at Fz and the forehead respect-*	Individual electrodes

	ively."	
**electrode_placement_system**	*"EEG recording electrodes (AmbuNeuroline disposable Ag/AgCl cup electrodes) were positioned on the scalp according to* ***the modi-fied international 10–20 System****, with reference and ground electrodes at Fz and the forehead respect-ively."*	Modified 10-20 system
**electrode_positions**	*"EEG recording electrodes (AmbuNeuroline disposable Ag/AgCl cup electrodes) were positioned on the scalp according to the modi-fied international 10–20 System, with reference and ground electrodes at* ***Fz*** *and the forehead respect-ively... Studies used a varying number of channels, but always included* ***Cz*** *and this is the channel from which we take EEG for this study."*	Fz, Cz
**eeg_data_loss_pct**	Not applicable. This study's aim is to create and test an automated method to detect artefact, so no study was excluded from analysis due to artefacts. *"The aim of this study was to create and test an automated method of detecting artefact in single-channel 1500 ms epochs of infant EEG."*	
**epoch_rej_method**	Not applicable. This study's aim is to create and test an automated method to detect artefact, so no study was excluded from analysis due to artefacts. *"The aim of this study was to create and test an automated method of detecting artefact in single-channel 1500 ms epochs of infant EEG."*	
**amplitude_threshold**	Not applicable. This study's aim is to create and test an automated method to detect artefact, so no study was excluded from analysis due to artefacts. *"The aim of this study was to create and test an automated method of detecting artefact in single-channel 1500 ms epochs of infant EEG."*	
**clinical_pain_scale**	Not applicable	
**non_eeg_recording**	Not applicable	

Sofie Nilsson (n=1 records)

55. Sofie Nilsson, Anton Tokariev, Timo Vehviläinen, Vineta Fellman, Sampsa Vanhatalo, Elisabeth Norman. Depression of cortical neuronal activity after a low-dose fentanyl in preterm infants. Acta Paediatrica. 2025;114(1):109-115. doi:10.1111/apa.17411

Variable	***Data from publication***	***Data summary for review***
**publication_year**		2025
**data_country**	"*The NeoFent study, a part of the EU- funded FP7- HEALTH- 2007B NeoOpioid project (no. 223767), was a two- centre study in* ***Sweden****...*"	Sweden
**sample_size**	*"****Fifteen*** *infants received fentanyl* ***prior to a skin breaking proce-dure*** *(0.5 µg/kg), one infant before tracheal intubation (2 µg/kg over 5 min) and one infant was excluded due to technical problems when opening the EEG file."*	15
**pma_birth_avg**	"*Fifteen infants* ***born at median 26.4 gestational weeks*** *(range 23.3–34.1), with a birth weight 740 grams (530–1420) and postnatal age 7 days (5–11) received fentanyl 0.5 or 2 µg/kg intravenously before a skin- breaking procedure or tracheal intubation, respectively."*	26.4
**pma_study_avg**	"*Fifteen infants* ***born at median 26.4 gestational weeks*** *(range 23.3–34.1), with a birth weight 740 grams (530–1420) and* ***postnatal age 7 days (5–11)*** *received fentanyl 0.5 or 2 µg/kg intravenously before a skin- breaking procedure or tracheal intubation, respectively."* 7 days = 1 week, so average PMA at study = 26.4 + 1 = 27.4 week	27.4
**sex_male_pct**	Not provided	
**sex_female_pct**	Not provided	
**pain_procedure**	*"To assess PK and pharmacodynamics (PD), infants born before 35 gestational weeks (GW) with a postnatal age (PNA) of 0–28 days were eligible if in need of a skin- breaking procedure (****an intravenous, intraarterial cannula or a short injection of local anaesthetics for a peripheral insertion of a central venous line****), endotracheal suction or an endotracheal intuba-tion."*	Venipuncture, Arterial puncture, Injection of local anaesthetics
**analgesic_intervention**	*"Fifteen infants received* ***fentanyl*** *prior to a skin breaking proce-dure (0.5 µg/kg), one infant before tracheal intubation (2 µg/kg over 5 min) and one infant was excluded due to technical problems when*	Fentanyl

	opening the EEG file."	
**electrode_placement_method**	Electrode positions were listed and 'cap' was not mentioned, so we assume individual electrodes placement method were used.	Individual electrodes
**electrode_placement_system**	Not provided	
**electrode_positions**	"*For the aEEG/EEG registration, six electrodes were used: two frontal (**F3 and F4**), two parietal (**P3 and P4**) and two reference electrodes placed at **Fz** (frontal midline) and **Cz** (central midline), respectively."*	F3, F4, P3, P4, Fz, Cz
**eeg_data_loss_pct**	*"**Fifteen** infants received fentanyl **prior to a skin breaking procedure (0.5 µg/kg),** one infant before tracheal intubation (2 µg/kg over 5 min) and one infant was excluded due to technical problems when opening the EEG file... **Fourteen infants received the 0.5 µg/kg fentanyl dose, and one infant received 2 µg/kg** (Table 1). In the visual observation of the time trends, there were no clear visual changes between pre-- and post-- drug values in neither the absolute time trends, nor the relative time trends."* Among the 15 infants that received skin-breaking procedure with 0.5 mcg/kg fentanyl, the text implies that only 14 was analysed. The reason for exclusion for the one infant was not provided.	
**epoch_rej_method**	*"First the recordings were **reviewed visually for data quality** as well as for any obvious changes in the background activity or aEEG trends. Then, the signals were exported into European Data Format in the recording montage. Further analyses were done using a custom- made Matlab script which included preprocessing **for automated artefact detection and removal**."*	Mixed
**amplitude_threshold**	Not provided	
**clinical_pain_scale**	Not applicable	
**non_eeg_recording**	Not applicable	

Sonya Wang (n=1 records)

6. Sonya Wang. Effects of Music Based Intervention (MBI) on Pain Response and Neurodevelopment in Preterm Infants.; 2020. https://clinicaltrials.gov/study/NCT04286269

Variable	*Data from publication*	*Data summary for review*
**publication_year**	*"Study Start (Actual)*<br>***2020**-11-20"*	2020
**data_country**	*"This study has 1 location*<br>***United States***<br>*Minnesota Locations*<br>*Minneapolis, Minnesota, United States, 55455*<br><br>*University of Minnesota"*	United States of America

Vineta Fellman (n=1 records)

7. Vineta Fellman. NeoFent-I Study; Fentanyl Treatment in Newborn Infants; a Pharmacokinetic, Pharmacodynamic and Pharmacogenetic Study.; 2012. https://clinicaltrials.gov/study/NCT03897452

Variable	*Data from publication*	*Data summary for review*
**publication_year**	*"Study Start (Actual)<br>**2012**-11"*	2012
**data_country**	*"This study has 1 location<br>**Sweden**<br>Lund, Sweden, 221 85<br><br>Neonatal Intensive Care Unit, Skåne University Hospital"*	Sweden

Researcher co-authorship network clusters

Supplementary table 6: Researcher co-authorship network

Cluster 1	Cluster 2	Cluster 3	Cluster 4	Cluster 5	Cluster 6	Cluster 7	Cluster 8	Cluster 9	Cluster 10	Cluster 11	Cluster 12	Cluster 13
adam shriver	alexandra key	anton tokariev	andreea pavel	carlo dani	aaron newman	eilon shany	chinami hanai	amir avnit	hisham abdelsami awad	guy dumont	behzad mozaffari tazehkand	mohammad reza daliri
alan worley	ann stark	elisabeth norman	anna powell	caterina coviello	arlene jiang	ivan cepeda	kiyoko yokoyama	behnood gholami	l taher bassiouny	liisa holsti	javad frounchi	reyhane shafiee
amraj dhami	arnaud jeanvoine	ingmar rosen	caoimhe howard	cesarina cossu	bonnie stevens	leonel selnovik	ming huang	jean-michel roué	rania mohamed abdou	shahbaz askari	safa talebi	
amy hoskin	caitlin kjeldsen	karin stjernqvist	daragh finn	clara lunardi	britney benoit	michael friger	nusreena hohsoh	kanwaljeet anand	sahar hassanein	zoya bastany		
amy lee	carrie mccoy menser	lena hellstrom-westas	elena pavlidis	giovanna bertini	celeste johnston	neta maimon	osuke iwata	wassim haddad				
ana alarcon	daniel france	marit lundblad	eugene dempsey	maria bastianelli	margot latimer	ruth grunau	tomoko suzuki					
andrew king	don wilkes	mats blennow	farah abu dhais	sara cavaliere	marsha campbell-yeo	shlomo gilat						
annalisa hauck	kathleen adderley	ove okland	geraldine boylan	silvia lori	ruth martin-misener							
annika grabbe	ken wilkens	paul castillo	john o’toole	simona montano	tim bardouille							
aomesh bhatt	lance relland	per-arne lonnqvist	vicki livingstone	simonetta gabbanini								
caroline hartley	lelia emery	sampsa vanhatalo										
chetan patel	maeve mcloughlin	sofie nilsson										
daniel crankshaw	melissa moore-clingenpeel	timo vehviläinen										
debbie patten	nathalie maitre	vineta fellman										
deniz gursul	olena chorna											
dominic wilkinson	rachelle srinivas											
edmund juszczak	stephen bruehl											
eleri adams												
eugene duff												
fiona moultrie												
foteini andritsou												
francis mcglone												
franziska denk												
gabriela schmidt mellado												
gabrielle green												
gareth hathway												
gemma williams												
hemani patel												
ilana shiff												
ismini panayotidis												
jan yoxen												
jane norman												
jean yong												
jennifer bell												
judith meek												
kelly brotherhood												
kimberley whitehead												
kirubin pillay												
laura cornelissen												
laura jones												
lorenzo fabrizi												
luke baxter												
madeleine verriotis												
maria cobo												
maria fitzgerald												
maria laudiano-dray												
marianne aspbury												
marianne van der vaart												
marina freire												
mariska peck												
miranda buckle												
mohammed rupawala												
nader raafat												
oana bucsea												
ravi poorun												
rebecca pillai riddell												
rebeccah slater												
ria evans fry												
richard rogers												
robert cooper												
roshni mansfield												
ryan purdy												
sean fitzgibbon												
sezgi goksan												
shellie robinson												
sheryl ledwidge												
simon marchant												
siân roberts-holdridge												
sofia olhede												
stewart boyd												
susannah walker												
vaneesha monk												
xiaogang wang												
